# Annotation of gene loci and analysis of expression diversity in sheep immunoglobulin

**DOI:** 10.3389/fimmu.2025.1643380

**Published:** 2025-08-29

**Authors:** Mingli Wu, Fuwen Chen, Xiaoqin Tang, Jingxuan Li, Haidong Zhao, Yuelang Zhang

**Affiliations:** ^1^ Guangxi Key Laboratory of Brain and Cognitive Neuroscience, Guilin Medical University, Guilin, Guangxi, China; ^2^ Hainan Institute of Zhejiang University, Sanya, Hainan, China; ^3^ College of Animal Science and Technology, Northwest A&F University, Shaanxi, China; ^4^ College of Intelligent Medicine and Biotechnology, Guilin Medical University, Guilin, Guangxi, China

**Keywords:** ovis aries, immunoglobulin heavy chain, immunoglobulin light chain, immunoglobulin gene loci, expression diversity

## Abstract

As an important livestock species, sheep exhibit remarkable environmental adaptability. Immunoglobulins, expressed by B cells, are among the most crucial effector molecules in adaptive immunity. However, systematic research on the structure and expression diversity of the sheep immunoglobulins gene loci remains limited. This study annotated the sheep IgH, Igκ, and Igλ loci based on the sheep genome assembly (ARS-UI_Ramb_v3.0). The sheep IgH is located on chromosome 18 and comprises 22 VH, 4 DH, and 6 JH. The Igκ is on chromosome 3, containing 18 Vκ and 4 Jκ. The Igλ is situated on chromosome 17 and consists of 128 Vλ and 3 Jλ. Rearranged IgH, Igκ, and Igλ sequences were obtained from sheep spleen using 5’ RACE PCR. Following PE300 high-throughput sequencing, we analyzed the diversity of V, D, J expression diversity, V(D)J recombination, junctional diversity, and somatic hypermutation in the rearranged sequences. For IgH rearrangement, 4 VH, 4 DH, and 2 JH gene segments were utilized, generating 26 distinct rearrangement types. Igκ rearrangement employed 5 Vκ and 3 Jκ gene segments, resulting in 13 rearrangement types. Igλ rearrangement involved 26 Vλ and 2 Jλ gene segments, producing 28 rearrangement types. Average length of sheep CDR3H is 44 bp (maximum 66 bp), CDR3κ averages 27 bp (maximum 48 bp), and CDR3λ averages 30 bp (maximum 47 bp). N-nucleotide additions contributed more significantly to CDR3 diversity than P-nucleotides in both Igκ and Igλ rearrangements. Simultaneously, 3’ V-deletion and 5’ J-deletion further enriched CDR3 diversity. SHM, especially the hotspot mutation motifs, enriches the diversity caused by the V gene segments. Thus, sheep enrich immunoglobulin diversity through both junctional diversity-driven CDR3 diversification and high-intensity SHM. This study expands our understanding of the sheep immunoglobulin gene loci and their expression diversity, providing theoretical foundation for research on immunoglobulin gene evolution within the Bovidae family.

## 1 Introduction

The immune system constitutes a vital defense mechanism through which multicellular organisms recognize and eliminate pathogenic antigens to maintain homeostasis. This complex biological system is functionally divided into two principal components: the innate (non-specific) immune system and the adaptive (specific) immune system (1). The innate immune system serves as the first-line defense in vertebrates, providing rapid non-specific responses through multiple protective mechanisms. These include mechanical barriers such as mucosal epithelia in the respiratory tract, gastrointestinal barriers, urogenital tract defenses, and molecular antimicrobial factors. In contrast, the adaptive immune system mediates antigen-specific responses characterized by immunological memory. This evolutionarily advanced system, which emerged approximately 500 million years ago through the development of antigen receptor gene rearrangement mechanisms, demonstrates two distinct effector pathways: humoral immunity mediated by antibody-producing plasma cells (differentiated from B lymphocytes) and cell-mediated immunity executed by cytotoxic T lymphocytes (2). The adaptive immune response exhibits antigen-specific recognition, clonal expansion, and long-term immunological memory formation.

Immunoglobulins are composed of four heterodimeric polypeptide chains, comprising two identical immunoglobulin heavy chain (IgH) and two identical light chain (IgL). X-ray diffraction phase analysis revealed that the heterodimers, connected by variable numbers of interchain disulfide bonds, adopt a characteristic Y-shaped configuration corresponding to a single immunoglobulin monomer. Both polypeptide chains contain variable (V) and constant (C) regions (3). The C-terminal domains, characterized by conserved amino acid sequences, constitute the constant region, whereas the N-terminal domains exhibit substantial sequence variation within approximately 110 amino acid residues, defining the variable region. The variable domains are designated as the heavy chains variable region (VH) and light chains variable region (VL), respectively. Structurally, VH comprises one-quarter of the IgH polypeptide, while VL accounts for half of the IgL chain. Both variable domains contain three frame regions (FRs) interspersed with three complementarity determining regions (CDRs) (4–6). The FRs, which are evolutionarily conserved, function primarily in maintaining the structural integrity of the immunoglobulin molecule. In contrast, CDRs demonstrate exceptional sequence diversity, directly mediating antigen recognition specificity and binding affinity. These hypervariable segments are positioned at amino acid residues H31-H35B/L24-L34 (CDR1), H50-H65/L50-L56 (CDR2), and H95-102/L89-L97 (CDR3), with CDR3 exhibiting the highest degree of sequence variation (7). Immunoglobulin isotypes differ in the domain organization of their constant regions. In mammals, five major isotypes have been characterized: IgM, IgD, IgG, IgE, and IgA (8). IgD, IgG, and IgA contain three CH domains, whereas IgM and IgE contain four CH domains. Between the CH1 and CH2 domains of IgD, IgG, and IgA, there is a proline-rich peptide segment approximately 20 amino acid residues in length. This region exhibits a high degree of spatial flexibility and is known as the hinge region (9, 10). Notably, the IgL constant region (CL) contains a single constant domain.

In vertebrate organisms, the germline-encoded immunoglobulin gene repertoire remains relatively fixed, whereas environmental pathogens exhibit continuous evolutionary diversification. To address this immunological challenge, vertebrates have evolved sophisticated molecular mechanisms to generate antibody diversity. Key mechanisms include V(D)J recombination, gene conversion (GCV), somatic hypermutation (SHM), and class switch recombination (CSR), which collectively enable the production of diverse antigen-specific immunoglobulins capable of neutralizing evolving pathogens (11–13). While CSR modifies the constant region of antibodies, the other three mechanisms enrich antibody diversity by increasing the diversity of the variable regions (14).

The V(D)J recombination process is initiated by recombination-activating gene 1/2 (RAG1/RAG2) complexes in conjunction with high mobility group (HMG) proteins. These molecular complexes precisely recognize conserved recombination signal sequences (RSS) flanking V, diversity (D), and joining (J) gene segments. The RAG endonuclease subsequently introduces double-strand breaks (DSBs) at specific RSS sites, followed by DNA repair mechanisms that mediate segment rearrangement through non-homologous end joining (15, 16). Furthermore, combinatorial assembly of two identical IgH with two identical IgL generates additional structural variation through heterodimer pairing.

To neutralize certain highly variable antigens, in addition to the four classical mechanisms of immunoglobulin diversity generation, there exist several non-canonical pathways. These atypical diversity-generating mechanisms hold significant value in both scientific research and medical applications (17). In addition to conventional antibodies composed of paired IgH and IgL, certain species have evolved the capability to produce heavy chain homodimers. Notably, camelids generate heavy-chain-only antibodies (HCAbs) that lack IgL and the CH1 domain in their IgH, while cartilaginous fish possess immunoglobulin new antigen receptors (IgNARs) characterized by a single variable domain followed by five constant domains. These atypical antibodies exhibit distinctive features including compact molecular dimensions, enhanced thermal stability, and distinct paratope configurations, thereby significantly expanding the antibody repertoire diversity in both camelids and elasmobranchs (18, 19). In bovines, a limited subset of rearranged IgH sequences exhibit exceptionally long CDR3H domains exceeding 70 amino acid residues, significantly exceeding those observed in the longest CDR3H regions of camelid IgGs and shark IgNARs. X-ray crystallographic analyses of five bovine antibodies featuring these ultralong CDR3H motifs reveal a distinctive architecture characterized by two structural components: a disulfide-bond-stabilized “knob” domain supported by an elongated β-ribbon “stalk” configuration (20). The ultra-long CDR3H region is generated through the involvement of the DH8 gene segment. The germline-encoded DH8 gene contains an even number of cysteine (Cys) residues, where alternative pairing configurations between different Cys residues significantly enhance immunoglobulin diversity. Notably, the DH8 segment harbors abundant activation-induced cytidine deaminase (AID) hotspot motifs. SHM modifies both the spatial distribution and numerical count of specific Cys residues, thereby further diversifying the immunoglobulin repertoire to a greater extent. The molecular mass of ultralong CDR3H is even lower than that of HCAb or IgNARs, potentially enabling its independent binding to epitopes inaccessible to conventional antibodies (21). This distinctive structural characteristic of ultralong CDR3H therefore provides a novel strategy for engineering antibodies targeting challenging antigenic targets. By substituting the original pivot domain with alternative proteins or peptides (e.g., GCSF, EPO, or CXCR4), functional fusion proteins with desired pharmacological properties can be generated (22–24). Given the structural versatility of bovine ultralong CDR3H, it establishes a robust structural platform for developing next-generation diagnostic tools, therapeutic agents, vaccine candidates, and immunomodulatory compounds.

Bovidae, as one of the primary categories of domesticated livestock in contemporary human society, encompasses major working or meat-producing species such as cattle, yaks, buffalo, goats, and sheep. Previous research on the structural diversity of immunoglobulins within this taxon has been constrained by sequencing technologies and genome assembly methods. To date, comprehensive characterization has only been achieved in Holstein cattle and yaks—revealing a unique evolutionary strategy in cattle and yaks that generates immunoglobulin diversity through ultra-long CDR3H domains, a biological phenomenon specific to domestic cattle among mammals (25). Nevertheless, current understanding of the immunoglobulin gene structure and expression diversity in Bovidae remains incomplete, limiting the comprehension of immunoglobulin evolutionary mechanisms within this family. To supplement evidence for immunoglobulin gene evolution in Bovidae, this study integrates genome alignment strategies with high-throughput sequencing technologies. Employing comparative genomics approaches, we systematically analyze the immunoglobulin gene structure and expression diversity in sheep. This expands the current framework for understanding immunoglobulin structural organization and diversity generation mechanisms in Bovidae, providing an important theoretical foundation for advancing disease prevention and immune intervention strategies in these animals.

## 2 Materials and methods

All experimental procedures were conducted in accordance with the Regulations on the Administration of Laboratory Animals approved by the State Council of the People’s Republic of China.

### 2.1 Animal model

Three healthy adult sheep (2 years old) were utilized in this study. The animal slaughter procedures and spleen sample collection were conducted at a certified slaughterhouse. Following collection, the spleen specimens were immediately preserved in liquid nitrogen for transport to the laboratory (Servicebio, Wuhan, China).

### 2.2 Structural analysis of immunoglobulin gene in sheep

Download the VH, DH, JH, Vλ, Jλ, Vκ, Jκ fragments and constant region μ, δ, α, γ, ϵ, λ, κ sequences of human, mouse, sheep and cattle from NCBI (http://www.ncbi.nlm.nih.gov) and the IMGT (https://www.imgt.org/). The locations of VH, DH, JH, Vλ, Jλ, Vκ, Jκ, μ, δ, α, γ, ϵ, λ, κ genes in sheep genome were searched by BLAST, and the potential D and J fragments were searched by FUZZNUC (http://embossgui.sourceforge.net/demo/fuzznuc.html) for RSS sequences conforming to the 12/23 rule. Immunoglobulin IgH, Igλ and Igκ gene loci were mapped. The naming is based on the similarity of the sequence to the sequences in the IMGT database.

### 2.3 RNA Isolation and 5’ RACE

Total RNA was isolated from spleen tissues of three adult sheep using TRIzol^®^ Reagent (Takara Bio, Dalian, China) according to the manufacturer’s protocol. RNA purity and concentration were determined by spectrophotometric measurement using a NanoDrop 1000 system (Thermo Fisher Scientific, USA). Qualified RNA samples were aliquoted and preserved at −80°C for subsequent experiments.

The 5’RACE reactions was performed using the SMARTer RACE 5’/3’ Kit (Takara Bio, Dalian, China) with the following procedure: A master mixture containing 1 μl 5’ RACE CDS Primer A (12 μM), 1 μg total RNA (1 μg/μl), and 9 μl nuclease-free H_2_O was prepared. Mix contents and spin the tubes briefly in a microcentrifuge. Incubate tubes at 72°C for 3 minutes, then cool the tubes to 42°C for 2 minutes. After cooling, spin the tubes briefly for 10 seconds at 14,000 x g to collect the contents at the bottom. Subsequently, to just the 5’-RACE cDNA synthesis reaction(s), add 1 µl of the SMARTer II A Oligonucleotide (24 μM) per reaction. The reaction system was supplemented with 4 μL 5× First-Strand Buffer, 0.5 μL DTT (100 mM), 1 μL dNTPs (20 mM), 0.5 μL RNase Inhibitor (40 U/μl), and 2 μL SMARTScribe Reverse Transcriptase (100 U). Mix the contents of the tubes by gently pipetting, and spin the tubes briefly to collect the contents at the bottom. Incubate the tubes at 42°C for 90 minutes in an air incubator or a hot-lid thermal cycler. Heat tubes at 70°C for 10 minutes. Dilute the first-strand cDNA synthesis reaction product with 240 μl Tricine-EDTA Buffer and stored at −20°C.

### 2.4 Cloning of the expressed sheep IgH, Igλ and Igκ fragments by 5’ race PCR and sequencing

The amplification of sheep IgH, Igλ, and Igκ genes was performed using SeqAmp DNA Polymerase (Takara Bio, Dalian, China; Cat. No. 638504) through 5’-RACE PCR. The universal forward primer (UPM: 5’-AAGCAGTGGTATCAACGCAGAGT-3’) was provided with the SMARTer RACE cDNA Amplification Kit. Gene-specific reverse primers (GSPs) were designed to anneal to the 5’ end of the constant regions: IgH-R (5’-ACACCAGGGGGAAGACTCTCGGG-3’), Igκ-R (5’-GAAGAGGAAGACGGATGGCT-3’), and Igλ-R (5’-GTGACCGAGGGTGCGGACTTG-3’). The RACE-PCR reaction system was assembled in a 200 μL tube (NEST, Wuxi, Chain) containing the following components: 25 μL PCR-grade H_2_O, 1 μL SeqAmp DNA Polymerase, 2.5 μL 5’-RACE-Ready cDNA, 5 μL 10× 3’ UPM short primer, 1 μL 5’ GSP (10 μM), and 15.5 μL nuclease-free H_2_O. The mixture was thoroughly mixed by gentle pipetting prior to thermal cycling. Amplification was performed under the following conditions: initial denaturation at 94°C for 30 sec; 25 cycles of denaturation (94°C, 30 sec), annealing (52°C, 30 sec), and extension (72°C, 1 min); followed by a final extension at 72°C for 5 min. Amplification products were subsequently purified and subjected to high-throughput sequencing analysis (Sangon Biotech, Shanghai, China).

### 2.5 Analysis of expression diversity

IgH recombination diversity and junctional diversity were analyzed using the International IMGT (26). All sequences were clustered according to their germline V gene segments assignments, with each cluster representing a distinct germline V gene segments. For subsequent analyses, the sequences were partitioned into distinct genomic segments based on germline reference alignments, including V gene segments, D gene segments, J gene segments, CDR3 sequences. Clonal sequences exhibiting maximal germline identity across Framework Regions 1-3 (FR1-FR3) were selected for detailed examination of VH expression patterns and nucleotide substitution profiles. Mutation sites and their corresponding frequencies were systematically analyzed using MEGA version 7.0 (Molecular Evolutionary Genetics Analysis) and Microsoft Excel software, with sequence alignments performed against established germline references. The MEGA software (version 7.0) was employed to identify V(D)J gene segment usage patterns and assess recombination diversity in immunoglobulin gene recombination. Based on cluster analysis results, SHM frequencies within FRs and CDRs were quantified through alignment of rearranged sequences with corresponding germline V segment reference genomes.

### 2.6 Sequencing and bioinformatics analysis

High-throughput sequencing and Sanger sequencing were performed by

Sangon Biotech (Shanghai) Co., Ltd (Shanghai, China). The experimental workflow comprised the following key steps (27):

1. 5’ RACE amplification: PCR products were purified and submitted for sequencing following experimental protocols.
2. Library preparation: PCR products underwent quality assessment using agarose gel electrophoresis. Qualified samples were processed for library construction using Illumina-compatible bridge PCR primers, followed by DNA fragment purification.
3. Sequencing execution: Library concentration was quantified via Qubit 3.0 fluorometer (Thermo Fisher Scientific). After quality validation, libraries were sequenced on an Illumina platform using a PE300 paired-end sequencing strategy to generate 300 bp paired-end reads.
  Bioinformatics processing:

(1) Raw reads were assembled and filtered through the following pipeline:
- Removal of sequences lacking amplification primers;
- Immunoglobulin recombination sequences with complete structures exceed 400 bp (barcode + upstream primer + leader region + V region + J region + partial C region + downstream primer > 400 bp). Therefore, reads shorter than 400 bp were discarded as they may represent incomplete recombination sequences;
(2) V(D)J gene alignment:
- IMGT/HighV-QUEST was employed for reference alignment against immunoglobulin V, D, and J gene databases;
- Sequences with valid V/D/J pairing were retained for downstream analysis;
(3) Gene annotation:
- IMGT/HighV-QUEST was utilized for partitioning sequences into V, D, J, and junction regions;
(4) Somatic hypermutation (SHM) analysis:
- IMGT/HighV-QUEST was employed to calculate SHM frequencies for each experimental group according to the analytical protocol established in Step (2);
(5) Junction characterization:
- IMGT/HighV-QUEST was utilized to quantitatively analyze the CDR3 length profiles and enumerate the nucleotide insertion patterns of N (non-templated) and P (palindromic) sequences.

5. Immunoglobulin repertoire analysis:

(1) V(D)J subgroup usage:
- subgroup distribution was quantified without duplicate removal
- Results expressed as percentage of total sequences
(2) CDR3 profiling:
- CDR3 length distributions were determined for each sample
(3) N/P nucleotide analysis:
- Base composition and positional distribution were quantified
(4) SHM quantification:
- After replacing the IMGT germline template V gene sequences with the template sequences submitted in this study (**Supplementary 1**), SHM analysis was then performed.
Mutation frequency calculated as:
SHM frequency = (number of mutated bases)/(total sequenced bases) × 100%
- AID hotspot motifs mutation count: the mutation count of C/G within the AID hotspot motifs WRCY/RGYW
(5) Mutation spectrum analysis:
- Transition/transversion patterns were categorized
- Mutation frequencies converted to percentage of total observed mutations

## 3 Results

### 3.1 Schematic structure of the genomic organization of sheep IgH and IgL

**Figure 1:**
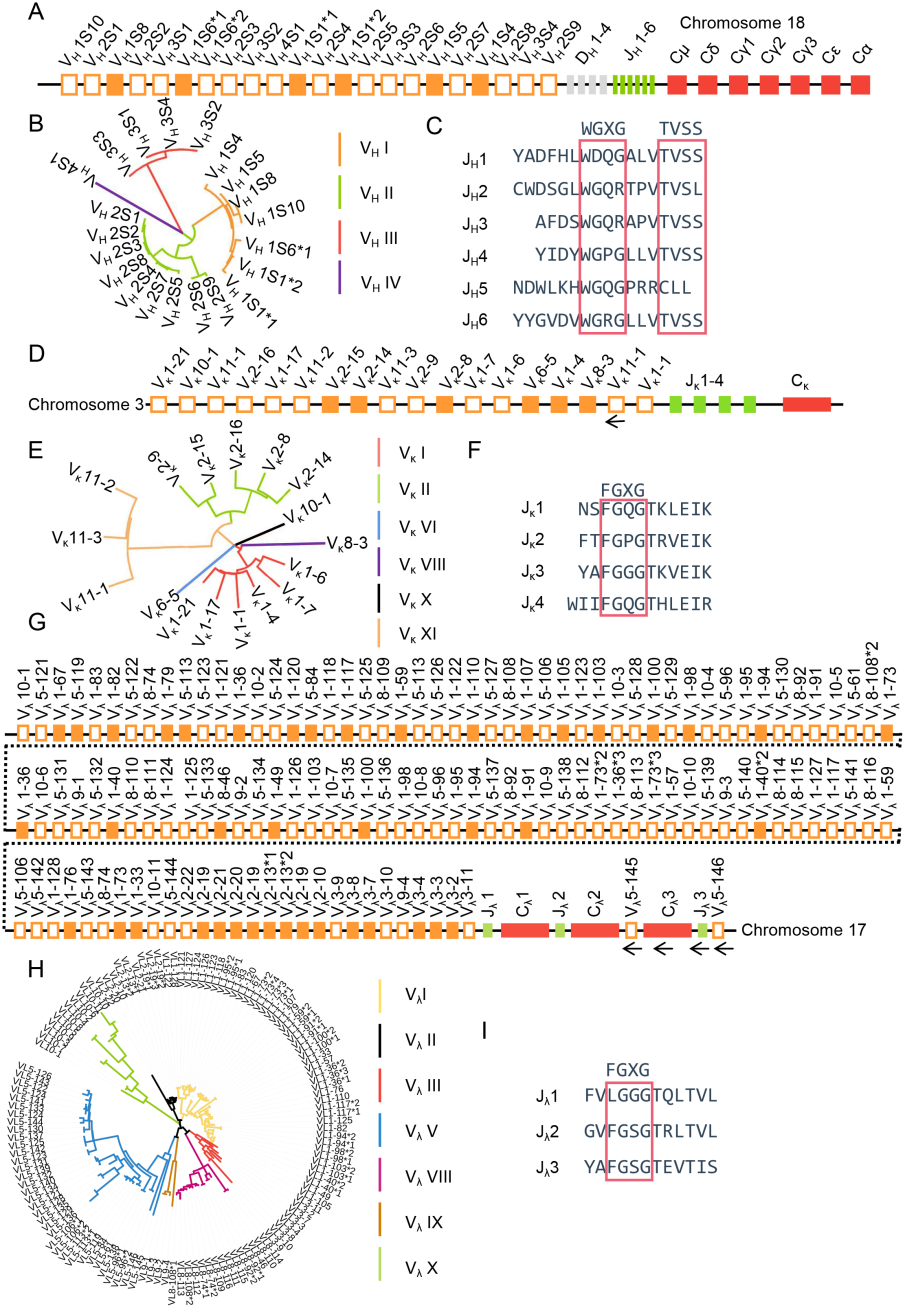
The schematic structure of the genomic organization of sheep IgH, Igκ and Igλ. **(A)** Schematic structure of the genomic organization of sheep IgH; **(B)** The phylogenetic tree of sheep VH; **(C)** The amino acid sequence of sheep JH; **(D)** Schematic structure of the genomic organization of sheep Igκ; **(E)** The phylogenetic tree of sheep Vκ; **(F)** The amino acid sequence of sheep Jκ; **(G:** Schematic structure of the genomic organization of sheep Igλ; **(H)** The phylogenetic tree of sheep Vλ; **(I)** The amino acid sequence of sheep Jλ.

Using all V, D, J, and C sequences from species in the IMGT database (https://www.imgt.org/) as reference templates, combined with FUZZNUC (http://embossgui.sourceforge.net/demo/fuzznuc.html) for RSS retrieval, we employed NCBI BLAST (https://blast.ncbi.nlm.nih.gov/Blast.cgi) to localize the immunoglobulin heavy and light chain loci in sheep. The IgH locus was identified on sheep chromosome 18 (GenBank: CM028721.1 NC_056071.1). Spanning 363 kb, it comprises 22 IgHV gene segments, 4 DH gene segments, 6 JH gene segments, and 6 constant region genes. The constant region genes include one μ gene, one δ gene, two γ genes, one ϵ gene, and one α gene (**Figure 1A**). The 22 IgHV segments clustered into four distinct subgroup, with all 6 functional VH genes belonging to subgroup I (**Figure 1B**). Among the 6 JH segments, only JH4 and JH6 exhibited the conserved amino acid motifs “WGXG” and “TVSS” (**Figure 1C**).

The Igκ locus was retrieved on sheep chromosome 3 (GenBank: CM028706.1, NC_056056.1) (**Figure 1D**). It spans 117 kb and includes 18 Vκ genes (of which 6 were functional, 3 were ORFs, and 9 were pseudogenes), 4 Jκ genes (all containing the conserved “FGXG” motif) (**Figure 1F**), and 1 Cκ gene. Classification was performed according to the IMGT annotation system (https://www.imgt.org/IMGTScientificChart/SequenceDescription/IMGTfunctionality.html). The Vκ genes clustered into six subgroups, with functional genes predominantly located in subgroup I and subgroup II consisting entirely of pseudogenes (**Figure 1E**).

The Igλ locus was found on sheep chromosome 17 (GenBank:CM028720.1, NC_056070.1), spanning 1412 kb. It contains 128 Vλ genes, of which 42 were functional, 9 were ORFs, and 77 were pseudogenes. Three Jλ-Cλ pairs are positioned downstream of the Vλ genes; however, the Vλ5-145 - Cλ3 - Jλ3 - Vλ5-146 segment was transcribed in the opposite orientation relative to the chromosomal transcription direction (**Figure 1G**). However, the FR1 region of Vλ5-145 is incomplete, and RSS was not retrieved for Vλ5-146. The 128 Vλ genes were classified into seven subgroups, with the majority of functional Vλ genes located within subgroup I, II and III (**Figure 1H**). Jλ2 and Jλ3 contains the conserved “FGXG” amino acid motif (**Figure 1I**). The V, D, J and C gene segment sequences are deposited in **Supplementary 1**.

### 3.2 Diversity analysis of V, D, J gene segment expression in sheep IgH

RNA was extracted from the spleens of three adult sheep. Specific IgH GSP primers were used to perform 5’RACE PCR to obtain recombinant fragments. Following sequence validation by Sanger sequencing, barcode labels were added to the 5’ RACE PCR products, which were then subjected to high-throughput sequencing. After preliminary screening of the sequencing data (fragment length and sequence integrity), the three samples yielded 4,244, 2,942, and 3,472 valid reads, respectively. These results demonstrate high coverage and high reliability, confirming the suitability of the data for subsequent analysis.

Analysis of sheep IgH expressed sequences was performed using IMGT, enabling the determination of VH, DH, and JH gene segment expression frequencies. The results revealed that IgHV1S1, IgHV1S5, and IgHV1S4 were expressed in all three samples, while IgHV1S8 exhibited no expression in Sample 1 and only minimal expression (0.1%) in Samples 2 and 3. Significant inter-sample variation was observed in IgHV expression profiles: Sample 1 showed frequencies of 58.13% (IgHV1S1), 12.63% (IgHV1S5), and 29.24% (IgHV1S4); Sample 2 displayed 67.26% (IgHV1S1), 19.74% (IgHV1S5), and 13% (IgHV1S4); and Sample 3 exhibited 40.53% (IgHV1S1), 15.6% (IgHV1S5), and 43.87% (IgHV1S4). Notably, IgHV1S4 demonstrated substantial variation in expression frequency across samples. All four DH genes were expressed with comparable frequencies among the three samples. IgHD2 predominated, exceeding 50% expression, significantly higher than the other three DH genes which ranged between 10%-20%. Regarding JH gene utilization, only JH4 and JH6 were incorporated into sheep IgH rearrangements. JH4 was the dominant joining segment, with expression frequencies of 88.3% in Sample 1, 91.1% in Sample 2, and 86.5% in Sample 3, while the remaining rearrangements exclusively employed JH6 (**Supplementary 2**).

### 3.3 Diversity analysis of V, D, J gene segment expression in sheep IgL

To investigate the expression characteristics of sheep Igκ recombination sequences, spleen RNA samples were extracted from three adult sheep. These samples were amplified using specific GSP primers via 5’RACE PCR. The obtained sequences were validated using Sanger sequencing, after which the 5’RACE PCR products were barcoded and subjected to high-throughput sequencing. Following preliminary screening, which included fragment length assessment and sequence integrity evaluation, the three samples yielded 5,301, 6,779, and 5,303 valid reads, respectively. Analysis of the sheep Igκ recombination sequences using the IMGT database successfully determined the expression frequencies of the Vκ and Jκ gene segments. The results revealed the utilization of five Vκ gene segments in recombination. Among these, Vκ1-4 exhibited the highest usage frequency across all three samples (54.5%, 75.4%, and 57.9%, respectively), followed by Vκ2-8 (43.3%, 11.7%, and 40.0%). Vκ2-14 usage showed significant variation between samples (1.5%, 12.8%, and 1.6%), while Vκ2-15 and Vκ8-3 were rarely expressed (below 1%). For the Jκ gene segments, expression was dominated by Jκ1 and Jκ3, with Jκ2 expression at approximately 0.2%. Jκ1 demonstrated the highest expression frequency in all three samples (62.1%, 62.7%, and 57.8%, respectively) (**Supplementary 2**).

Following high-throughput sequencing of the Igλ 5’RACE PCR products, the final numbers of valid reads obtained were 7,901, 6,862, and 6,041, respectively. Analysis of expression diversity using the IMGT database after filtering sequences revealed a rich repertoire of Vλ gene expression within the recombinant sequences, with a total of 26 distinct Vλ genes detected participating in Igλ V-J recombination. In Sample 1, Vλ3-8 and Vλ1-103 were expressed at rates of 21.7% and 11%, respectively. Sample 2 showed expression of Vλ1-36, Vλ2-10, and Vλ1-103 at rates of 15.3%, 14.7%, and 12.2%, respectively. For Sample 3, Vλ2-13, Vλ1-103, Vλ1-36, and Vλ2-10 were expressed at 15%, 14.2%, 12.2%, and 10%, respectively. Genes Vλ1-120, Vλ1-100, Vλ1-94, Vλ1-94, VλVλ2-21, Vλ3-7, and Vλ3-3 exhibited extremely low expression levels across all three samples, each consistently below 1%. The expression pattern of Jλ gene segments was relatively consistent, with only two types expressed: Jλ1 accounted for less than 0.05%, while the remaining sequences were Jλ2 (**Supplementary 2**).

### 3.4 Recombination and junction diversity analysis in sheep IgH

**Figure 2:**
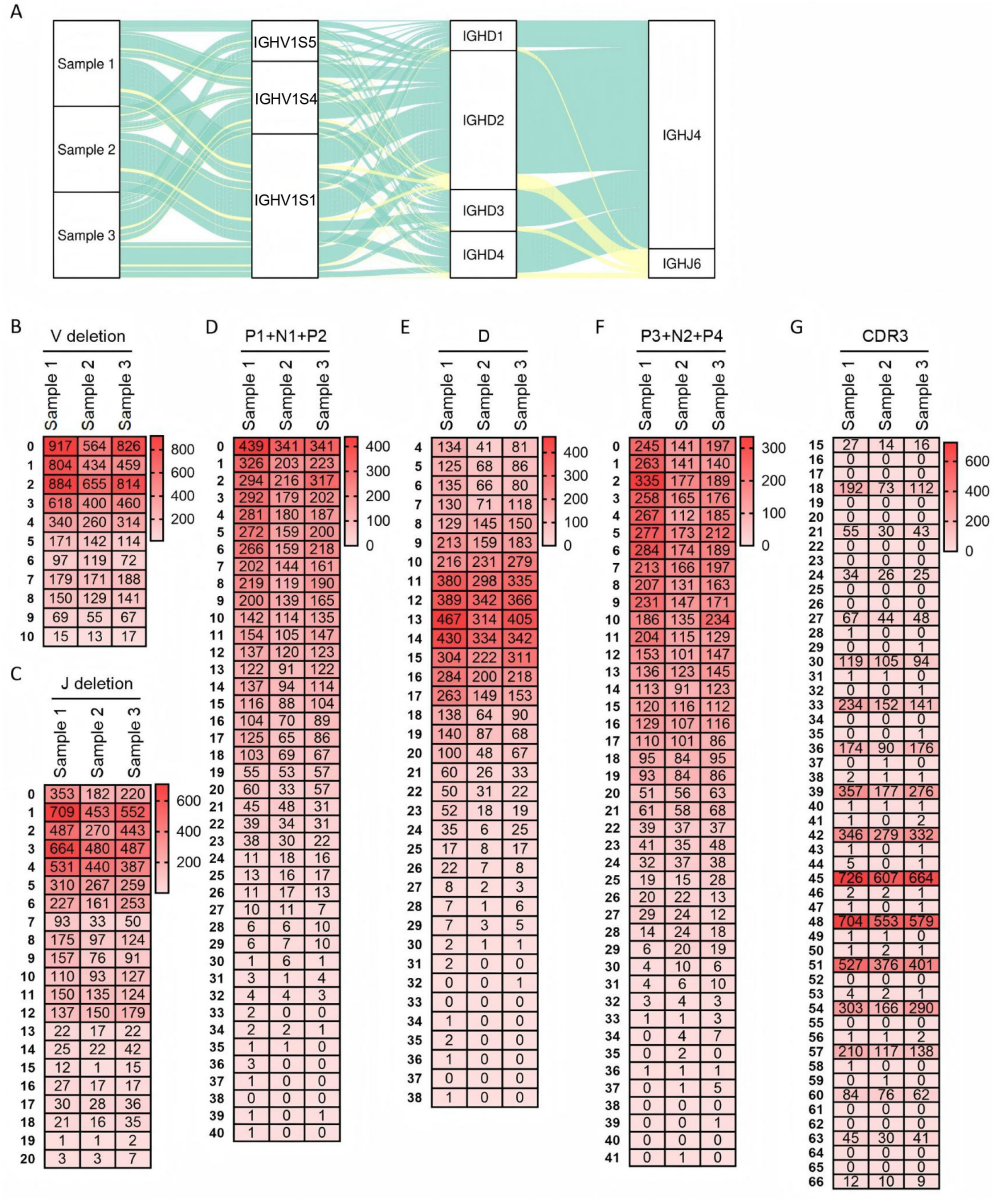
Recombination and junctional diversity of IgH in sheep. **(A)** The sankey diagram of VDJ recombination in sheep IgH; **(B–G)** The length distribution of 3’ V-deletion, P1+ N+P2 nucleotide, D fragment, P3+ N2+P4 nucleotide, 5’ J-deletion and CDR3 in sheep IgH.

**Figure 2A** shows the VDJ recombination diversity of three samples using a pie chart. Sequencing results revealed a total of 25 recombination types in Sample 1. Among these, VH1S1-DH2-JH4 exhibited the highest expression frequency (32.8%), followed by VH1S4 - DH2 - JH4 (14.1%). None of the other recombination types exceeded 10%. Sample 2 contained 26 recombination types, with VH1S1 - DH2 - JH4 being the most frequent (34%), followed by VH1S1 - DH4 - JH4 (11.1%). The remaining recombination types each accounted for less than 10%. Sample 3 presented 27 recombination types, with recombination types exceeding 10% expression being VH1S1 - DH2 - JH4 (20.3%) and VH1S4 - DH2 - JH4 (19.2%). The most frequent recombination type was consistent across all three samples. Specifically, recombination types with expression frequencies below 1% were identified in 8 cases in Sample 1, 12 cases in Sample 2, and 10 cases in Sample 3. The expression frequency for each recombination type, including those mentioned above, is presented in **Supplementary 3**.

As the core component of the immunoglobulin variable region, the CDR3 region critically determines antibody specificity and affinity. The length and amino acid sequence diversity of this region significantly impact immunoglobulin function. In the sheep IgH CDR3 domain, length contribution can be dissected into five major factors: random deletion at the VH gene end (3’V-deletion), diversity in P1 + N1 + P2 nucleotides length, diversity in CDR3H length, diversity in P3 + N2 + P4 nucleotides length, and random deletion at the JH gene end (5’ J-deletion). Analysis of sheep 3’ V-deletion revealed a predominant deletion of 0~4 bp (**Figure 2B**), while 5’ J-deletion was mainly concentrated within 0~12 bp (**Figure 2C**). The combined length of P1 + N1 + P2 nucleotides was predominantly 0~9 bp (**Figure 2D**), and the length of P3 + N2 + P4 nucleotides was primarily 0~11 bp (**Figure 2F**). Statistical analysis of DH gene segment length in the ovine samples showed that DH lengths were predominantly distributed within the 11~17 bp range, followed by the 4~10 bp range. The longest observed DH segment reached 38 bp (**Figure 2E**).

Further analysis of CDR3H length distribution demonstrated that CDR3H lengths in all three samples were primarily concentrated at 45 bp, 48 bp, and 51 bp, exhibiting a pattern of incrementation by 3 bp. The 3bp variation pattern of CDR3 may be related to productive recombination. The maximum observed CDR3H length was 66 bp (**Figure 2G**).

### 3.5 Recombination and junction diversity analysis in sheep IgL

**Figure 3:**
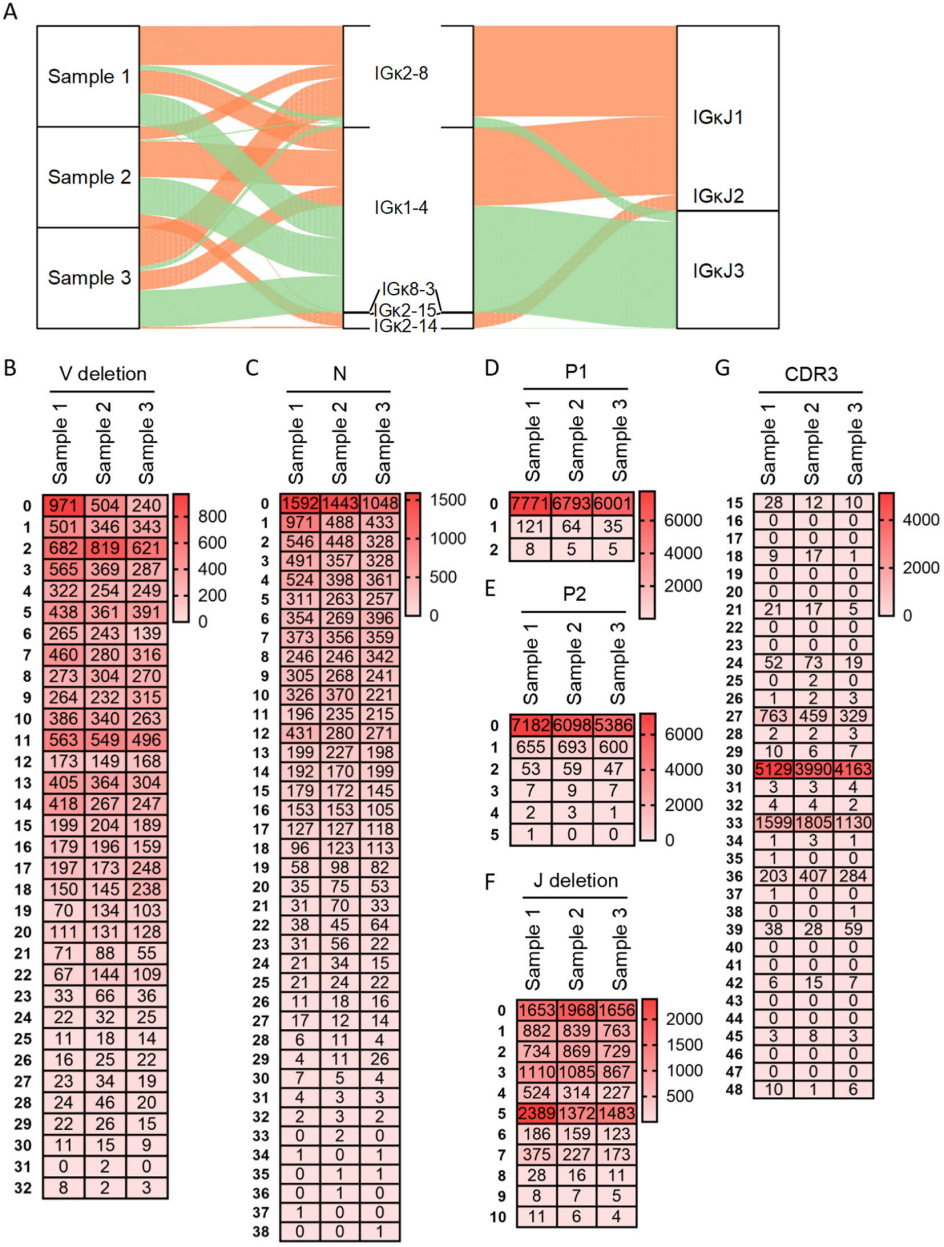
Recombination and junctional diversity of IgL(κ) in sheep. **(A)** The sankey diagram of VJ recombination in sheep IgL(κ); **(B–G)** The length distribution of 3’ V-deletion, N nucleotide, P1 nucleotide, P2 nucleotide, 5’ J-deletion and CDR3 in sheep IgL(κ).

**Figure 3A** depicted the diversity of Igκ Vκ-Jκ recombination. The three samples exhibited 12, 12, and 13 distinct recombination types, respectively. In Sample 1, the predominant types were Vκ2-8-Jκ1 (39%), Vκ1-4-Jκ3 (32.9%), and Vκ1-4-Jκ1 (21.3%); the remaining 9 types collectively accounted for 6.8%. Sample 2 showed high expression frequencies (>10%) for Vκ1-4-Jκ1 (40.2%), Vκ1-4-Jκ3 (35.1%), Vκ2-15-Jκ1 (12.3%), and Vκ2-8-Jκ1 (10%); the other 8 types represented only 2.3%. In Sample 3, Vκ1-4-Jκ3 (38.6%), Vκ2-8-Jκ1 (36.8%), and Vκ1-4-Jκ1 (19.2%) were the most abundant types; the remaining 10 types constituted 5.4%. The recombination types Vκ2-8-Jκ1, Vκ1-4-Jκ1, and Vκ1-4-Jκ3 were consistently highly expressed across all samples. Analysis of Igλ junctional diversity revealed no discernible pattern in 3’ V-deletion segment lengths. The longest length could reach 32 bp, concentrated in the range of 0 bp to 14 bp and the 3’ V-deletion lengths are mainly 11 bp, 2 bp and 0bp (**Figure 3B**). Rearranged Igλ sequences lacking N nucleotides were predominant, followed by those with 1 bp N additions (**Figure 3C**). Sequences without P nucleotides accounted for 98.4%, 99%, and 99.3% in the three samples respectively, with P nucleotide lengths never exceeding 2 bp. Sequences lacking P2 nucleotides constituted 90.9%, 88.9%, and 89.2% respectively, while P2 nucleotide length did not exceed 5 bp (**Figures 3D, E**). Rearranged sequences exhibiting 5 bp deletions 5’ J-deletion were most abundant, followed by 0 bp and 3 bp deletions; the maximum 5’ J-deletion length observed was 10 bp (**Figure 3F**). The CDR3κ length distribution demonstrated strong regularity comparable to CDR3κ, with predominant lengths at 30 bp, 33 bp, and 27 bp. The maximum CDR3κ length observed was 48 bp (**Figure 3G**).

**Figure 4:**
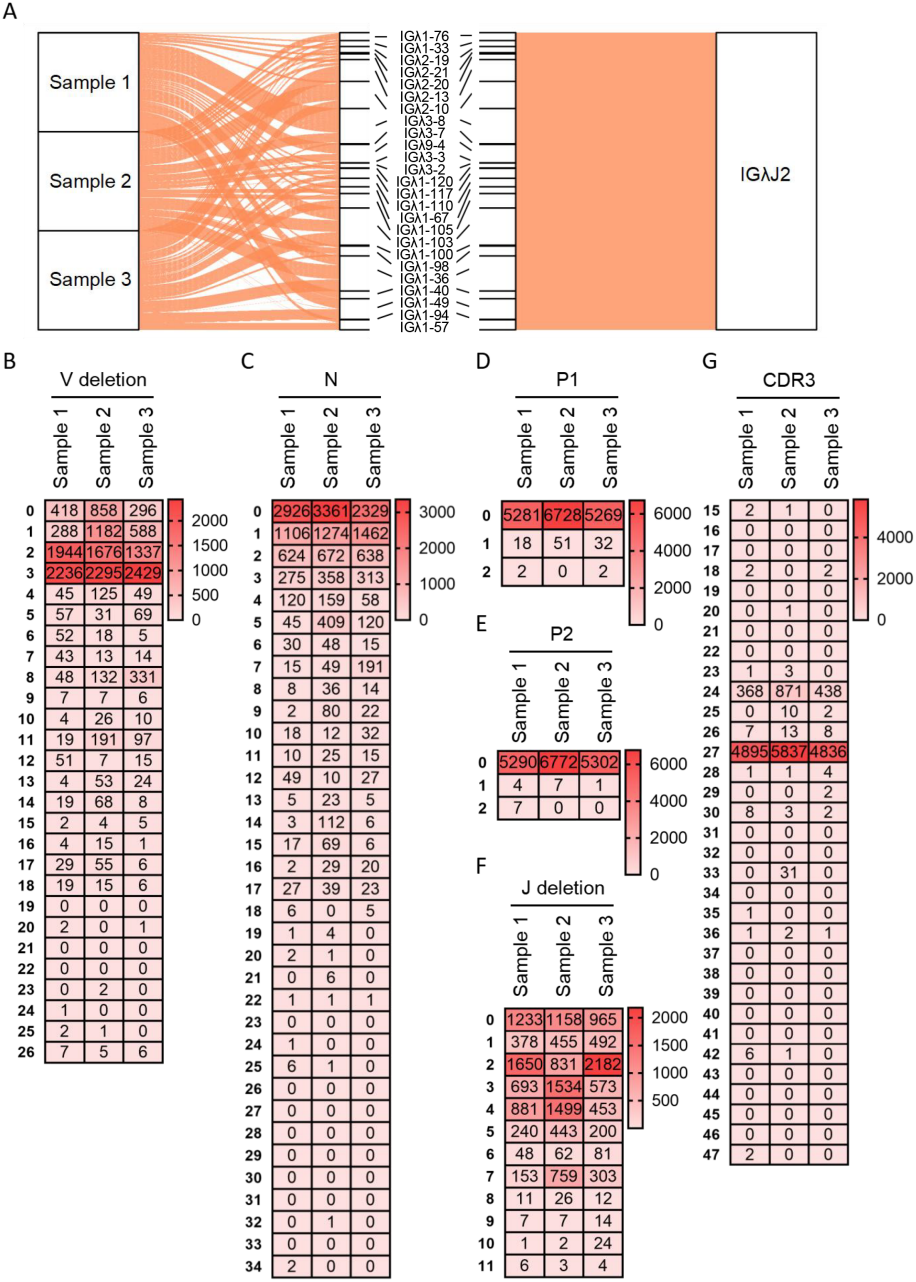
Recombination and junctional diversity of IgL(λ) in sheep **(A)** The sankey diagram of VJ recombination in sheep IgL(λ); **(B–G)** The length distribution of 3’ V-deletion, N nucleotide, P1 nucleotide, P2 nucleotide, 5’ J-deletion and CDR3 in sheep IgL(λ).

Diversity analysis was similarly performed for Vλ-Jλ rearrangements of the Igλ chain. The Vλ-Jλ rearrangement repertoire exhibited the greatest richness, attributable to the abundance of germline Vλ genes, with Jλ2 expression frequency reaching 99.5%. Sample 1 contained 28 rearrangement types, among which Vλ3-8 - Jλ2 demonstrated the highest recombination frequency (21.7%), followed by Vλ1-103 - Jλ2 (11.1%) and Vλ1-149 - Jλ2 (9.8%). Sample 2 contained 26 rearrangement types, with three types exceeding 10% frequency: Vλ2-10 - Jλ2 (15.4%), Vλ2-10 - Jλ2 (14.7%), and Vλ1-103 - Jλ2 (12.2%). Sample 3 contained 26 rearrangement types, where Vλ2-13 -J λ2 was most frequent (15%), followed by Vλ1-103 - Jλ2 (14.2%), Vλ1-36 - Jλ2 (12.2%) and Vλ2-10 - Jλ2 (10%). Notably, the Vλ1-103 - Jλ2 rearrangement maintained relatively high frequency across all three samples (**Figure 4A**). Further junctional diversity analysis revealed that 3’ V-deletion lengths were predominantly 3 bp and 2 bp (**Figure 4B**), while N nucleotide lengths mainly ranged from 0 to 2 bp, with a maximum observed length of 34 bp (**Figure 4C**). Both P1 and P2 nucleotide lengths were ≤2 bp; sequences with P1 lengths of 1 bp or 2 bp represented ≤1%, and those with P2 lengths of 1 bp or 2 bp constituted ≤0.1% (**Figures 4D, E**). 5’ J-deletion lengths were primarily 0-4 bp, extending up to a maximum of 11 bp (**Figure 4F**). CDR3λ length distribution followed consistent patterns in all three samples: the predominant length was 27 bp (frequencies: 92.3%, 86.1%, and 91.2% in Samples 1, 2, and 3, respectively), followed by 24 bp (frequencies: 6.9%, 12.8%, and 8.3%). However, the maximum observed CDR3λ lengths were notably shorter at 47 bp, 42 bp, and 36 bp in the respective samples (**Figure 4G**).

### 3.6 The SHM of sheep IgH and IgL

**Figure 5:**
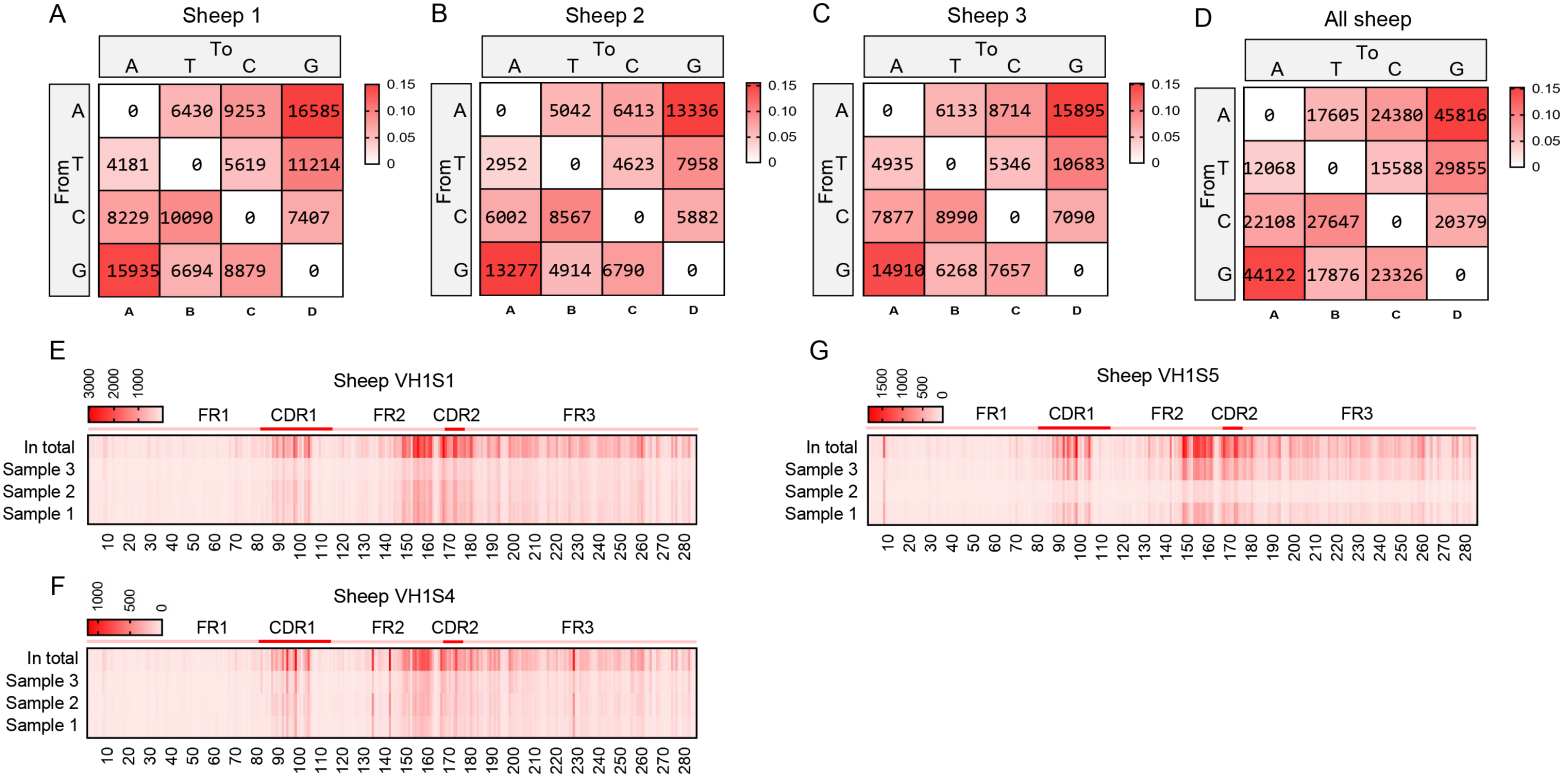
The somatic hypermutation (SHM) of sheep IgH. **(A–D)** The base mutation types of SHM in sheep IgH; **(E–G)** The distribution of SHM in sheep IgH. Notes: the color-shading from Figure **(A–D)** represents the frequency of SHM; the color-shading from Figure **(E–G)** represents the counts of SHM.

By aligning each reads sequence with its corresponding germline VH gene, we determined the mutation type at each position in the VH segment. In the grid plots, the background shading intensity represents the proportion of each base substitution type relative to the total number of mutations, while the data indicates the frequency of that specific substitution type per individual. The SHM variation preferences were highly consistent across the three samples: the highest mutation frequency was consistently A→G, followed by G→A, and the lowest was consistently T→A (**Figures 5A–D**). Based on the mutation frequency of each base, **Figures 5E–G** were generated. These figures reveal that mutations primarily occur within the CDR regions. However, a region of high-frequency mutation persists outside the CDRs (specifically in the latter part of FR2), likely due to the presence of mutation hotspots, indicating that SHM is not confined solely to CDR regions, and other regions also exhibit high levels of mutation frequency.

**Figure 6:**
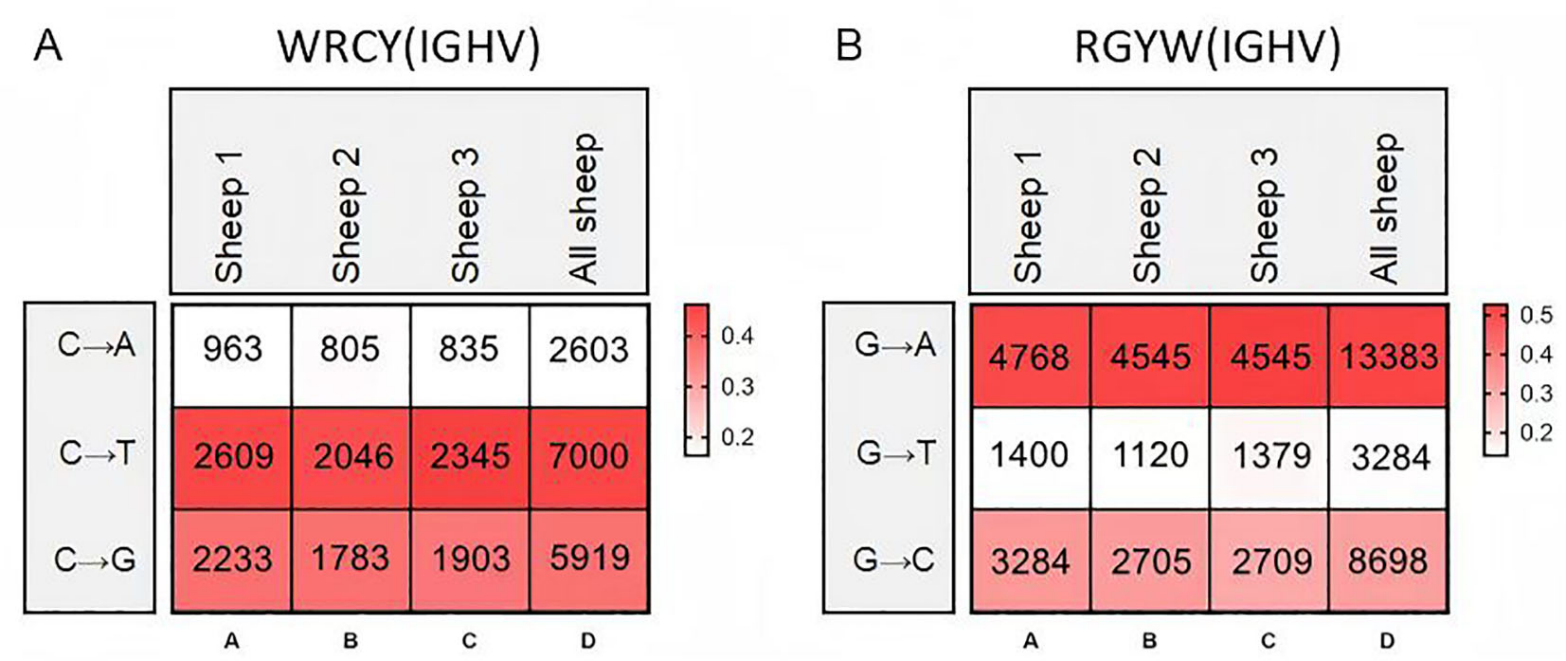
Hotspot mutation frequency of SHM in sheep IgH. **(A)** The sheep IgH SHM in “WRCY” locus; **(B)** The sheep IgH SHM in “RGYW” locus.

Therefore, we further analyzed the mutation frequency of C/G within the AID hotspot motifs WRCY/RGYW, specifically the proportion of C/G mutations within these hotspots relative to the total mutated bases. The numbers within the boxes represent mutation counts, and the background color represents the frequency of each mutation type within that individual. The figures demonstrate that: (1) Within “WRCY”, the C→T substitution has the highest frequency, while C→A has the lowest frequency (**Figure 6A**); (2) Within “RGYW”, G→A has the highest frequency, and G→T has the lowest frequency (**Figure 6B**); (3) Therefore, IgH hotspot mutations exhibit distinct type preferences.

**Figure 7:**
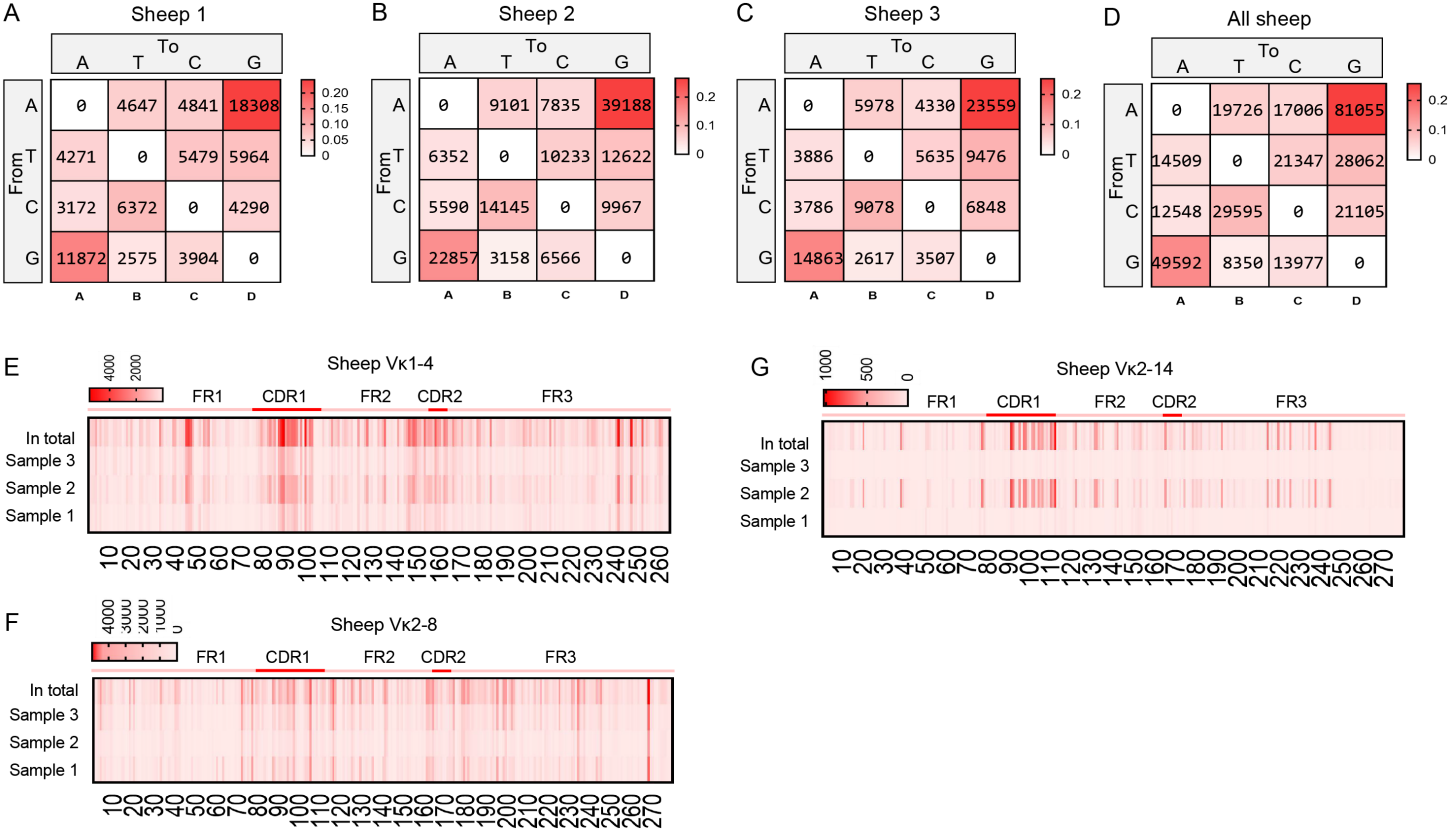
The somatic hypermutation (SHM) of sheep IgL(κ). **(A–D)** The base mutation types of SHM in sheep IgL(κ); **(E–G)** The distribution of SHM in sheep IgL(κ). The color-shading from Figure **(A–D)** represents the frequency of SHM; the color-shading from Figure **(E–G)** represents the counts of SHM.

**Figure 8:**
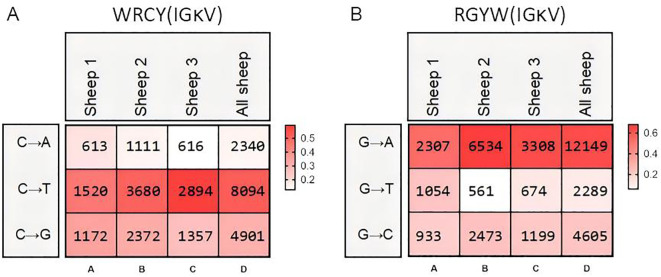
Hotspot mutation frequency of SHM in sheep IgL(κ). **(A)** The sheep IgL(κ) SHM in “WRCY” locus; **(B)** The sheep IgL(κ) SHM in “RGYW” locus.

Mutation bias and mutation frequency in Igκ SHM were analyzed using the same methodology. Similar to IgH, the A→G mutation frequency was the highest, accounting for 25.6%, followed by G→A (15.7%). However, the mutation frequency of G→T was the lowest at only 2.6%, which differed from the pattern observed in IgH (**Figures 7A–D**). Statistical analysis of mutation frequencies across all sites revealed that, besides the CDR regions, a few sites within the FR regions exhibited high-frequency mutations (**Figures 7E–G**). Further analysis of the mutation frequency of C/G within the AID hotspot motifs WRCY/RGYW showed that: In “WRCY”, C→T mutations occurred most frequently, while C→A mutations were the least frequent (**Figure 8A**); In “RGYW”, G→A mutations were the most frequent, while G→T mutations were the least frequent (**Figure 8B**). Igκ hotspot mutations displayed a distinct bias.

**Figure 9:**
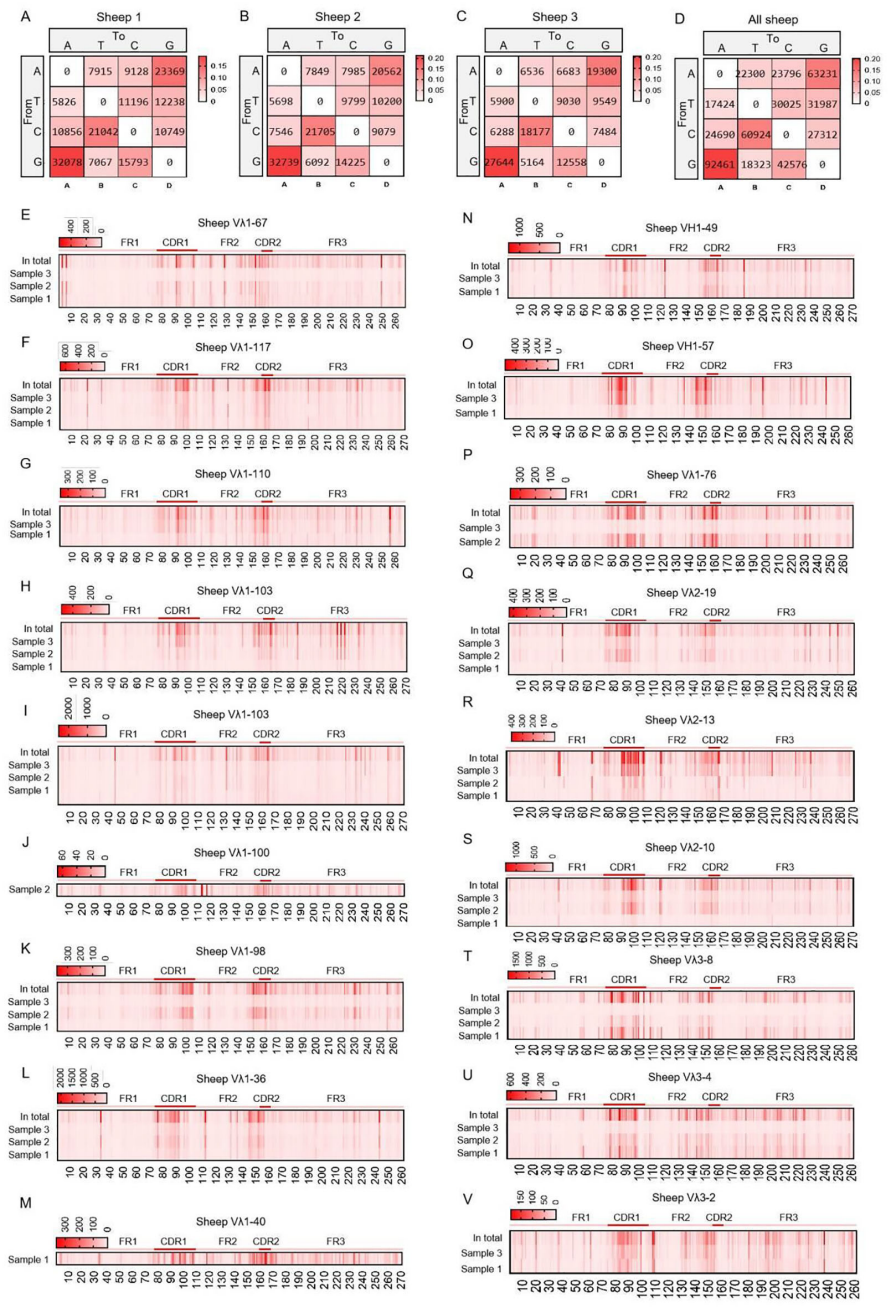
The somatic hypermutation (SHM) of sheep IgL(λ). **(A–D)** The base mutation types of SHM in sheep IgL(λ); **(E–V)** The distribution of SHM in sheep IgL(λ). Notes: the color-shading from Figure **(A–D)** represents the frequency of SHM; the color-shading from Figure **(E–V)** represents the counts of SHM.

**Figure 10:**
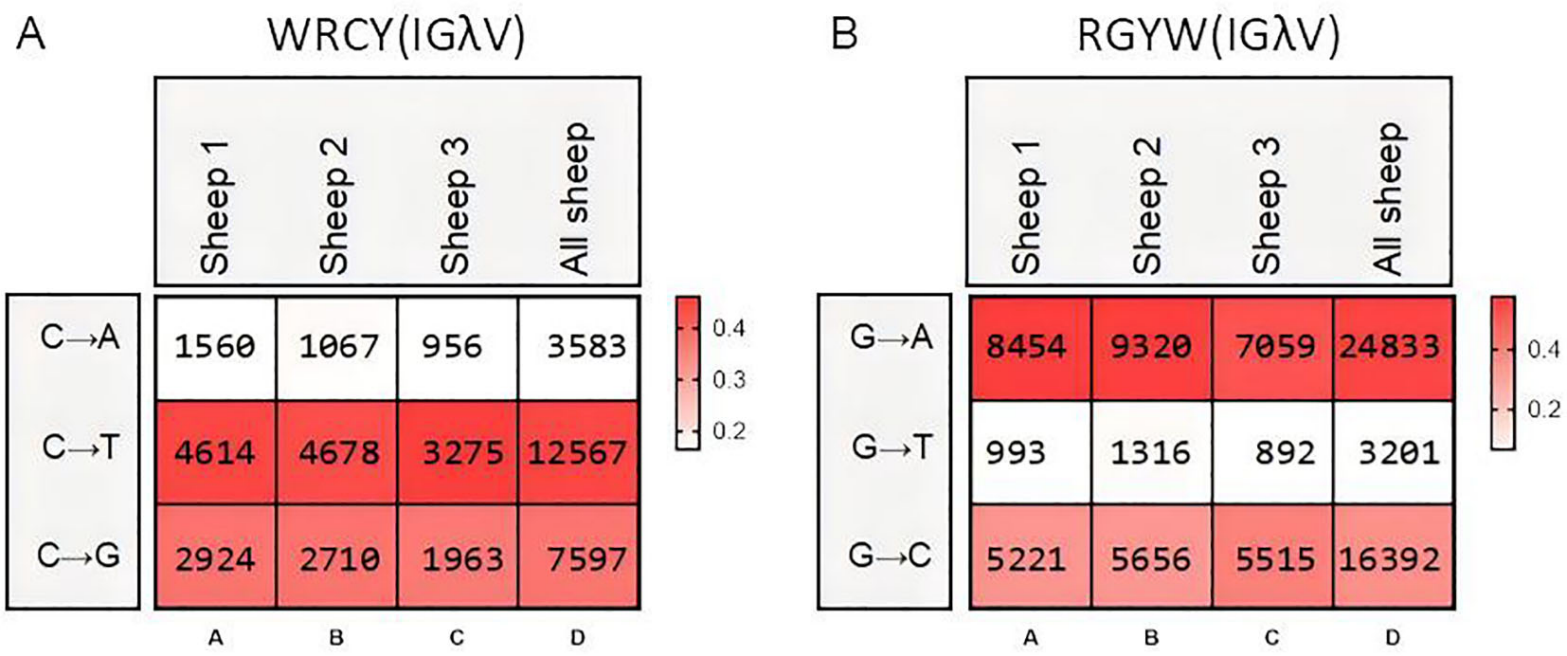
Hotspot mutation frequency of SHM in sheep IgL(λ). **(A)** The sheep IgL(λ) SHM in “WRCY” locus; **(B)** The sheep IgL(λ) SHM in “RGYW” locus.

The SHM in Igλ exhibited changes, with G→A substitutions occurring at the highest frequency, followed by A→G. The frequency of C→T mutations closely followed that of A→G, while T→A mutations showed the lowest frequency (**Figures 9A–D**). Given the involvement of numerous germline Vλ genes in recombination, the site-specific mutation frequencies were divided into 18 groups. Apart from the CDR regions, a few groups exhibited high-frequency mutation hotspots within the FR2 region (**Figures 9E–V**). Further analysis of the mutation frequency of C/G within the AID hotspot motifs WRCY/RGYW revealed that the hotspot mutational preference was consistent with that observed in IgH and Igκ. Specifically, within the WRCY motif, C→T mutations were the most frequent, while C→A mutations were the least frequent (**Figure 10A**). Similarly, within the RGYW motif, G→A mutations occurred at the highest frequency, and G→T mutations showed the lowest frequency (**Figure 10B**).

## 4 Discussion

In the analysis of the locus structure of sheep immunoglobulin heavy chain, the types and quantities of ultra-long DH and μ genes in the germline were mainly focused on. Regrettably, by locating RSS, only four DH gene fragments were identified in the sheep genome, indicating that the number of DH in the sheep germline was significantly lower than that in cattle and yak. Moreover, no ultra-long DH gene analogous to those found in cattle, yak, swamp buffalo, and river buffalo appears to exist in the sheep genome. The longest DH gene fragment identified in sheep is only 42 bp, while the longest germline DH segments in cattle, yaks, and swamp buffalo measure 149 bp, 193 bp, and 119 bp, respectively (27–30). The characteristics containing (YG)_n_ repetitive amino acid sequences were found in the DH2 (SYYSGYGYAYGY) and DH4 (SYYSDYGY) sequences of sheep, which were consistent with those of DH4 (SYSGYGYGYSYGY) and DH6 (SCYSGYGYGCGYGYGYDY) in domestic cattle and DH28 (SYSGYGYGGYGCYGYGYGY) and DH34 (SCYSGYGYGYGCGYGYGYDY) in yak (27, 28). The analysis of VDJ recombination preferences across three species revealed distinct preferential selection patterns in DH genes during the recombination process. In sheep, the DH2 exhibited the highest selection frequency, whereas cattle predominantly selected DH4 and DH6, yak predominantly selected DH28 and DH34. These findings suggest that Bovidae members exhibit an evolutionary tendency to preferentially select DH fragments containing (YG)n amino acid during VDJ recombination. The JH gene cluster is relatively conserved among reported bovid species. The JH clusters of sheep (Ovis aries), goat (Capra hircus), water buffalo (Bubalus bubalis), and the two JH clusters in cattle (Bos taurus) exhibit conservation in terms of JH gene number, sequence similarity, and arrangement order (27–31). All four species possess six JH genes within their JH clusters. Among these, JH1, JH2, JH3, and JH5 are pseudogenes, while JH4 and JH6 are functional genes. The primary expression is from JH4 (JH10 in cattle), with low-level expression of JH6 (JH12 in cattle). Notably, cattle possess two JH gene clusters. The JH1–JH6 cluster and the JH7–JH12 cluster display extremely high sequence similarity and identical arrangement order. Although only three JH genes, all pseudogenes, were identified in the yak (Bos grunniens) genome, reads obtained from 5’RACE data indicate that the primarily expressed JH gene in yak shares high identity with bovine JH4 (JH10). This suggests that the yak JH gene cluster may be similar to those of other bovids. Water buffalo harbors eight JH genes. JH1-1 to JH1-6 constitute the first JH cluster, while JH2-1 and JH2-2 potentially represent a second JH cluster. The predominantly expressed JH gene in water buffalo shows high identity with the bovid JH4 gene.

Comparative genomic analysis revealed that sheep, similar to yak, possess only a single μ gene (21, 28). Sequence alignment demonstrated that the ovine μ gene shares 94.5% similarity with bovine μ2 and 93.8% with bovine μ1, whereas the yak μ gene exhibits 98.7% sequence identity with bovine μ2. Notably, previous reported differential VDJ recombination patterns in cattle, showing preferential utilization of μ1 gene segments paired with JH6 elements and μ2 gene segments associated with JH10 during immunoglobulin rearrangement (28). These phylogenetic and functional observations collectively suggest that sheep, analogous to yak, may have undergone evolutionary loss of the μ1 gene through genomic deletion or pseudogenization events. Similar to yaks, sheep were found to possess only one μ gene, lacking the μ2 gene present in domestic cattle. A similarity analysis of the sheep μ gene with bovine μ genes revealed 94.5% similarity with the cattle μ2 gene and 93.8% similarity with the cattle μ1 gene. In contrast, the yak μ gene shows a significantly higher similarity of 98.7% with the domestic cattle μ2 gene. According to the research (28), domestic cattle tend to utilize the μ1 gene with JH6 and the μ2 gene with JH10 during VDJ recombination. These findings collectively suggest that sheep, similar to yaks, may have lost the μ1 gene in their evolutionary history (21, 28).

Although 25 VDJ recombinant types were identified in sheep, the three expressed VH genes shared 94% sequence similarity and clustered within the same gene subgroup. The expression of JH genes and DH genes also had strong expression preference, consistent with patterns observed in other ruminant species including goat, cattle, and yak. These findings suggested that IgH VDJ recombination contributes minimally to antibody repertoire diversity in these species. Conservation patterns were similarly observed in the Jκ gene locus, with both bovine and yak genomes containing six Jκ gene segments demonstrating high sequence homology. Comparative analysis revealed that the sheep genome lacked the Jκ1 segment, retaining only five functional Jκ gene segments. Notably, expression profiling of immunoglobulin recombination events in sheep showed differential utilization patterns, with Jκ3 representing the most highly expressed segment, followed by Jκ2 based on somatic recombination frequency analysis.Our previous investigations demonstrated that approximately 80%-90% of immunoglobulin recombination in domestic cattle and yak utilized the Jκ2 gene segment, while 10%-20% of recombinant sequences contained Jκ4. For sheep Igκ, five Vκ gene segments and three Jκ gene segments were involved in recombination, with a total of 12 recombination types, but Vκ2-8 - Jκ1 (38.9%), Vκ1-4 - Jκ3 (32.9%) and Vκ1-4 - Jκ1 (21.3%) accounted for 93.1% of the analyzed reads. The recombination process of yak Igκ involved six Vκ gene segments and four Jκ gene segments (27). A total of 14 distinct recombination types were identified, among which three predominant modes—Vκ1-1 - Jκ2, Vκ1-1 - Jκ4, and Vκ1-1 - Jκ2—were frequently observed. With genome reassembly and optimization for completeness, more germline Vλ gene segments were discovered. Previous studies had only mapped 32 Vλ gene segments, whereas this study mapped 128 germline Vλ gene segments. This also represents one of the main differences in Igλ between sheep and other bovids: yaks possess only 42 germline Vλ gene segments, swamp buffalo only 29, and goats only 35 (27, 30, 32, 33). There were significant differences in the Igλ locus between yak and sheep. Sheep possess 128 germline Vλ genes, while yak had only 45. Notably, the Jλ and Cλ genes exhibit distinct organizational patterns: sheep display a simple alternating arrangement of 3 Jλ and 3 Cλ gene segments, whereas yaks demonstrate a more complex organization with 9 Jλ and 7 Cλ gene segments arranged in the following sequential pattern: Jλ1-Jλ2-Cλ1-Cλ2-Jλ3-Jλ4-Jλ5-Cλ3-Jλ6-Cλ4-Jλ7-Cλ5-Jλ8-Cλ6-Jλ9-Cλ7. Existing studies have revealed that the Igλ loci in most mammals exhibit a conserved (Jλ-Cλ)_n_ arrangement pattern. Specifically, the equine Igλ locus demonstrates a Vλ_n_-(Jλ-Cλ)_7_-Vλ_n_ configuration, domestic cattle possess a Vλ_n_-(Jλ-Cλ)_4_ structure, pig display a Vλn-(Cλ-Jλ)_3_-λ4 organization, and caprine species feature a Vλ_n_-(Jλ-Cλ)_3_ arrangement (33–36). Unfortunately, the Vλ5-145 – Cλ3 – Jλ3 – Vλ5-146 loci discovered in the sheep genome with opposite transcriptional orientation do not participate in rearrangement due to the incomplete FR1 of Vλ5-145 and the absence of RSS in Vλ5-146. Therefore, our study was unable to demonstrate whether Vλ-Jλ-Cλ loci with opposite transcriptional orientation can undergo rearrangement. Notably, the sheep Igλ locus conforms to this established genomic organizational principle. IMGT analysis of sheep Igλ rearranged sequences revealed the utilization of 26 Vλ genes during VJ recombination in sheep, compared with 10 Vλ genes employed by yak. Although the diversity of Vλ gene segments participating in recombination appeared considerable, these gene segments were ultimately grouped into four distinct families, exhibiting extremely high sequence similarity among subgroup members. During recombination, Jλ1 was utilized in less than 0.1% of sequences, while Jλ2 constituted the remainder. All yak sequences exclusively employed Jλ3 genes, whereas domestic cattle demonstrated recombination involving both Jλ2 and Jλ3. Sequence similarities exceeding 80% are observed in the Jλ genes utilized in recombination across these three bovid species. Although interspecies variations exist in germline Jλ genes, the recombination preference remains consistent, with all species selecting Jλ genes from this conserved subgroup for VJ recombination. Consequently, the diversity of Igλ generated through VJ recombination proves equally limited as observed in IgH and Igκ chains.

Is junctional diversity during recombination a key factor in the generation of immunoglobulin diversity in sheep? We conducted separate analyses of the junctional regions of IgH, Igκ, and Igλ through five parameters: DH segment length, CDR3 length, N/P nucleotide length, and random deletion lengths at V/J junctions.

In sheep, the longest CDR3H was 66 bp, with a mean length of 44 ± 9.7 bp. In contrast, the longest CDR3H observed in yaks is 129 bp, while in domestic cattle, it reaches 195 bp. The CDR3H lengths in sheep are predominantly concentrated at 45 bp, 48 bp, and 51 bp. Domestic cattle exhibit a predominant clustering of CDR3H lengths at 63 bp, 66 bp, and 69 bp, whereas yaks show the highest frequencies at 57 bp and 60 bp. The sheep genome did not reveal ultra-long DH gene segment, with the longest DH fragment utilized during recombination being 38 bp in length. Consequently, no exceptionally long CDR3H regions analogous to those observed in domestic cattle, yaks and buffalo were identified in the sheep immunoglobulin. The DH gene segments in human and mouse contributed 14.3 ± 5.5 bp and 10.8 ± 4.7 bp, respectively, to the CDR3H region. In cattle, the DH-Cμ1 and DH-Cμ2 segments contributed 27.5 ± 8.5 bp and 43.1 ± 25.3 bp to CDR3H length, while yak DH gene segments contributed 39.9 ± 13.7 bp (37). The average DH-derived CDR3H length in sheep was 12.9 ± 4.3 bp, which was significantly shorter than those observed in both cattle and yak, and only marginally exceeds that of mouse. Previous studies have identified ultra-long CDR3H as a unique diversity-enhancing immunoglobulin feature exclusive to domestic cattle and yak. The primary focus of this investigation was to determine whether this structural characteristic exists in other members of the Bovidae family. While ultra-long CDR3H was not observed in sheep, our analysis revealed that the distinctive (Yx)_n_ amino acid motif encoded in cattle and yak ultra-long CDR3H sequences was unexpectedly present in sheep CDR3H regions. This finding contradicts our initial hypothesis that the (Yx)n motif was specifically associated with ultra-long CDR3H architecture. Instead, our data suggest that Bovidae species may exhibit an evolutionary preference for utilizing DH gene segments encoding (Yx)n amino acid motifs, independent of CDR3H length characteristics.

SHM occurs in activated B cells, further increasing immunoglobulin diversity beyond the initial repertoire generated by the organism, and is also an indispensable step for antibody affinity maturation (13, 38). The SHM pattern in the sheep IgH exhibits a distinct nucleotide substitution bias, with A→G and G→A transition mutations occurring at the highest frequency, followed by T→G transversions. Among these, A→G mutations are most likely generated during the second phase of SHM: the error-prone Polη tends to A→G in WA motifs (where W denotes A/T) during the repair of DNA damage. Conversely, G→A and C→T mutations likely occur during the first phase of SHM: C→U mutations produced by AID deamination become fixed in subsequent DNA replication. These mutational signatures are evolutionarily conserved across multiple species, including domestic cattle, yak, mouse, and zebrafish, indicating that the SHM mechanism has been retained throughout vertebrate evolution (28, 39). Notably, AID preferentially targets hotspot motifs (RGYW/WRCY) for mutagenesis. AID initiates the process through cytidine deamination, creating U:G mismatches. These mismatches are then channeled into distinct repair pathways via different molecular factors. This mechanistic preference leads to elevated mutation frequencies of C and G within these hotspot motifs. Consequently, significant mutation clustering is observed in FRs as well as the classical CDRs, due to the presence of these hotspots (40). Certain limitations also exist in the SHM analysis process: (1) SHM variant types may exhibit certain variations with genomic updates and the assembly of different sheep genome assemblies. (2) **Figure 9E** displays not only variant types but also variant frequencies. Although there may be unavoidable errors in the variant types at this locus, it can still demonstrate the richness of SHM at this site.

In summary, different species appear to have evolved distinct Ig gene rearrangement sequences and strategies for selecting functional immunoglobulin repertoires (41). Comparative analysis of immunoglobulin diversity generation mechanisms across species reveals that humans and mouse display extensive V(D)J recombination diversity, enabling them to generate a broad repertoire of immunoglobulin types through recombination to meet diverse antigenic challenges (42). In species with limited V(D)J recombination diversity, such as rabbits, this limitation is compensated for by GCV occurring in specific B-cell lineages combined with high levels of SHM, ultimately producing antibody repertoires with greater diversity than those observed in humans or mice. Structurally, rabbits rely more heavily on the IgL for antigen specificity, utilizing extended CDR3L loops and interdomain disulfide bonds (43). In camelids, B cells produce both conventional IgH/IgL paired antibodies and unique HCAbs. These HCAbs contain distinctive structural features that further enrich the camelid antibody repertoire. Previous studies found that domestic cattle, yak, swamp buffalo, and river buffalo utilize ultra-long CDR3H to enhance immunoglobulin diversity. Sheep appear to enrich their antibody repertoire through recombination diversity and junctional diversity of Igλ. Notably, while the V and J gene segments show strong conservation in bovid, the unique ultra-long CDR3H mechanism is absent in sheep, indicating that the ultra-long CDR3H is not a conserved diversity generation strategy among bovid. The evolutionary drivers behind the specific emergence of this mechanism in domestic cattle and yak require further investigation.

## Data Availability

The datasets presented in this study can be found in online repositories. The names of the repository/repositories and accession number(s) can be found in the article/**Supplementary Material**.

## Supplementary material

Supplementary 1 The complete nucleotide sequences of V(D)J genes.

Supplementary 2 The Expression of V(D)J in Sheep IgH and IgL.

Supplementary 3 VDJ combinations frequence.
