# Supplementary material for: Annotation of gene loci and analysis of expression diversity in sheep immunoglobulin
Source: Front Immunol. 2025 Aug 29;16:1643380. doi: 10.3389/fimmu.2025.1643380 (PMC12425715; doi:10.3389/fimmu.2025.1643380)
Supplement: Supplementary 1 — The complete nucleotide sequences of V(D)J genes. [file Table1.docx]

>VH1S10 ORF

CAGGTGCGGCTGCAGGAGTCGGGACCCAGCCTGGTGAAGCCCTCACAGACCCTCTCCCTCACCTGCACGGTCTCTGGATTCTCATTAACCAGCAAGGCTGTAGGCTGTGTCCGCCAGCCTCCAGGAAAGGCACCGGAGTGGGTTGGTGGTATAGACAATGATGGAGACACAGGCTATAACCCAGCCCTTAAGTCCCGGCTCAGCATCACCAGGGACACCTCCAAGAGCCAAGTCTCCCTGTCACTGAGCAGCGTGACAACTGAGGACACGGCCGTGTACTACTGT

>VH2S1 P

CAGGTGAAGGTGCAGGAGTCGGGCCCTGACCTGCTGAAGCCCTCGCAGACCGTCTCCCGCACATCTGCTGCCTCTGGTTACTCCATCACGTGGTTATGGAGGGAGCTGGATCCACCAGGCCCCAGGGAAGGGGCTAGAGTGGATGGGAAGCATATATTATAATGGTGACACTCACGACCGCCCCTCCATCAAGAGCCACACCTCCATCTCCAGGGACACGTCCAAGGACCAGTTCTCCCTGCAGCTGAGCTCTGTGACCTCTGTGGACACAGCTGTGTATTACTGT

>VH1S8 F

CAGGTGCGGCTGCAGGAGTCGGGACCCAGCCTGGTGAAGCCCTCACAGACCCTCTCCCTCACCTGCACGGTCTCTGGATTCTCATTAACCAGCAAGGCTGTAGGCTGTGTCCGCCAGCCTCCAGGAAAGGCACCGGAGTGGGTTGGTGGTATAGACAATGATGGAGACACAGGCTATAACCCAGCCCTTAAGTCCCGGCTCAGCATCACCAGGGACACCTCCAAGAGCCAAGTCTCCCTGTCACTGAGCAGCGTGACAACTGAGGACACGGCCGTGTACTACTGT

>VH2S2 P

CAGGTGAAGGTGCAGGAGTCGGGCCCTGACCTGCTGAAGCCCTCGCAGACCGTCTCCCGCACATCTGCTGCCTCTGGTTACTCCATCACGTGGTTATGGAGGGAGCTGGATCCACCAGGCCCCAGGGAAGGGGCTAGAGTGGATGGGAAGCATATATTATAATGGTGACACTCACGACCGCCCCTCCATCAAGAGCCACACCTCCATCTCCAGGGACACGTCCAAGGACCAGTTCTCCCTGCAGCCGAGCTCTGTGACCTCTGTGGACACAGCTGTGTATTACTGT

>VH3S1 P

GGAGGCCCTCTCCCCACCTGGTCTGTCTGTGGATTCTGCATCACACAGAGTGCTGCCTGCTGGGGCTGGATCCAATCAGTCTGAAGAGTCAGAGCTGCAGTGACGGGGTGC

>VH1S6*1 F

CAGGTGCAGCTGCAGGAGTCGGGACCCAGCCTGGTGAAGCCCTCACAGACCCTCTCCCTCACCTGCACGGTCTCTGGATTCTCATTAACCAGCTATCGTGCAGGCTGGGTCCGCCAGGCTCCAGGAAAGGCGCCAGAGTGGGTTGGTGGTACAAGCAGTGGTGGAAGCACAGGCTATAACCCGGCCCTGAAATCCCAGCTCAGCATCACCAGGGACAACTCCAAGAGCCAAGCCTCCCTGTCACTGAGCAGCGTGACAACTGAGGACACGGCCGTGTACTACTGT

>VH1S6*2 P

TGGAAGCACAGGCTATAACCCGGCCCTGAAATCCCAGCTCAGCATCACCAGGGACAACTCCAAGAGCCAAGCCTCCCTGTCACTGAGCAGCGTGACAACTGAGGACACGGCCGTGTACTACTGT

>VH2S3 P

CAGGTGAAGGTGCAGGAGTCGGGCCCTGACCTGCTGAAGCCCTCACAGACCGTCTCCCTCACATCTGCTGCCTCTGGTTACTCCATCACGTGGTTATGGTGGGAGCTGGATCCACCAGGCCCCAGGGAAGGGGCTAGAGTGGATGGGAAGCATATATTATAATGGTGACACTCACGACCGCCCCTCCATCAAGAGCCACACCTCCATCTCCAGGGACACGTCCAAGATCCAGTTCTCCCTGCAGCCGAGCTCTGTGACCTCTGTGGACACAGCTGTGTATTACTGT

>VH3S2 P

GGAGGCCCTCTCCCCACCTGGTCTGTCTGTGGATTCTGCATCACACAGAGTGCTGCCTGCTGGGGCTGGATCCAATCAGTCTGAAGAGTCAGAGCTGCAGTGACGGGGTGC

>VH4S1 P

CAGGTGCAGCTGGTGCTCTTGGGGCTGAGCTGAGGAAGCCTGGGGATCAGTGAAGGGGTCCTGCGAGGCTTCTGATACACCTTCACCGACTACTACAGGCACTGGGTGTGACAGGCCCCTCAACAAGGGCTTGAACGGATGGACAGATGGACAGCAAGGATGGTGGAACAAAGTATGCACAGCAGTTCCAGAGCAGAGTCACGCTGGCTGCAGACACGTCCACCGGCACCGCCTACATGGAGCTGAGCAGTCCGAGGTCTGAGGACACGGCTGTTTATTACTGT

>VH1S1*1 F

CAGGTGCGGCTGCAGGAGTCGGGACCCAGCCTGGTGAAGCCCTCACAGACCCTCTCCCTCACCTGCACGGTCTCTGGATTCTCATTAACCAGCAATGCTGTAGGCTGGGTCCGCCAGGCTCCAGGAAAGGTGCCGGAGTGGCTTGGTGGTATAAGCAGTGGTGGAAGCACATACTATAACCCGGCCCTGAAATCCCGGCTCAGCATCACCAGGGACACCTCCAAGAGCCAAGTCTCCCTGTCACTGAGCAGCGTGACAACTGAGGACACGGCCGTGTACTACTGT

>VH2S4 ORF

CAGGTGAAGGTGCAGGAGTCGGGCCCTGACCTGCTGAAGCCCTCACAGACCGTCTCCCTCACATCTGCTGCCTCTGGTTACTCCATCACGTGGTTATGGAGGGAGCTGGATCCACCAGGCCCCAGGGAAGGGGCTAGAGTGGATGGGAAGCATATATTATAATGGTGACACTCACGACAGCCCCTCCATCAAGAGCCACACCTCCATCTCCAGGGACACGTCCAAGGACCAGTTCTCTCTGCAGCTGGGCTCTGTGACCTCTGTGGACACAGCTGTGTATTACTGT

>VH1S1*2 F

CAGGTGCGGCTGCAGGAGTCGGGACCCAGCCTGGTGAAGCCCTCACAGACCCTCTCCCTCACCTGCACGGTCTCTGGATTCTCATTAACCAGCTATGGTGTAGGCTGGGTCCGCCAGGCTCCAGGAAAGGCGCTGGAGTGGCTTGGTGGTATAAGCAGTGGTGGAAGCACAGGCTATAACCCGACCCTGAAATCCCGGCTCAGCATCACCAGGGACACCTCCAAGAGCCAAGTCTCCCTGTCACTGAGCAGCGTGACAACTGAGGACACGGCCGTGTACTACTGT

>VH2S5 P

CAGGTGAAGGTGCAGGAGTCGGGCCCTGACCTGCTGAAGCCCTCACAGACCGTCTCCCTCACATCTGCTGCCTCTGGTTACTCTATCACGTGGTTATGGAGGGAGCTGGATCCACCAGGCCCCAGGGAAGGGGCTAGAGTGGATGGGAAGCATATATTATAATGGTGACACTCACGACAGCCCCTCCATCAAGAGCCACACCTCCATCTCCAGGGACATGTCCAAGAACCAGTTCTGCCTGCAGCTGAGCTCTGTGACCTCTGTGGACACAGCTGTGTATTACTGT

>VH3S3 P

CAGGTGCTGGTGCGGGAGTCGGAGCAGGACTATTGGAGGCCCTCTCCCCACCTGGTCTGTCTGTGGATTCTGCATCACACAGAGTGCTGCCTGCTGGGGCTGGATCCAATCAGTCTGAAGAGTCAGAGCTGCAGTGACGGGGTGC

>VH2S6 P

CTGCAGCAGTCGGGCCCAGAGCTGGTGAAGCCCTCACTGACCCTCTCCTCACGTGCGCTGTCTCTGCTTACTCCATCACGTGCTTATAGTGGGAACTGGATTCGCCAGGCCCCAGGGACGGGGCTAGAGCAGACGGCATGCATCACCGATGATGGTGACACTTACTACACCCCCTCCATCAAGAGCCACGCCTCCATCTGCAGAGACACCCAAGAACCAGTTCTCCCTGCAGCTGAGCTCTGTGACCACTGAGGACACGGCCGTGT

>VH1S5 F

CAGGTGCGGCTGCAGGAGTCGGGACCCAGCCTGGTGAAGCCCTCACAGACCCTCTCCCTCACCTGCACGGTCTCTGGATTCTCATTAACCAGCTATGGTGTAGGCTGGGTCCGCCAGGCTCCAGGAAAGGCGCCGGAGTGGGTTGGTGGTATAGATAATGATGGAGACACAGGCTATAACCCAGCCCTTAAGTCCCGGCTCAGCATCACCAGGGACACCTCCAAGAGTCAAGTCTCCCTGTCACTGAGCAGCGTGACAACTGAGGACACGGCCGTGTACTACTGT

>VH2S7 ORF

CAGGTGAAGGTGCAGGAGTCGGGCCCTGACCTGCTGAAGCCCTCACAGACCGTCTCCCTCACATCTGCTGCCTCTGGTTACTCCATCACGTGGTTATGGAGGGAGCTGGATCCACCAGGCCCCAGGGAAGGGGCTAGAGTGGATGGGAAGCATATATTATAATGGTGACACTCACGACAGCCCCTCCATCAAGAGCCACACCTCCATCTCCAGGGACTCGTCCAAGAACCAGTTCTCTCTGCAGCTGAGCTCTGTGACCTCTGTGGACACAGCTGTGTATTACTGT

>VH1S4 F

CAGGTGCAGCTGCAGGAGTCGGGACCCAGCCTGGTGAAGCCCTCACAGACCCTCTCCCTCACCTGCACGGTCTCTGGATTCTCATTAACCAGCTATGGTGTAGGCTGGGTCCGCCAGGCTCCAGGAAAGGCACTGGAGTGGGTTGGTAACATATATAGTGGTGGAAGTACATACTATAACCCGGCCCTGAAATCCCGGCTCAGCATCACCAGGGACACCTCCAAGAGCCAAGTCTCCCTGTCACTGAGCAGCGTGACAACTGAGGACACGGCCGTGTACTACTGT

>VH2S8 P

CAGGTGAAGGTGCAGGAGTCGGGCCCTGACCTGCTGAAGCCCTCACAGACCGTCTCCCTCACATCTGCTGCCTCTGGTTACTCCATCACGTGGTTATGGAGGGAGCTGGATCCACCAGGCCCCAGGGAAGGGGCTAGAGTGGATGGGAAGCATATATTATAATGGTGACACTCACGACCGCCCCTCCATCAAGAGCCACACCTCCATCTCCAGGGACACGTCCAAGATCCAGTTCTCCCTGCAGCCGAGCTCTGTGACCTCTGTGGACACAGCTGTGTATTACTGT

>VH3S4 P

GGAGGCCCTCTCCCCACCTGGTCTGTCTGTGGATTCTGCATCACACAGAGTGCTGCCTGCTGGGGCTGGATCCAATCAGTCTGAAGAGTCAGAGCTGCAGTGACGGGGTGC

>VH2S9 P

ACCCTGGCACTGCAGCAGTCGGGCCCAGAGCTGGTGAAGCCCTCACTGACCCTCTCCTCACGTGCGCTGTCTCTGCTTACTCCATCACGTGCTTATAGTGGGAACTGGATTCGCCAGGCCCCAGGGACGGGGCTAGAGCAGACGGCATGCATCACCGATGATGGTGACACTTACTACACCCCTCCATCAAGAGCCACGCCTCCATCTGCAGAGACACCCAAGAACCAGTTCTCCCTGCAGCTGAGCTCTGTGACCACTGAGGACACGGCCGTGT

>DH1

AGAATACCATGATGATAGCTACTGCTATAGC

>DH2

GTAGTTATTATAGTGGTTATGGTTATGCTTATGGTTATGC

>DH3

GTGATATGATAGGTATGGCTGTAGTTACTGTAGTGTCGCTAC

>DH4

GTAGTTATTATAGCGATTATGGTTATGC

>JH1

ACTATGCTGACTTCCATCTCTGGGACCAGGGTGCCCTGGTCACCGTCTCCTCAG

>JH2

GCTGCTGGGACTCGGGTCTCTGGGGCCAGCGCACCCCGGTCACCGTGTCCTTGG

>JH3

TTGCTTTTGACTCCTGGGGCCAGCGCGCCCCGGTCACAGTCTCCTCAG

>JH4

ACTATATCGACTACTGGGGCCCAGGACTCCTGGTCACCGTCTCCTCAG

>JH5

TAACGACTGGCTCAAGCACTGGGGCCAGGGACCCCGACGCTGTCTGCTCA

>JH6

ATTACTACGGTGTAGATGTCTGGGGCCGAGGACTCCTGGTCACCGTCTCCTCAG

>μ1

CH1

AAAGTGAATCTCACCCGAAAGTCTTCCCCCTGGTGTCCTGTGTGAGCTCCCCGTCTGATGAGAACACGGTGGCCCTGGGCTGCCTGGCCCGGGACTTCGTGCCCAATTCTGTCAGCTTCTCCTGGAAGTTCAACAACAGCACGGTCAGCAGCGAGAGGTTCTGGACCTTCCCCGAAGTGCTGAGGGACGGCTTGTGGTCGGCCTCCTCTCAGGTGGCCCTGCACTCCTCAAGCACCTTTCAAGGGACGGATGGCTACCTGGTGTGTGAAGTCCAGCACCCCAAGGGAGGAAAGACCGTCGGGACCGTGATGGTGGTCGCTCCAA

CH2

AGGTGGAAGTGTTGTCCCCCGTCGTGAGTGTCTTTGTCCCGCCTTGCAACAGCCTCTCTGGTAACGGCAATAGCAAGTCCAGCCTCATCTGCCAGGCCACTGACTTCAGCCCCAAACAGATCTCCTTGTCCTGGTTTCGTGATGGAAAGCGGATAGTGTCTGATATTTCTGAAGGCCAGGTGGAGACTGTGCAGTCCTCACCCACAACATACAGGGCCTACAGCGTGCTGACCATCACGGAGCGAGAATGGCTCAGCCAGAGCGCATACACCTGCCAGGTGGAGCACAACAAGGAAACCTTCCAGAAGAACGCGTCCTCTTCGTGTGATGCTA

CH3

CACCACCGTCTCCCATCGGGGTCTTCACCATCCCCCCATCCTTTGCCGACATCTTCCTCACGAAGTCAGCCAAGCTTTCCTGTCTGGTCACAAACCTGGCTTCCTATGATGGCCTGAACATCAGCTGGTCCCATCAGAATGGCAAGGCCCTGGAGACCCACACTTATTTTGAGAGACACCTCAACGACACCTTCAGCGCCAGGGGCGAGGCCTCAGTCTGCTCGGAGGACTGGGAGTCCGGAGAGGAGTACACCTGCACAGTGGCCCACTTGGACCTGCCCTTCCCAGAAAAGAGCGCTATCTCCAAGCCCAAAG

CH4

ACGTCGCCATGAAACCGCCGTCCGTGTACGTGCTGCCTCCAACGCGGGAACAGCTGAGCCTGCGGGAGTCAGCCTCCGTCACCTGCCTGGTGAAGGGCTTCGCGCCCGCGGACGTGTTCGTGCAGTGGCTGCAGAAGGGGGAGCCTGTGGCCAAGAGCAAGTACGTGACAAGCAGCCCGGCGCCCGAGCCCCAGGACCCCAGCGCGTACTTCGTGCACAGCATCCTGACAGTGACCGAGGAGGACTGGAGCAAAGGGGAGACCTACACCTGCGTCGTGGGCCACGAGGCCCTGCCCCACATGGTCACCGAGCGGACCGTGGACAAGTCCACCGGTAAACCCACCCTGTACAACGTGTCCCTGGTCATGTCTGACACGGCCAGCACCTGCTACTGA

M1

AGGGGGAGGTGAGCGCCGAGGAGGAAGGCTTCGAGAACCTCAACACCATGGCCTCCACCTTCATTGTGCTCTTCCTCCTGAGCCTCTTCTACAGCACCACGGTCACCCTGTTCAAG

M2

GTGAAGTGA

>δ

CH1

AAAGTGAATCTCACCCGAAAGTCTTCCCCCTGGTGTCCTGTGTGAGCTCCCCGTCTGATGAGAACACGGTGGCCCTGGGCTGCCTGGCCCGGGACTTCATGCCCAATTCTGTCAGCTTCTCCTGGAAGCTCAACAACAGCACGGTCAGCAGCGAGAGGTTCTGGACCTTCCCCGAAGTGCTGAGGGACGGCTTGTGGTCGGCCTCCTCTCAGGTGGCCCTGCACTCCTCAAGCGCCTTTCAAGGGACGGATGGCTACCTGTGTGAAGTCCAGCACCCCAAGGGAGGAAAGACCGTCGGGACCACAAGGGTGGTCCCCAGAG

Hinge1

TTTCTGCATCAACTCTGACCCCGACCACGCTAGCACCGTCTCTGAAATCCAGGTCAGAGGGCTCCAGTAAAGCCGTCACCACTCAGAGCAGCCCAG

Hinge2

TGCCGGCCACCAGCCACAGCCAGACAGAAGCCCCGACACTAGCGTGTCCAAAGGACCCCTGCAGAG

CH2

AGTGTCAGAACCACACCCAGGCCCCCAGCGTCCGCCTGCTGCCCCCGCCCCCGCAGGGCCTCTGGCTTTTGGACAAGGCAGAGTTCACCTGCCTGGCCACAGGGGAGGCCCTGCTGGATGCCCACTTCTCCTGGGAGGTGAACGGGCAGCCCCATGGCGGGGCCGTGGAGGAGAGACCCACCTCGCACATGAACGGCTCCTGGAGCCACAGCAGCCGCCTGGCCCTGCCCAGGTCCCTGTGGGCCTCGGGCTCCAACGTCACCTGCACACTGAGCGGCCCTGGCCTGCGGTCACCAGTGTCCCTGACGGCACAGAGAGAGCATG

CH3

CTGCCTCAGTGCCCGGCAATCTGACCCTCCGCACTCTGACCACGCCTGGCCCCTTCTCCCCTGCCTGGCTCCTGTGCGAGGTGTCCGGCTTCTCACCCGTGGACATCCTCCTCACATGGCTGGAGGGCCAGCAAGAAGTGGAGCCTTCCCAGTTTGCCACAGCGCACACCACAGCCCAGTCTGGGCATGCCTCATTCCACACCTGGAGTGTCCTACATGTCTCCAGCCCCCTGGACCATGTGGGGTCCACCTACACCTGTGTGGTCAGCCACGAGGCCTCCCGGACGCTCCTCAATGGCAGCTGCAGCCTGGACACTGGTG

Sec

CTCTTTCCCGCCTTCTCATCCCATGTGTCTGAGAAGACCTCCCTGACCTCCATAGCTGGGCCATGGAGCATCCAGGGGGTCCTGTCATCGCATTTAGCCACCTCACCGACCCCCAGACCACTCACCCCTGCATCCCGTGTGTCTAG

M1

GCCTGGCCACCTGGCCGCCCTGGAGCCAGGACGAGAGCAGCGACGACAGCGCAGATGCAGAGGACGCCAGCCCCCTCTGGCTCACCTTCCTGGCCCTCTTCCTCGCCACTGTGGTCTACAGCGGCTTCGTCACCTTCATCAAGGCAG

M2

GTGAAGTAG

>γ1

CH1

CCTCCACCACACCCCCGAAAGTCTACCCTCTGGCATCCAGCTGCGGGGACACGTCCAGCTCCACCGTGACCCTGGGCTGCCTGGTCTCCAGCTATATGCCCGAGCCAGTGACCGTGACCTGGAACTCGGGTGCCCTGACCAGCGGCGTGCGCACCTTCCCGGCCATCCTGCAGTCCTCCGGGCTCTACTCTCTCAGCAGCGTGGTGACCGTGCCGGCCAGCACCTCAGGAGCCCAGACCTTCACCTGCAACGTAGCCCACCCGGCCAGCAGCACCAAGGTGGACAAGCCTGTCACTGCAAGGCCTCGGGTCCTGCAGACAACGCCAGGGTCACCTATCCGTACTGTAAGACCCACGACCCAGG

Hinge 1

AGCCCAAAGGTGAACAGAAACCCTGCCAGTGTCCCAAATGCCCAG

CH2

CCACAGAACCTCTGGGAGGACTGTCTGTCTTCATCTTCCCACCGAAACCCAAGGACACCCTTACAATCTCTGGAACGCCCGAGGTCACGTGTGTGGTGGTGGACGTGGGCCAGGATGACCCCGAGGTGCAGTTCTCCTGGTTCGTGGACAACGTGGAGGTGCGCACGGCCAGGACAACGCCGAGAGAGGAGCAGTTCAACAGCACCTTCCGCGTGGTCAGCGCCCTGCCCATCCAGCACCAAGACTGGCTGCGGGGCAAGGAGATCAAGTGCAAGGTCCACAACAAAGCCCTCCCGGCCCCCATCGTGAGGACCATCTCCAGGACCAAAG

CH3

GGCAGGCTCGGGAGCCGCAGGTGTACGTCCTGGCCCCACCCCGGGAAGAGCTCAGCAAAAGCACGCTCAGCGTCACCTGCCTCATCATCGGTTTCTACCCAGAAGAGGTAGACGTGGAGTGGCAGAGAGATGGGCAGCCTGAGTCGGAGGACAAGTACCACACAACCCCGCCCCAGCTGGACGCCGACGGCTCCTACTTCCTGTACAGCAGGCTCAGGGTGAATAAGAGCAGCTGGCAGGAAGGAGACACCTACACGTGTGCGGTGATGCATGAAGCTCTACGGAATCACTACAAAGAGAAGTCCATCTCTAAGTCTCCGGGTAAATGA

TM1

ATCTGCTGCTGGAGGAGGAGAGCTGTGCGGACGACCTGGACGGGGAGCTGGACGGGCTCTGGACGACTATCTCCATCTTCATCACGCTCTTCCTGCTCAGCGTCTGCTACAGCGCCACCGTGACCCTCTTCAAG

TM2

GTGAAATGGATCTTCTCCTCAGCAGTGGAGCTGAAGAGGACGATGGTCCCCGACTACAGGAACACGATTGGGCAGGGCGCCTAG

>γ2

CH1

CCTCCACCACACCCCCGAAAGTCTACCCTCTGACTTCTTGCTGCGGGGACACGTCCAGCTCCATCGTGACCCTGGGCTGCCTGGTCTCCAGCTATATGCCCGAGCCGGTGACCGTGACCTGGAACTCTGGTGCCCTGACCAGCGGCGTGCACACCTTCCCGGCCATCCTGCAGTCCTCCGGGCTCTACTCTCTCAGCAGCGTGGTGACCGTGCCGGCCAGCACCTCAGGAGCCCAGACCTTCATCTGCAACGTAGCCCACCCGGCCAGCAGCACCAAGGTGGACAAGCGTGTTG

Hinge 1

AGCCCGGATGCCCGGACCCATGCAAACATTGCCGATGCCCAC

CH2

CCCCTGAGCTCCCCGGAGGACCGTCTGTCTTCATCTTCCCACCGAAACCCAAGGACACCCTTACAATCTCTGGAACGCCCGAGGTCACGTGTGTGGTGGTGGACGTGGGCCAGGATGACCCCGAGGTGCAGTTCTCCTGGTTCGTGGACAACGTGGAGGTGCGCACGGCCAGGACAAAGCCGAGAGAGGAGCAGTTCAACAGCACCTTCCGCGTGGTCAGCGCCCTGCCCATCCAGCACCAAGACTGGACTGGAGGAAAGGAGTTCAAGTGCAAGGTCCACAACGAAGGCCTCCCGGCCCCCATCGTGAGGACCATCTCCAGGACCAAAG

CH3

GGCAGGCCCGGGAGCCGCAGGTGTACGTCCTGGCCCCACCCCAGGAAGAGCTCAGCAAAAGCACGCTCAGCGTCACCTGCCTGGTCACCGGCTTCTACCCAGACTACATCGCCGTGGAGTGGCAGAAAAATGGGCAGCCTGAGTCGGAGGACAAGTACGGCACGACCACATCCCAGCTGGACGCCGACGGCTCCTACTTCCTGTACAGCAGGCTCAGGGTGGACAAGAACAGCTGGCAAGAAGGAGACACCTACGCGTGTGTGGTGATGCACGAGGCTCTGCACAACCACTACACACAGAAGTCGATCTCTAAGCCTCCGGGTAAATGA

TM1

ATCTGCTGCTGGAGGAGGAGAGCTGTGCGGACGCCCAGGACGGGGAGCTGGACGGGCTCTGGACGACTATCTCCATCTTCATCACGCTCTTCCTGCTCAGCGTCTGCTACAGTGCCACCGTGACCCTCTTCAAG

TM2

GTGAAGTGGATCTTCTCATCAGTGGTGGAGCTGAAGAGGACCATCGTCCCCGACTACAGAAATATGATCGGGCAGGGCGCCTAG

>γ3

CH1

CCTCCACCACACCCCCGAAAGTCTACCCTCTGACTTCTTGCTGCGGGGACACGTCCAGCTCCATCGTGACCCTGGGCTGCCTGGTCTCCAGCTATATGCCCGAGCCGGTGACCGTGACCTGGAACTCTGGTGCCCTGACCAGCGGCGTGCACACCTTCCCGGCCATCCTGCAGTCCTCCGGGCTCTACTCTCTCAGCAGCGTGGTGACCGTGCCGGCCAGCACCTCAGGAGCCCAGACCTTCATCTGCAATGTAGCCCACCCGGCCAGCAGCACCAAGGTGGACAAGCGTGTTG

Hinge 1

GGATCTCCAGTGACTACTCCAAGTGTTCTAAACCGCCTT

CH2

GCGTGAGCCGACCGTCTGTCTTCATCTTCCCCCCGAAACCCAAGGACAGCCTCATGATCACAGGAACGCCTGAGGTCACGTGTGTGGTGGTGGACGTGGGCCAGGATGACCCCGAGGTGCAGTTCTCCTGGTTCGTGGACAACGTGGAGGTGCGCACGGCCAGGACAAAGCCGAGAGAGGAGCAGTTCAACAGCACCTTCCGCGTGGTCAGCGCCCTGCCCATCCAGCACGACCACTGGACTGGAGGAAAGGAGTTCAAGTGCAAGGTCCACAGCAAAGGCCTCCCGGCCCCCATCGTGAGGACCATCTCCAGGACCAAAG

CH3

GGCAGGCCCGGGAGCCGCAGGTGTACGTCCTGGCCCCACCCCAGGAAGAGCTCAGCAAAAGCACGCTCAGCGTCACCTGCCTGGTCACCGGCTTCTACCCAGACTACATCGCCGTGGAGTGGCAGAGAGCGCGGCAGCCTGAGTCGGAGGACAAGTACGGCACGACCACATCCCAGCTGGACGCCGATGGCTCCTACTTCCTGTACAGCAGGCTCAGGGTGGACAAGAGCAGCTGGCAAAGAGGAGACACCTACGCGTGTGTGGTGATGCACGAGGCTCTGCACAACCACTACACACAGAAGTCGATCTCTAAGCCTCCGGGTAAATGA

TM1

ATCTGCTGCTGGAGGAGGAGAGCTGTGTGGACGCCCAGGACGGGGAGCTGGACGGGCTCTGGACGACTATCTCCATCTTCATCACGCTCTTCCTGCTCAGCGTCTGCTACAGCGCCACCGTGACCCTCTTCAAG

TM2

GTGAAGTGGATCCTCTCATCTGTGGTGGAGCTGAAGCAGTCAATCACTCCCAACTACAGAAACATGATTGGACAGGGCGCCTAG

>ε

CH1

TCTCCAGCCAGCATCCATCCATCTACCCCTTGAGGACCTGTTGCATAAAAGAAACCTCTGTGGCCCTGGGCTGCCTGGTCAAGGACTACTTCCCAGGGTCAGTGGCTGTGACCTGGGACACAGCGCCCCTGGACGGGAGCACCCTGACCTTCCCTAGCATCCAAATGGCGAACTCCAGTCTCTACGTCACCACCAGCCAGCTGACCGTCTCGGGCGAGCAGCCCAAGCAGTTCACCTGCAGTGTTTTCCACGCTGAGACTAACACCACTGCCATGAAGACCGTCAGCACTG

>CH2

AGTGCGCCAAGAACTTCAGCGACCCCTCGGTGAGGCTCTTCTACTCCTCCTGTGACCCCAGCGGCGACACCCACACCACCATCCAGCTCCTGTGCCGGATCTCCGGCTACACCCCAGGCAAGATCAAGGTCACCTGGCTGGTGGACGGGCACGAGTCCAAAGAACTGTATGCACAGCCCGGCCCCGAGATACAGGAGGGCAATCTGACCACCACCTACAGCGAAGTCAACATCACACAGGGCCAGTGGGTGTCCGAGAAAACCTACACCTGCCGGGTCAACTATTACGGCTTCAACTTCGACAACCACGCCCGCAGGTGCACAG

>CH3

CCGAGTCCGAGCCCCGCGGCGTGAGCACCTACCTGATCCCACCCACCCCCCTCGAGCTGTACGTCAACAAGTCGCCCAAGATCACCTGCCTGGTTGTGGACCTGGCCAGCACGAATAACTTGAGCCTGACCTGGTCGAGGGCAAACGGGAAACCTGTGCACGCAGACCCCCTCGACATCAAGCATCAGTTCAACGGGACGATCACCGTCACGTCCACCCTGCCGGTGAACGTCATTGACTGGGTTGAAGGCGAGACCTACTACTGCAAAGTGAGCCACGGCGACCTGCCCAAGGATATCCAGAGATCCATCTCCAAGGATGTTG

>CH4

GCAAGCGTGTGGCCCCCAAGGTCTACGTGTTCTGGCCAGACAGAAAGGAGCTGGAGAATCAGGAGGAGCTCACCCTCACCTGCCTGATCCAGAAATTCTTCCCCAAGGACATCTCTGTGCGGTGGCTGCGTAACAAGGAACTGATGCGGGAAGGCCAGCACACCACCACGCAGCCCGACAGGGCCGACAACAACAGCCTCGCCTTCTTCGCCTACAGCCGCCTGGCGGTCCCCAAGGCCAGCTGGAAGATGGACGATGAGTTCACCTGCCAAGTGATCCATGAGGCACTGCCCAAGACTAGGACCCTGGAGAAATCGGTGTTCATTAACAGCGGTAAATAA

>TM1

AGCTGGCGCTCGAGGACCTGTGTGCGGAGGAAGCTGAGAGCGAGGAGCTGGAGGAGACCTGGACCAGCCTCCTTGTCTTCATCGTGCTCTTCCTGCTCAGCGTGAGCTATGGCGCCACCGTCAGCCTCTGCAAG

>TM2

GTGAAGTGGGTCCTGGACGCCATCCTCCAGGGGCAGCCCCAGGGCTCCCAAGACTACACCAATGTCACGCAGCGCCCGCTGCCTCCTGGCGTCTGA

>α

CH1

AGAGTGAAGCCAGCCCCAGCATCTTCCCACTGAGCCTTGGGAAGGACGACCCAGCCGGGCAGGTGGTCATCGGCTGCCTGGTCCAGGGCTTCTTCCCATCAGCACCTCTGAGCGTGACCTGGAACCAGAGTGGTGACAATGTGTCCGTCAGGAACTTCCCCGCCGTGCTGGCCGGGAGCCTGTACACCATGAGCAGCCAGCTGACCTTGCCAGCCAGCCTGTGCCCAGAAAACAAGTTCGTGACCTGCCAAGTGCAGCACCTCTCCAAAGCCAGCAAGACCGTGGACGTGCCCTGCAAACTTGAAG

Hinge 1

GTTGTCCTGAGGAGTGTC

CH2

TCCCATTGAACTGCGAGCCCAGCCTGTCCCTGCAGCCTCCAGCCCTCGAGGATCTGCTCCTGAGCTCCAACGCCAGCCTCACGTGCACGCTGCGTGGCCTGAAAAGCGCCAAGGGCGCCAGCTTCACCTGGAGCCCGACAGGCGGGAAGACCGCCGTCCAGGGGCTGCCCGAGCGCGACTCCTGCGGCTGCTACAGCGTGTCCAGCGTCCTGCCGGGCTGTGCCGACCCCTGGAACAGCGGACAGACTTTCTCCTGCTCTGCCACCTACCCCGACGGCTCACTGACCACCACCATCAAGAAAGACCTAG

CH3

TGAACACGTTCCGGCCTCAGGTCCACCTGCTGCCGCCGCCGTCGGAGGAGCTGGCCCTCAACGAGCTGGTGACGCTGACGTGCCTGGTGCGGGGGTTCAGCCCCAGGGACGTGCTGGTGCGCTGGCTGCAGGGCAATCAAGAGCTGCCCCGCGAGAAGTATCTGACCTGGGGACCCCTGCCCGAGCCCGGCCAGAGTGTCACCACCTTCGCCGTGACCAGCGTGCTACGCGTGGACGCCGAGGTCTGGAAGCAGGGGGACACCTTCTCCTGCATGGTGGGCCACGAGGCCCTGCCCCTGGCCTTCACCCAGAAGACCATCGACCGCCTGGCGGGTAAACCCACCCACGTGAACGTGTCTGTGGTCATGTCAGAGGTGGATGGGGTCTGCTACTGA

TM1

CCCTGGCTGGTGCTGGACCTGATGCAGGGCAGCCCCGAGGAGGACAGCCTGGAGGACAGCCTGTGGCCCACGACCGTCACCCTGCTCACCCTCTTCCTGCTGAGCCTCTTCTACAGCACAGCGCTGACCGTGACGAGCATCCGGGCCACACCGGACAGCCGGCAGGTCCCCCAGTACTGA

>Vκ1-21 ORF

GACCTCCAGATGACCCAGTCTCCATCTTCCCCGTTTGCATCTCTAGGAGATGCCATCACTTGGCAGGCCAGTCAGAGCATTAGCAAGTACTTAGCTTGGTATCAACAGAAACCAGGGAAAGCTCCTAAGCTCCTGATCTATCATGCATCCAGACTGCACACAGGGGTCCCATCCTGGTTCAGTGGCAGTGGATCTGGGACAGATTACACCCTCACCATCAGCAAGCTGGAGGCTGATGATGTTGCAACTTATTACTGT

>Vκ10-1 P

GAACTTATGTCGAAGCAGTCTCCAGCCTCCCTGTCTTTGACTCCAGCAGAAATAGCCACCCTCACCTGCAGGGCCAGTCAGAGTATTAGTAGCTACTTGGACTGGTACCAGCAGAAGCCTGGGCAGGCCCCCAGACTCTTCATCTATGCTGCCTCCAGCAGGGCCTCTGGCATCCCAGCCCGGTTCAGTGACAGTTGGGTCAGGGACAGACTTTACTCTCCCCATCAGCAGCCTGGAGCCTGAAGATGTTGCAGTTCATTTCT

>Vκ11-1 P

CCGTGGAGGGAGCGTCTCCATCTCCTACAAGGCCATCTCATCTCATCCTCAGTAATGGACACACACCCCCACCCTGCTTCCAGCACAAACCACACACTCTCCTCCCCCGCTGATCACAGGGGGTCCCTCCACCTCCCGGGGTCCCAGCCCAGCCCAGTGGCCGTGGGACTGGGATGGATTTCACACTCACACTCATCAGTGTGGAGACTGCGGACTCTGCACATGACTTCTGT

>Vκ2-16 P

TGCTGACCCAGACTCCACTCTCCCTGTCTATCATCCCTGGAGAATGGCCTCCATCTCCTGCAAGTCTAGACGGAGCCTTCTATATAGTGATGGAAACACCTATTTGTACTGGTTTCAGCATAAACCAGGCCAGTCCCCACAGCGACTGATCTATCAGGTTTCCAGCCGTGACACTGGGGTCTCAGACAGGTTCACTGGCAGTGGGTCAGGGACAGACTTCACACTTGCAGTCTGCAGAGTACAGGCTGAGGATGCTGGAGTCTATTACTGT

>Vκ1-17 ORF

GACCTCCAGATGATTCAGTCTCTATCCTCCCTGTCTGCATCTCTAGGAGACAGAGTCTCCATCACTTGTCAGGCCAGTCAGAGTGTTAGAAATAATTTACAGTGGTATCAAGAGAAACCAGGGAAAGCTCCTAAGTTCCTCATCTGTGACACAACCAGTGTGCACACAGGGGTCCCATCCCGGTTCAGTGGCAGTGGATCTGGGACAGATTACACCTTCACCACCAGCAGCCTGGAGGCTGACGATTTTGCAGCTTATTAGTGT

>Vκ11-2 P

CTCCATCTCCTGCAAGGCCATCTCATCCCATCCATAGTAATGGACACACCTGCCCTCCCTCCTTCCTGAACAAACCACACATTCTCCTCACCCACTGACATAAGGGGTCCCCCAGGCTCCTGGGGTCTCATCCTGGCTCAGTGGCCATGGGTCTGGGAGGGATTTCACACTCAGATTCACCAGTGTGGAGACTGGGGACCCTGAACATGACTTCTGTGCACAGAGCCTTCAATGGGAACTGTACTTGCAC

>Vκ2-15 F

GCTATTGTTCTGACCCAGACTCCACGCTCCCTGTCTGTCATCCCTGGAGAGACGGCCTCCATCTCCTGCAGAGCAAGTCAGAGCGTTCAAAATAGATACGGAGACAATTTTTTGCACTGGTATGTGCAGAAGCCCAGCCAGTCTCCGCAGCTCCTGATCTATGCAGCTTCTAACCGTGCCTCTGGAGTCTCAGACAGGTTCACTGGCAGTGGGTCAGGGACAGATTTTACCCTCAAAATCAGCAGGGTGGAGGCTGAGGATGCTGGAGTTTATTATTGC

>Vκ2-14 F

GATGTTGTGCTGACCCAGACTCCCCTCTCCCTGTCTGTCATCCCTGGAGGGACGGTCTCTATCTCCTGCAAGTCTTCGCAGAGTCTGAAATATAGTGATGGAAACACCTATCTGTACTGGTTTCAACATAAACCAGGCCAGTCTCCACAGCTATTGATCTATCAGGTGTCCAACCGTTACACTGGGGTCCCAGACAGGTTCACTGGCAGTGGGGCAGAGACAGATTTCACACTTACAATCAGCAGTGTGCAGGCTGAGGATGCTGGAGTCTATTACTGT

>Vκ11-3 P

TGTGCTGACTCCATCTCCTGCCTCTGGCCTGGGGCCATGGAGGGAGCGTCTCCATCTCCTGAAGGCCATCTCATCTCATCCACAGTAATGGACACACCTGCCCACCATGCTTCCGGCACAAACCACACACTCTCCTCAATCACTGATCATAGGGGTTCCTGGGGTCCCAGCCTGGCTCAGTGACTGTGCGTCTGGGGCAGATTTCACACTCACACCCACCAGGGTGGGGTCCCTGCATGTGACCTCTGTGCACAGA

>Vκ2-9 P

GCTATTACGCAGACACAGACTCTACGCTCCCTGTCTGTCATCCCTGGAGAGACGGCCTCCATCTCCTGCAAAGCCAATCAGAGCGTTCAAAATAGATATGGAGACAATTTTTTGCACTGGTATGTGCAGAAGTCCAGCCACGCTCCACAGCTCATGATTTATAGGGCTTCTAACTGGGAGTCTTAGGTCCCAGACAGGTTCACAAGCAGTGGGTCAGAGACAGATTTCATCCTCAAAATCAGCAGGGTGGAGGCTGAGGATGGTGAAATTTACTACTGC

>Vκ2-8 F

GATGTTGTGCTGACCCAAACTCCACTCTCCCTGTCTGTCATCCCTGGGGAGACGGCCTCCATCTCCTGCAAGTCTAGTCAGAGCCTGGTACATAGTGATGGAAATACCTATTTGAATTGGATTCAACATAAACCAGGCCAATCACCACAGGGTCTGATCTATCAGGTTTCCAACCGTTACTCTGGAGTCTCAGACAGGCTCACTGGCAGTGGGTCAGGGACAGATTTCACATTTACAATCAGCAGTGTGCAGGCTGAGGATGCTGGAGTCTACTACTGT

>Vκ1-7 P

GACCTCCAGATGACTCAGTCTCCATCCCTGTCTGCCTCTCTAGGAAACAGAGTATCCATCATTTGCTGGGCCAGTCAAAGCATTAGCAAATGGTTAGACAGGTATCAGCAGAAATCAGGGTGAGTTCCTAAGCTCCTGGTCTATGCAGCATCCAGTTTGGGAACTGGGGCCCCCTCCCGGTTCAGTGACAGTGGATCTGGGGCAGATTTCACTCTCACCACGAGCAGCCTGGAGGCTGAAGATGCTGCAACTTATTACTG

>Vκ1-6 P

GACATCCAGATGACCTAGTCTCCATCCATCCTGTCTGCATCTCTAGGAGACAGAGTTTCCATCACTTGCCAGGCCAGTCAGAGTGTTAGCAAATGGTTAGCTTGGTATCAGCAGAAACCAGGGCAAGCTCCTAAGCTGCTCACCTATGTAGCATCCAATTTGCAAACCGGGGTCTCATCCAGGTTTGGTGGCAGTGGATCTGGGACAGACTTCACTGTTGCCATCAGCAGCCTGGAGGCTGAAGATGTTGCAACTTATTACTGT

>Vκ6-5 F

GAGATTATTCTGACCCAGTCTCCAGCCCTCCTGTATAAAACTTCAGGGGAGGAAGCCGCCATCACCTGCTGGGCCAGCCAGGACACTGACAGCAGCTTGCACTGGTACCAGCAGAAATCAAATCAGGCTCCCAAGTTCCTTATAAAGTATGCCTCCCAGTCCATCTCATGGATCCCATCTCAGGTCAGCAGAAGTGGATCTGGGACAGATTTCACCCTCACCATCACTAGTCTCGAAGCTGAAGATGCTGCAACGTCTTACTGT

>Vκ1-4 F

GACATCCAGGTGACCCAGTCTCCATCCTCCCTGTCTGCATCTCTAACAGAGAGAGTCTCCATCACTTGCCGGACCAGTCAGAGCGTTAGCAACTACTTAAACTGGTATCAACAGAAACCAGGGCAAGCTCCTAAGCTCCTGATCTATTATGCAACCAGATTGCACACCGATGTCCCATCCCGGTTCAGTGGCAGTGGATCTGGGACAGATTACACCCTCACCATCAGCAGCCTGGAGGCTAACGATACTGCAACTTATTACTGT

>Vκ8-3 F

CCAGACTCCAGCCTACATTGCTGTACCCCTAGGAGAGAGCATCTCCATCACTTGCAGAGCCAATCAAAGCATTAGTGATTACTTAAGCTGGCATAAGCAGAAACCTGGCCAGGCTCCTATGATTCTCATCTATGATGCTGATAATCGTCTTAATGGTGTCCCAGAGAGGTTCACTGCGACTCAATCTGGGACAGAATTTGTTTTCACAATCAGCCAGGTAGAGGCTGATGATGCTGCCATGTATTACTGC

>Vκ11-1 P

GACATCATGATGACCCAGTCTCAAAGTTCCTTGGCTGTGTCTGCAGGAGAGGAAGACCACCATCAACTGCAGGTCCAGCCAGAATCTTCTATACTTCAACCAGAAAACCTAGTTTGCCTGGTGCCAGCAGAAAC

>Vκ1-1 ORF

GACATCCAGGTGACCCAGTCTCCATCCTCCCTATCTGCATCTCTAGCAGACAAAGTCTCCATCACTTGCCAGGCCAGTCAGAACACTGACACTAAATTAGCCTGGTATCAACAGAAACCAAGGAAAGCTCCTAAGCTCCTCATCTATGCAGTATCCAGGTCGCCCTCCTGGTTCCCATCCCAGTTCAGTGGCAGTGGATTTGGGATAGATTTCACCCTCACCATCAGCAGCCTGAAGGCTGATGATATTGCAGCTTACTACTGT

>Jκ1

TTAATAGTTTTGGCCAAGGAACCAAGCTAGAGATCAAAA

>Jκ2

TTCACTTTCGGCCCAGGGACCAGAGTGGAGATCAAAC

>Jκ3

ATATGCATTCGGCGGTGGGACCAAGGTGGAAATCAAAC

>Jκ4

TGGATCATCTTTGGCCAAGGGACACATCTGGAGATTAGAC

>Cκ

GGTCTGATGCTCAGCCATCCGTCTTCCTCTTCAAACCATCTGAGGAACAGCTGAGGACCGGAACTGTCTCTGTCGTGTGCTTGGTGAATGATTTCTACCCCAAAGATATCAATGTCAAGTGGAAAGTGGATGGGGTTACCCAGAACAGCAACTTCCAGAACAGCTTCACAGACCAGGACAGCAAGAAAAGCACCTACAGCCTCAGCAGCACCCTGACACTGTCCAGCTCAGAGTACCAGAGCCATAACGCCTATGCGTGTGAGGTCAGCCACAAGAGCCTGCCCACCGCCCTCGTCAAGAGCTTCAATAAGAATGAATGTTAG

>VL10-1 P

GCATCTTGGCTGCAGCCATACCCTGGCCAGGTCCCCAGACTCCTGACCCACAGGAGTCACAAGCAGTCCTTATCATCTCAGAGAAGTTCTCCAGTTCCAGGTCCGTCAAGACCCCTGGCCATCCCTGGGCTCCAACCTGAGGACGTGGCTGACTGTTTGTGC

>VL5-121 P

CAGCCTCTGCTGACTCAGCTGGCCTCCCTCTCTGCGTCTCCGGGAGCATCAGCCAGACTCACCTGCACCCTGAGCAGTGGCTACAGTGTTGGTAGCTACCAAATGTCCTGTTTCCAGCAGAAGCCAGGGGGCCCTCCCAAGTACCTCCTGATGTTCAAGCCAGAGTCGGATAAGCCCCAGGGCTCCAGGGTCCCCAGACACTTCTCTGGATCCAAGGCTGCCTCGGCCAACACAGAGCTCATGCTCATCTCTGGGCTGCAGACTCAGGATGAGGCTGATTGTAACTGC

>VL1-67 F

CTGTGCTGACTCAGCCGCCCTCTGTGTCCGGGTCCCTGGGCCAGACGGTGACCATCTCCTGCTCTGGAAGCAGCAGCAACATTGGACTATTGGGTGTGAGCTGGTACCAACAGCTCCCAGGATCGGCCCCCAAAACCCTGATCTATGGTAGTAACAAACGACCCTCGGGGGTCCCCGACCGATTCTCTGGCACCAAGTCTGGCAACACAGGCACCCTGACCATCACTTCGCTCCAGGCTGAGGACGAGGCCGATTATTACTGT

>VL5-119 F

CAGCCTCTTCTGACTCAGCCAGCCTCCCTCTCTGCGTCTCCGGGAGCATCAGCCAGACTCTCCTGCACCCTGAGCAGTGGCTACAATGTTGGCAACTATAGCATATACTGGTATCAGCAGAAGGCAGGGAGCCCTCCCCGGTACCTCCTGAGGTTCAAGTCAGACTCTGATAAGCACCAGGGCTCTGGGGTTCCCAACCGCTTCTCTGGATCCAAAGATGCCTCGACCAACGCAGGGCTCCTGCTCATCTCCGAGGTGCAGCCCGAGGACGAGGCCGACTATTACTGT

>VL1-83 P

CAGGCTGGGCTGACTCAGCTGCCCTCCTTTTCCGGGATACTGGGCCAGAGGGTCACCATCTCTTGTACTGGAAGCAGCAACATAATCGGGGGTTATTATGTGAGCTGGTACCAACAGCTCCCAGCATCGGCCCCCAGATTCCTGACCTACGAAAATGGCTAAAGATCCTCAGGGGTCCCGGATCGGTTCTCTGGCTCCAAGTCTGGCAGCTCGGCCTCTCTGACCACCTCAGTTCATGCTGAGGACGACGCTGATTACTGCTGC

>VL1-82 F

CAGGCCGTGCTGACTCAGCCACCCTCCGTGTCCGGGTCCCCGGGCCAGAGGGTATCCATCACCTGCTCTGGAAGCAGCAGCAACATCGGGGGTGGTAATTATGTGAGCTGGTTCCAACAGCTCCCAGGATCGGCCCCCAAACTCCTCATCTATGGTACTAGCAGTCGAGAGTCAGGGGTCCCAGACCGATTCTCTGGCTCCAGGTCTGGCAACACGGCCACCCTGACCATCAGCTCGCTCCAGGCTGAGGACGAGGCCGATTATTACTGT

>VL5-122 P

CCTGCACCCTGAGCAGTGACATCAGTGTTGGCCGCTGTGCTCTTACTGGAACCAGCAGAGGCCAGCGAGCCCTGCTCCGTCCTCCGGTCCTACCACTCAGACTCCAGGAAGAACCAAGCTCTGGGGTTCCCGCGGGTTTTCTGGATCCAAAGATGTCTCGGCCAGCGCAGGGCTCCTGCTCGTCTCTGGGCTGCAGCGTGAGGACGAGGCTGACTCTGACCGT

>VL8-74*1 P

CGGACTGTGGTCCAGGAGCCAGCGCTGTCAGTGTCTCCAGGAGGGACGGTCACCCTCACCTGTGGACTGAGCTCTGGGTCAGTCACTACCAGTAACTACCCCAGCTGGTTCCAGCAGACCCCAGGCCAGGCTCCCTGATTGCTTATCTACAGCACAAACAGCTGATATTCTGGGGCCCCTGATTGCTTCTCTGGCTCCATCTCTGGAAACAAAGCCACCCTCACCATCACCGGGGCCCAGCCCGAGGACGAAGCCGACTATTACTGT

>VL1-79 F

CAGGCTGTGCTGACTCAGCCATCCTCCGTGTCCAGGTCCCTGGGCCAGAGGGTCACCATCACCTGCTCTGGAAGCAGCAGCAACACTGGGGGTAATTTTGTGGGCTGGTGCCAGCAGCTCCCAGGAACGGCCCCCAAAACCCGGATCTATGGTGATAGCAATCGACCCTCGGGAGGCCCAGAGTGGTTCTCTGGCTCCAAGTCTGGAAATTCGGCCTCCATGACCATCGCTTCACTCCCGGCGGAGGAGGAGGCTGGTTATTACTGT

>VL5-113*1 F

CAGCCTCTGCTGACTCAGCCGGCCTCCCTCTCTGGGTCTCCGGGAGCATCAGCCAGACTCACCTGCACCCTGAGCAGTGGCTACAGTGTTGTCAGTTATACCATATACTGGTATCAGCAGAAGGCAGGGAGCCCTCCCCGGTACCTCCTGAGGTTCAAGTCAGACTCGGATAAGCACCAGGGCTCCGGGGTTCCCAGCCACTTCTCTGGATCCAAAGATGCCTCGACCAACGCAGGGCTCCTGCTCATCTCTGGGCTGCAGCCCGAGGACGAGGCCAACTATCACTGT

>VL5-123 P

CCTGCACCCTCAGCAGTGACATCAGTGTCGGCGGCTCTGCTGTTGCTGGAATCAGCAGAAGCCAGCGACCCCTCCTCAGTCCTCCGGTCCTGCCACTCAGACTCCAGGAAGAACCAGGCTCCAGGGGCCCCGTGTGTTTTCTGGATCCAAAGATGCCTCGGCCAGCGCAGGGCTCCTGCTCGTCTCTGGGCTGCAGCGTGAGGACGAGGCTGACTCTGACCGT

>VL1-121 P

CAGGCTCTGCTGACTCAGCCGCCCTCCGTGTCCAGGACTTTGGGCCAGAGGATCACCCTCTCCCGCACTGGAAGCAGCAGCAACACTGGGGTTATTATGTGAGCTGGCACCAACAGCTCCCAGGATCGGCGCCCACACTTCTGATCTATGAAAATGGCTAAAGATCCTCAGGGGTCCCGGATCGGTTCTCTGGCTCCAAGTCTGGCAGCTCGGCCTCTCTGACCATCTCAGTTCAGACTGAGGATGATGCTGATTATTGCTGT

>VL1-36*1 F

CAGGCTGTGCTGACTCAGCCGTCCTCCGTGTCCGGGTCCCCGGGCCAGAGGGTCTCCATCACCTGCTCTGGAAGCAACATCGGTAGTAGTGGTGTAGGCTGGTTCCAACAGCTCCCAGGATCGGGCCTCAAAACCGTCATCTATTATAATAGCAATCGACCCTCAGGGGTCCCCGACCGATTCTCTGGCTCCAAATCGGGCAACACAGCCACCCTGACCATCAGCTCGCTCCAGGCTGAGGACGAGGCTGATTATTTCTGT

>VL10-2 P

GCCACACTCACCTGCCTGGGAACAGCAATACTGTTGGCACCAAGGGGGCGGCTTGGCTGCCATCTCAGTCATGCTGAGTCCCAAGCTCTGAGCACAGGAGTCACAACCTGCCCGCAGGGTCTCAGCAAGGCTGGCAGGCTCTGATTTGGGTG

>VL5-124 P

CCTGCACCCTCAACAGTGACATCAGTGTCGGCCACTCTGCTCTTACTGGAACCAGCAGAAGCCAGCGAGTCCTCCTCAGTCCTACCACTCAGACTCCAGGAAGAACCAGGCTCTGGGGTCCCTGCGAGTTTTCTGGATCCAAAGGTGTCTCGGCCAACTCAGGGCTCCTACTCGTCTCTGGGCTGCAGCGTGAGGACGACGCTGACTCTGACCGT

>VL1-120 F

CTGTGCTGACTCAACCGCCGTCCGTGTCCGGGTCCCTGGGCCAGACGGTGACCATCTCCTGCTCTGGAAGCAGCAGCAACATTGGACTATTGGGTGTGAGCTGGTACCAACAGCTCCCAGGATCGGCCCCCAGAACCCTGATCTATGGTAGTAACAAACGACCCTCGGGGGTCCCCGACCGATTCTCTGGCACCAAGTCTGGCAACACAGGCACCCTGACCATCACTTCGCTCCAGGCTGAGGACGAGGCCGATTATTACTGT

>VL5-84 F

CAGCCTCTTCTGACTCAGCCGGCCTCCCTCTCTGCGTCTCCGGGAGCATCAGCCAGACTCTCCTGCACCCTGAGCAGTGGCTACAATGTTGGCAACTATAGCATATACTGGTATCAGCAGAAGGCAGGGAGCCCTCCCCGGTACCTCCTGAGGTTCAAGTCAGACTCTGATAAGCACCAGGGCTCTGGGGTTCCCAACCGCTTCTCTGGATCCAAAGATGCCTCGACCAACGCAGGGCTCCTGCTCATCTCCGAGGTGCAGCCCGAGGACGAGGCCGACTATTACTGT

>VL1-118 P

CAGGCTGTGCTGACTCAGCTGCCCTCCTTTTCCGGGATATTGGGCCAGAGGGTCACCATCTCTTGTACTGGAAGCAGTAACAAAATCGGGGGTTATTATGTGAGCTGGTACCAACAGCTCCCAGCATCGGGCCCCAGACTCCTGACCTATGGAAATGGCTAAAGACCCTCAGGGGTCCCAGATCGGTTCTCTGTCTCCAAGTCTGGCAGCTCGGCCTCTCTGACCACTGCAGTTCATGCTGAGGACGACGCTGATTATTGCTGC

>VL1-117*1 F

CAGGCTGTGCTGACTCAGCCGTCCTCCGTGTCCGGGTCCCTGGGCCAGAGGGTCTCCATCACCTGCTCTGGAAGCAGCAGCAACATCGGGGGTGGTAATTATGTGGGCTGGTACCAACAGCTCCCAGGATCAGGCCTCAAAACCATCATCTATGGTACTAGCAGTCGACCCTCGGGGGTCCCGGACCGATTCTCCGGCTCCAGGTCTGGCAACACGGCCACCCTGACCATCAGCTCGCTCCAGGCTGAGGACGAGGCCGATTATTACTGT

>VL5-125 P

CCTGCACCCTGAGCAGTGACATCAGTGTCGGCCGCTCTGCTCTTACTGGAGCCAGCAGAAGCCAGCGAGCCCTCCTCAGTCCTCCGGTCCTACCACTCAGACTCCAGGAAGAACCAGGCTCCGGGGTTCCCGCGGGTTTTCTGGATCCAAAGATGTCTCGGCCAGCACAGGGCTCCTGCTCGTCTCAGGGCTACAGCGTGAGGACGAGGCTGACTCTGACCGT

>VL8-109 P

GTGATCCAGGAGCCAGCTGTCAGTGTCTCCAGGAGGGACGGTCACCCTCACCTGTGGACTGAGCTCTGGGTCAGTCACTACCAGTAACTACCCCAGCTGGTTCCAGCAGACCCCAGGCCAGGCTCCCTGACTGCTTATCTACAGCACAAGCAGCTGATATTCTGGGGCCCCTGATTGCTTCTCTGGCTCCATCTCTGGAAACAAAGCCGCCCTCACCGTCACCGGGGCCCAGCACGAGGACGAGCCCGACTATTA

>VL1-59*1 F

CAGGCTGTGCTGACTCAGCCATCCTCCGTGTCCAGGTCCCTGGGCCAGAGGGTCACCATCACCTGCTCTGGAAGCAGCAGCAACATGGGGGGTAATTTTGTGGGCTGGTGCCAGCAGCTCCCAGGAACGGCCCCCAAAACCCCGATCTATGGTGATAGCAATCGACCCTCAGGGGGCCCAGAGTGGTTCTCTGGCTCCAAGTCTGGAAATTCGGCCTCCATGACCATCGCTTCACTCCAGTCGGAGGAGGAGGCTGGTTATTACTGT

>VL5-113*2 P

CAGCCTCTGCTGACTCAGCCGGCCTCCCTCTCTGGGTCTCCGGGAGCATCAGCCAGACTCACCTGCACCCTGAGCAGTGGCTACAGTGTTGGCAGTTATACCATATACTGGTATCAGTAGAAGGCAGGGAGCCCTCCCCGGTACCTCCTGAGGTTCAAGTCAGACTCGGATAAGCACCAGGGCTCCGGGGTTCCAAGCCACTTCTCTGGATCCAAAGATGCCTCGACCAACGCAGGGCTCCTGCTCATCTCTGGGCTGCAGCCCGAGGACGAGGCCGACTATCACTG

>VL5-126 P

CCTGCACCCTCAGCAGTGACATCAGTGTCGGCGGCTCTGCTGTTGCTGGAATCAGCAGAAGCCAGCGACCCTCCTCAGTCCTCCGGTCCTGCCACTCAGACTCCAGGAAGAACCAGGCTCCAGGGGCCCCGTGTGTTTTCTGGATCCAAAGATGCCTCGGCCAGCGCAGGGCTCCTGCTCGTCTCTGGGCTGCAGCGTGAGGACGAGGCTGACTCTGACCGT

>VL1-122 P

CAGGCTCTGCTGACTCAGCCGCCCTCCGTGTCCAGGACTTTGGGCCAGAGGATCACCCTCTCCCGCACTGGAAGCAGCAGCAACACTGGGGTTATTATGTGAGCTGGCACCAACAGCTCCCAGGATCGGCGCCCACACTTCTGATCTATGAAATGGCTAAAGATCCTCAGGGGTCCCGGATCGGTTCTCTGGCTCCAAGTCTGGCAGCTCGGCCTCTCTGACCATCTCAGTTCAGACTGAGGATGATGCTGATTATTGCTGT

>VL1-110 F

CAGGCTGTGCTGACTCAGCCGTCCTCCGTGTCTGGGTCCCTGGGCCAGAGGGTCTCCATCACCTGCTCTGGAAGCAGCAGCAACGTTGGTAGATATGGTGTAGGCTGGTACCAACAACTCCCAGGATCAGGCCTCAGAACCATCATCTATGGTACTAGCAGTCGACCCTCGGGGGTCCCGGACCGATTCTCCGGCTCCAGGTCTGGCAACACGGCCACCCTGACCATCAGCTCGCTCCAGGCTGAGGACGAGGCCAATTATTACTGT

>VL5-127 P

CCTGCACCCTGAGCAGTGACATCAGTGTCAGCCGCTCTGCTCTTACTGGAGCCAGCAGAAGCCAGCGAGCCCTCCTCAGTCCTCAGTCCTACCACTCAGACTCCAGGAAGAACCAGGCTCTGGGGTCCGTGGGTTTTCTGGATCCAAAGATGCCTCGGCCAACGCAGGGCTCCTGCTCGTCTCTGGGTTGCAGCGTGAGGACGAGGCTGACTCTGACCGT

>VL8-108*1 P

CAGAGTGTGGTCCAGGAGCCAGCACTGTCAGTGTCTCCATGAGGGATGGTCACCCTCACCTGTGGACTGAGCTCTGGGCCAGTCACTACCAGTAACTACCCCAGCTGGTTCCAGCAGACCCCAGGCCAGGCTCCCTGACTGCTTATCTACAGCACAAGCAGCTGATATTCTGGGGCCCCTGATTCCTTCTCTGGCTCCATCTCTGGAAACAAAGCCACCCTCACCGTCACCGGGGCCCAGCCCGAGGACAAAGCCAACTATTACTGT

>VL1-107 F

CAGGCTGTGGTGACTCAGCCATCCTCCGTGTCCGGGTCCCTGGGCCAGAGGGTCACCATCACCTGCTCTGGAAGCAGCAGCAACACTGGGGGTAATTTTGTGGGCTGGTGCCAGCAGCTCCCAGGAACGGCCCCCAAAACCCCGATCTATGGTGATAGCAATCGACCCTCGGGGGGCCCAGAGTGGTTCTCTGGCTCCAAGTCTGGAAATTCGGCCTCCATGACCATCGCCTCACTCCAGGCGGAGGAGGAGGCTGGTTATTACTGT

>VL5-106*1 P

CAGCCTCTGCTGACTCAGCCGGCCTCCCTCTCTGCATCTCCAGGAGCATCAGCCAGACTCTCCTGCACCCTGAGCAGTGGCTACAGTGTTGGCAGTTATACCATATACTGGTATCAGTAGAAGGCAGGGAGCCCTCCCCGGTACCTCCTGAGGTTCAAGTCAGACTCGGATAAGCACCAGGGCTCCGGGGTCCCCAGCCGCTTCTCTGGATCCAAAGATGCCTCGACCAACGCAGGGCTCCTGCTCATCTCTGGGCTGCAGCCCGAGGACGAGGCCGACTATCACTG

>VL1-105 F

CAGGCTGTGCTGACTCAGCCGTCCTCCGTGTCCAAGTCCCTGGGCCAGAGTGTCTCCATCACCTGCTCTGGAAGCAGCAGCAACGTTGGATATGGTAATTATGTGAGCTGGTTCCAACAGGTCCCAGGATCAGCCCCCAAACTCCTCATCTATGGTGCAACCAGTCGAGCCTCGGGGGTCCCCGACCGATTCTCCGGCTCCAGGTCTGGCAACACAGCGACTCTGACCATCAGCTCGCTCCAGGCTGAGGACGAGGCCGATTATTACTGT

>VL1-123 P

CAGGCTGTGCTGACTCAGCCACCCTGTTTCCTGAACTTTGGGTCAGAGGGTGACCATCTCCTGCACGGGAAGCAGCAACACAACCGGGGGTTATCATGTGAGCTGGTTCCAACAGCTCCCAGGATGGGCCCCAGACTCCTGACCTATGGAAATGGCTAAAGACCCTCAGGGGTCCCGGATCGGTTCTCTGTCTCCAAGTCTGGCAGCTCGGCCTCTCTGACCACCTCAGTTCATGCTGAGGACGACGCTGATTATTGCTGC

>VL1-103*1 F

CAGGCTGTGCTGACTCAGCCGTCCTCCGTGTCTGGGTCCCTGGGACAGAGGGTCTCCATCACCTGCTCTGGAAGCAGCAGCAACGTTGGATATGGTAATTATGTGGGCTGGTACCAACAGGTCCCAGGATCGGGCCTCAGAACCCTCATCTATGGTGCAACCAGTCGAGCCTCGGGGGTCCCCGACCGATTCTCCGGCTCCAGGTCTGGGAACACAGCCACCCTGACCATCAGCTCGCTCCAGGCTGAGGACGAGGCGGATTATTACTGT

>VL10-3 P

AGACAGCGAGCCGCACTCACCTGCACTGGAAAGCCCAGTACTGCTGGGCAGCAGCTTGGCTGCAGCAGCAACTGGGGGGTCCACAAAGCCCTGACCGCAGGAGCCACAACTGGCCCCCCGAGTCTCAGCGAGGCTCTCGGGCTCTGGGTCAGGCAGGGCCCCCTGACCATCTCTGGGCTCCAGCCCGAGGACGAGGCTGATTGTTCTTGC

>VL5-128 P

CAGCCTCTCCTAACTCAGCTGGCCTCCCTCTCTGCGTCTCCGGGAGCATCAGCCAGACTCACCTGCACCCTGAGCAGTGGGTACATTGTTGGTGAATATAGGATTTACTGATAGCAGTAGAAGCCAGGGACCCCTCTTCAGTACCTCCTGAGTTTCTTCTACTCAGAATCCAGTGAGCACCAGGACTCTGGGTTCCCAGCCGTGTCCCTGGATCCAAAGAAGCCTCGGCAAATGTAGGGTTCCTGCTCATCTCTGGGCCGCAGCCGGAGGAGGACACTGACTATTAC

>VL1-100*1 F

CAGGCTGTGCTGACTCAGCCGTCCTCTGTGTCCAGGTCCCTGGGCCAGAGTGTCTCCATCACCTGCTCTGGAAGCAGCAGCAACATCGGGGGTGGTTATTATGTGGGCTGGTGCCAGCAGCTCCCAGGATCGGCCCCCAGAACCCTCATCTATCAAAACAGCAAACGACCGACAGGCGTCCCCGACCGATTCTCTGGCTCCAAGTCGGGCAGCACAGCCACCCTGACCATCAGCTCGCTCCAGGCTGAGGACGAGGCGGATTATTACTGT

>VL5-129 P

CAGCCCGTGCTGACTCAGCTGACCTCCCTCTCTGCGTCTCTGGGAGAATCAGCCAGACTCATCTGCACCCTGAGCAGTGGGTATTGTGTTGGCGCCTATGCCAACAGAAGTCAGGGGTCTCCTCAGTAACTCCTGAGGTTCTACTCAGACTCCAATAAGTATCAAGGCTCCAGGGTCCAGACGTTTTTCTGGATCTAAGGCTGCCTCATCCAGTGCAGGGATCTCAGACCGTC

>VL1-98*1 F

CAGGCTGTGCTGACTCAGCCGTCCTCCGTGTCCGGGTCCCTGGGCCAGAGGGTCTCCATCACCTGCTCTGGAAGCAGCAGCAACATCGGGCGTGGTTATGGTAGCTGGTACCAACAGGTCCCAGGATCAGCCCCCAAACTCCTCATCTATGGTGCAACCAGTCGAGCCTCGGGGGTCCCCGACCGATTCTCTGGCTCCAGGTCTGGGAACACAGCCACCCTGACCATCAGCTCGCTCCAGGCTGAGGACGAGGCGGATTATTACTGT

>VL10-4 P

GCCACACTCACCCGCACTGGGACGAGCAAGAGTGTAGGCACCAAGGGGGCAGCTTGGCTGCAGCAACAACTGGGCAGGGTCCACAGACCCCTGACTACAGGAGTCACGACCAGCCCGCGGGGTCTCAGAGAGGCTCTAGGGCACTTAGCGCCAAGGACGAAGCTGA

>VL5-96*1 P

CAGCCTGTGCTGACTCAGCCGGCCTCCCTCTCTGGCTCTCCGGGAGCATCAGCCAGACTCTCCTGCACCCTGAGCAGCGGCTACAGTGTTGGTGATTTCTCCTTATCTTGGTTCCAGCAGAAGCCAGGAAGCCCTCCATGGTATGTCGTGAGGGTCAAGTCAGACTCCGATAAGAACCAAAGTTCTGGAGTCCCCAACCGCTTCTCTGGATCCAAAGATGCCTCAGCCAACGCAGGGCTCCTGCTCATCTCTGGGCTGCAGCCCGAGGACGAGGCCGACTATCACTG

>VL1-95*1 P

CAGGCTGGGCTGACTCAGCTGCCCTCCTTTTCCGGGATATTGGGCCAGAGGGTCACCATCTCTTGTACTGGAAGCAGCAACATAATCGGGGGTTATTATGTGAGCTGGTACCAACAGCTCCCAGGATGGGCCCCCAGACTCCTGACCTATGGAAATGGCTAAAGATCCTCAGGGGTCCCGGATCGGTTCTCTGGCTCCAAGTCTGGCAGCTCGGCCTCTCTGACCACCTCAGTTCATGCTGAGGACGACGCTGATTATTGCTGT

>VL1-94*1 F

CAGGCTGTGCCGACTCAGCCACCCTCTGTGTCCGGGTCCCCAGGCCAGAGGGTCTCCATCACCTGCTCTGGAAGCAGCAGCAACATCGGTAGCTATGGTGTATGTTGGTACCAACAGCTCCCAGGATCGGCCCCCAAACTCATCTGTTGTACTACCAGTGGAGCCTTGGAGTCCTCGGACCGATTCTCCTGCTCCAGGTCTGGCAACACGGCCACCCTGACCATCAGCTCGCTCCAGGCTGAGGACGAGGCCGATTATTACTGT

>VL5-130 P

CCTGCACCCTGAGCAGCGACATCAGTGTCTGCCGCTCTGCTCTTACTGGAACCAGCAGAAGCCAGTGAGCCCTCCTCAGTACTCTGGTCCTACCACTCAGACTCCAGGAAGAACCAGGCTCCGGGGTTCCCGCGGGTTTCCTGGATCCAAGGATGTCTCAGCCAACGCAGGGCTCCTGCTCGTCTCTGGGCTGCAGCCTGAGGACGAGGCTGACTCTGACCGT

>VL8-92*1 P

GTGATCCAGGAACCGTCACTGTTAGTGTCTCCAGGAGGGACGGTCACCCTCACCTGTGCACTGAGCTCTGGGTCAGTCACTACTTACAACGAACCCAGCTGGTACCAGCAGACCCCAGGCCAGGCTCCCAGAAATGTTATCTACAACACAAATACCCATGCCTCTGGGGTCCCTGATCGCTTCTCTGCCTCCATCTCTGGGAACAAAGCCACCCTCACCATCACGGGGGCCCAGCCCAAGGACGAGGCTGACGATCACTGC

>VL1-91*1 ORF

CAGGCTGTGCTGACTCAGCCACCCTCCGTGTCTGGATACCCAGGTCAGATGGTCACCATCTCCTGCACTGCAAGCAGCAGCAACATGAGGCTTGGCGATGTGGGCTGGTACCAACAGCTCCCCGGATCCGCTCCCAGAACCGTAATCTATGATACTATTGATCGACCTTCGGGGGTCCCAGACCGATTCTCTGGCTCCAGGTCTGACAGCACAGTCACCCTGACTATCACTTCGCTCCAGGCTGAGGACGAGGCAGATTATCACTGT

>VL10-5 P

TGATTCAGCCACATTCAGTGACTACGTCCTAGGACAGAAGGCCACTCTCACTTGGGCTGGAAAGATTGACCATGCTGTCAGCCAGGGGACAGCTTGGCTGCAGCCACATCCATGCTGGGTCCCTAAACTCCTAACCCTCGGGAGTCACAACTTGCCCCCAGAGTCTCAGAGAAGCTTTCGGGCCCCTGGTCAGGTGAAGCCCCCTGACCATCTCTGGGCTCTAGCCCGAGGATGAGGCTGATTGCCACCGC

>VL5-61 P

CAGCCTGTGCTGACTCAGTCAGCCTCCCTGTCTGGATCTCTGGGAGCATCAGCCAGACTCTCCTGCACCCTGAGCAGGGGCTACAACTTTGGCAGCTTGCCTATAACCTGGTACCAGCAGAAGCCAGGGAGTCCTCCCCGGTAACTCCTGTCCTACAACTCAGACTCCCAAAAGCTCCCGGGCTCCAGGGTCCCCAGCCACTTCTCTGGATCCAAAGACACCTCAGCCGACGTGGGGCGCCTGCTCATCTCTGGGCTGCAGCCCGAGGACAAGGCTGACTATTACTG

>VL8-108*2 P

GACTGTGGTCCAGGAGCCAGCTGTCAGTTTCTCCAGGAGGGATGGTCACCCTCACCTGTGGACTGAGCTCTGGGCCAGTCACTACCAGTAACTACCCCAGCTGGTTCCAGCAGACCCCAGGCCAGGCTCCCTGACTGCTTATCTACAGCACAAGCAGCTGATATTCTGGGGCCCCTGATTGCTTCTCTGGCTCCATCTCTGGGAACAAAGCCGCCCTCACCGTCACCGGGGCCCAGCCCGAGGACGAAGCCGACTATTACTGT

>VL1-73*1 F

CAGGCTGTGCTGACTCAGCCGTTCTCCGTGTCCGGGTCCCTGGGCCAGAGGGTCACCATCACCTGCTCTGGAAGCAGCAGCAACACTGGGGGTAATTTTGTGGGCTGGTGCCAGCAGCTCCCAGGAACGGACCCCAAAACCCCGATCTATGGTGATAGCAATCGACCCTCGGGGGGCCCAGAGTGGTTCTCTGGCTCCAAGTCTGGAAATTCCGCCTCCATGACCATCGCTTCACTCCAGGCGAAGGAGGAGGCTGGTTATTACTGT

>VL1-36*2 F

CAGGCTGTGCTGACTCAGCCGTCCTCCGTGTCCGGGTCCCTGGGCCAGAGGGTCTCCATCACCTGCTCTGGAAGCAACATCGGTAGTAGTGGTGTAGGCTGGTTCCAACAGCTCCCAGGATCGGGCCTCAGAACCGTCATCTATTATAATAGCAATCGACCCTCAGGGGTCCCCGACCGATTCTCTGGCTCCAAGTCGGGCAACACAGCCACCCTGACCATCAGCTCGCTCCAGGCTGAGGACGAGGCCGATTATTTCTGT

>VL10-6 P

GCCACACTCACCTGCCTGGGAACAGCAACACTGTTGGCACCAAGGGGGCGGCTTGGCTGCCATCTCAGTGGTGCTGAGTCCCAAGCTCTGAGCACAGGAGTCACAACCCGCCCACAGGGTCTCAGCGAGGCTGGCGGGCTCTGATTTGGATGGGGCCCCCTGACCGTC

>VL5-131 P

CCTGCACCCTCAGCAGTGACATCAGTGTCGGCCACTTTGCTGTTACTGGCACCAGCAGAAGCCAGCGAGCCCTCCTTGGTCCTCCAGTCCTACCACTCAGACTCCAGAAAGAACCAGGCTCCGGGGTCCCCACAGGTTTTCTGGATCCAAAGATGCCTTGGCCAACGCAGGGCTCCTGCTCGTCTCTGGGCTGCAGCGTGAGGACGACGCTGACTCTGACCGT

>VL9-1 P

CAGCCTGTGCTGATGCAGCCGCCCTGAGCATCTGCCTCCCTGGGAGCCTCGGCCAAACTCACCTGCACCCTGAGCGGCGGCTACAGCCGCTACTATGTGGGCTGGCACCAGAAGGTCCCAGGGAGGGGTCCCTGTTTCCTGATGTGAGTGGGCACCAGTGGTGTTGCGGGACAAGTGGGATGGGATCTCTGACCGCTTCTCAGGCTCGGGCTCTGGCCTGAGGGTGCCTGACCTTCCAGAACATCCCGGAGGAAGACGAGGCTGACTACATCTGT

>VL5-132 P

CAGCCTGAGGTGACTCAGGCGGCCTCCCTCTGGGCATCTCCGGGAGCATCAGCCAGACTCCCAGACTGCACCCTGCACAGTGGGCCAATGTTGGTGGCTAGCAAATATCCTGTTTCCAGCAGAAGCCAGGGAGCCCTCTCCAGGACCTCCTGGTGTTCAAGTCAGACTCCAATAAGCCCCAGGCCTCCGGGGTCCCCAGTCACTTCTGTGGATGCAAGGCTGCCTCAGCCAGTGAAGGGCTCCTGCTCATCTCTGGGCTGCAGACTCAGGATGAGGCTGATTATGATTGC

>VL1-40*1 F

CAGGCTGTGCTGACTCAGCCGTCCTCCGTGTCCAGGTCCCTGGGCCAGAGTGTCTCCATCACCTGCTCTGGAAGCAGCAGCAACGTTGGATATGGTAATTATGTGAGCTGGTTCCAACTCATCCCAGGATCGGCCCCCAGAACCCTCATCTATGGTGCAACCAGTCGAGCCTCGGGGGTCCCCGACCGATTCTCCGGCTCCAGGTCTGGCAACACAGCGACTCTGACCATCAGCTCGCTCCAGGCTGAGGACGAGGCCGATTATTACTGT

>VL8-110 P

AGGAGCTGGCACTGTCAGTGTCTCCAGGAGGGACCGTCATCCTCACCTGTGCCCTTCGCTCTGGTTCAGCCTACTCTCCCACCCAAATGGAACCAGTAGCCCCCAGGCCAGGATCCCCACAGGCTTACCCACAGCACAAGCAGCCGCCCCTCTGGGGTCCCCTGTTGCTTCTCTGGCTCCATCTTTGGGAACAAAGCTGTCCTCACCATCACGGGAGCCCAGTCTGAGGATGAGGCCAAGTATCACTGT

>VL8-111 P

TGGTCCAGGAGCCAGCACTGTCAGTGTCTCCAGGAGGGACGGTCACCCTCACCTGGGGCCTCAGCTCTGGGTCAGTCACCACCAGTAACTCCCCTGGCTGGTTCCAGCAGACCCCAGGCCAGGCTCCCAGAACTGCTATCTAAGCACAAACACCCGCCTCTCTGGGGTCCCTGCTCGCTTCTCTGTCTCCGTCTCTGGGAACAAAGCCGCCCTCACCATCACGGGGGCCCAGCCCGGGGACGAGGCCGTCCCTTACTGT

>VL1-124 P

CAGGCTGTGCTGACTCAGCCACCCTCTGTTTCCCGAACTTTGGGTCAGAGGGTGACCATCTCCTGCACTGGAAGCAGCAACACAATTGGGGGTTATCATGTGAGCTGGTTCCAACAGCTCCCAGGATGGGCCCCCAGACTCCTGACCTATGGAAATGGCTAAAGACCCTCAGGGGTCCCGGATCGGTTCTCTGGTCCAAGTCTGGCAGCTCGGCCTCTCTGACCACCCCAGTTCATGCTGAGGATGATGTTGATTATTACTGT

>VL1-125 P

CAGGCTGTGCTGACTCAGCCGCCCTCCGTGTCCGGGTCCCCGGGCCAGAGGGTCTCCATCACCTGCTCTGGAAGCAGCAGCAACATCGGGGGTGGTAATTATGTGGGCTGGTACCAACAGCTCCCAGGATCAGGCCTCAAAACCATCATCTATGGTACTAGCAGTCGACCCTCGGGGGTCCCGGACCGATTCTCGGCTCCAGGTCTGGCAACACGGCCACCCTGACCATCAGCTCGCTCCAGGCTGAGGACGAGGCCGATTATTACTGT

>VL5-133 P

CCTGCACCCTGAGCAGTGACATCAGTGTCGGCCGCTCTGCTCTTACTGGAACCAGCAGAAGCCAGCGAGCCCTCCTCAGTCCTCCGGTCCTACCACTCAGACTCCAGGAAGAACCAGGCTCCGGGGTTCCCGCGGGTTTTCTGGATCCAAAGATGTCTCGGCCAGCACAGGGCTCCTGCTCGTCTCAGGGCTGCAGCGTGAGGACGAGGCTGACTCTGACCGT

>VL8-46 F

GTGATCCAGGAGCCAGCACTGTCAGTGTCTTCAGGAGGGATGGTCACCCTCACCTGTGCACTGAGCTCTGGGTCAGTCACTACTTACAACGAACCCAGCTGGTACCTGCAGACCCCAGGCCAGGCTCTCAGAAATGTTATCTACAACACAAACACCCGCCCCTCTGGGGTCCCTGATCGCTTCTCTGCCTCCATCTCTGGGAACAAAGCCACCCTCACCATCACGGGGGCCCAGCCCGAGGACGAGGCTGAGGATCACTGC

>VL9-2 P

CAGCCTGTGCTGACGCAGCCGCCCTGAGCATCTGCCTCCCTGGGAGCCTCGGCCAAACTCACCTGCACCCTGAGCGGCGGCTACAGCAGCTACTATGTGGGCTGGCACCAGAAGGTCCCAGGGAGGGGTCCCCGTTTCCTGATGCGAGTGGGCACCAGTGGTGTTGCTGGACAAGTGGGATGGGATCTCTGACCGCTTCTCAGGCTCGGGCTCTGGCCTGAGGGTGCCTGACCTTCCAGAACGTCCCGGAGGAGGGCGAGGCTGACTACATCCGT

>VL5-134 P

CAGCCTGTGGTGACTCAGCCAGCCTCCCTCTGGGCATCTCCGGGAGCATCAGCCAGACTCCCAGACTGCACCCTGCACAGTGGGCCAGTGTTGGTGGCTAGCAAATATCCTGTTTCCAGCAGAAGCCAGGGAGCCCTCTCCAGGACCTCCTGGTGTTCAAGTCAGACTCCAATAAGCCCCAGGCCTCCGGGGTCCCCAGTCACTTCTGTGGATGCAAGGCTGCCTCAGCCAGTGAAGGGCTCCTGCTCATCTCTGGGCTGCAGACTCAGGATGAGGCTGATTATGATTGC

>VL1-49 F

CAGGCTGTGCTGACTCAGCCGTCCTCCGTGTCCAGGTCCCTGGGCCAGAGTGTCTCCATCACCTGCTCTGGAAGCAGCAGCAACGTTGGATATGGTAATTATGTGGGCTGGTTCCAACAGGTTCCAGGATCAGCCCCCAAACTCCTCATCTATGGTGCAACCAGTCGAGCCTCGGGGGTCCCCGCCCGATTCTCCGGCTCCAGGTCTGGCAACACAGCGACTCTGACCATCAGCTCGCTCCAGGCTGAGGACGAGGCCGATTATTACTGT

>VL1-126 ORF

CAGGCTGTGCTGACTCAGCCACCCTGTTTCCCGAACTTTGGGTCAGAGGGTGACCATCTCCTGCACGGGAAGCAGCAACACAACCGGGGGTTATCATGTGAGCTGGTTCCAACAGCTCCCAGGATGGGCCCCAGACTCCTGACCTATGGAAATGGCTAAAGACCCTCAGGGGTCCCGGATCGGTTCTCTGGCTCCAAGTCTGGCAGCTCGGCCTCTCTGACCACCTCAGTTCATGCTGAGGACGACGCTGATTATTGCTGT

>VL1-103*2 ORF

CAGGCTGTGCTGACTCAGCCGTCCTCCGTGTCTGGGTCCCTGGGCCAGAGGGTCTCCATCACCTGCTCTGGAAGCAGCAGCAACGTTGGATATGGTAATTATGTGGGCTGGTACCAACAGGTCCCAGGATCAGGCCTCAGAACCCTCATCTATGGTGCAACCAGTCGAGCCTCGGGGGTCCCCGACCGATTCTCCGGCTCCAGGTCTGGGAACACAGCCACCCTGACCATCAGCTCGCTCCAGGCTGAGGACGAGGCGGATTATTACTGT

>VL10-7 P

AGACAGCGAGCCGCACTCACCTGCACTGGAAAGCCCAGTACTGCTGGGCAGCAGCTTGGCTGCAGCAGCAACTGGGGGGTCCACAAAGCCCTGACCGCAGGAGCCACAACTGGCCCCCCGAGTCTCAGCGAGGCTCTCGGGCTCTGGGTCAGGCAGGGCACCCTGACCATCTCTGGGCTCCAGCCCGAGGACGAGGCTGATTGTTCTTGC

>VL5-135 P

CAGCCTGTGCTGACTCAGCCGGCCTCCCTCTCTGCCTCTCCGGGAGCATCAGCCAGACTCACCTGCACCCTGAGCAGTGGGTACATTGTTGGTGAATATAGGATTTACTGATAGCAGTAGAAGCCAGGGACCCCTCTTCAGTACCTCCTGAGTTTCTTCTACTCAGAATCCAGTGAGCACCAGGACTCTGGGTTCCCAGCCGTGTCCCTGGATCCAAAGAAGCCTCGGCAAATGTAGGGTTCCTGCTCATCTCTGGGCCGCAGCCGGAGGAGGACACTGACTATTAC

>VL1-100*2 F

CTGTGCTGACTCAGCCGTCCTCTGTGTCCAGGTCCCTGGGCCAGAGTGTCTCCATCACCTGCTCTGGAAGCAGCAGCAACATCGGGGGTGGTTATTATGTGGGCTGGTGCCAGCAGCTCCCAGGATCGGCCCCCAGAACCCTCATCTATCAAAACAGCAAACGACCGACAGGCGTCCCCGACCGATTCTCTGGCTCCAAGTCGGGCAGCACAGCCACCCTGACCATCAGCTCGCTCCAGGCTGAGGACGAGGCGGATTATTACTGT

>VL5-136 P

CAGCCCGTGCTGACTCAGCTGACCTCCCTCTCTGCGTCTCTGGGAGAATCAGCCAGACTCATCTGCACCCTGAGCAGTGGGTATTGTGTTGGCGCCTATGCCAACAGAAGTCAGGGGTCTCCTCAGTAACTCCTGAGGTTCTACTCAGACTCCAATAAGTATCAAGGCTCCAGGGTCCAGACGTTTTTCTGGATCTAAGGCTGCCTCATCCAGTGCAGGGATCCTGCTCACCTCGGGGCTGCAGCTGAGGACGAGGCTGGCCAATACTGT

>VL1-98*2 ORF

CAGGCTGTGCTGACTCAGCCGTCCTCCGTGTCCGGGTCCCTGGGCCAGAGGGTCTCCATCACCTGCTCTGGAAGCAGCAGCAACATCGGGCGTGGTTATGGTAGCTGGTACCAACAGGTCCCAGGATCAGCCCCCAAACTCCTCATCTATGGTGCAACCAGTCGAGCCTCGGGGGTCCCCGACCGATTCTCTGGCTCCAGGTCTGGGAACACAGCCACCCTGACCATCAGCTCGCTCCAGGCTGAGGACGAGGCGGATTATTACTGT

>VL10-8 P

AGCCACACTCACCCGCACTGGGACGAGCAAGAGTGTAGGCACCAAGGGGGCAGCTTGGCTGCAGCAACAACTGGGCAGGGTCCACAGACCCCTGACTACAGGAGTCACGACCAGCCCGCGGGGGTCTCAGAGAGGCTCTAGGGCACTTAGCGCCAAGGACGAAGCTGA

>VL5-96*2 P

CAGCCTGTGCTGACTCAGCCGGCCTCCCTCTCTGCCTCTCCGGGAGCATCAGCCAGACTCTCCTGCACCCTGAGCAGCGGCTACAGTGTTGGTGATTTCTCCTTATCTTGGTTCCAGCAGAAGCCAGGAAGCCCTCCATGGTATGTCGTGAGGGTCAAGTCAGACTCCGATAAGAACCAAAGTTCTGGAGTCCCCAACCGCTTCTCTGGATCCAAAGATGCCTCAGCCAACGCAGGGCTCCTGCTCATCTCTGGGCTGCAGCCCGAGGACGAGGCCGACTATCACTG

>VL1-95*2 P

CTGGGCTGACTCAGCTGCCCTCCTTTTCCGGGATATTGGGCCAGAGGGTCACCATCTCTTGTACCGGAAGCAGCAACATAACCGGGGGTTATTATGTGAGCTGGTTCCAACAGCTCCCAGGATCGGCCCCCAGATTCCTGACCTATGGAAATGGCTAAAGATCCTCAGGGGTCCCGGATCGGTTCTCTGGCTCCAAGTCTGGCAGCTCAGCCTCTCTGACCACCTCAGTTCATGCTGAGGACGACGCTGATTATTGCTGT

>VL1-94*2 F

CAGGCTGTGCCGACTCAGCCACCCTCTGTGTCCGGGTCCCCGGGCCAGAGGGTCTCCATCACCTGCTCTGGAAGCAGCAGCAACATCGGTAGCTATGGTGTATGTTGGTACCAACAGCTCCCAGGATCGGCCCCCAAACTCATCTGTTGTACTACCAGTGGAGCCTTGGAGTCCTCGGACCGATTCTCCTGCTCCAGGTCTGGCAACACGGCCACCCTGACCATCAGCTCGCTCCAGGCTGAGGACGAGGCCGATTATTACTGT

>VL5-137 P

CCTGCACCCTGAGCAGCGACATCAGTGTCTGCCGCTCTGCTCTTACTGGAACCAGCAGAAGCCAGTGAGCCCTCCTCAGTACTCTGGTCCTACCACTCAGACTCCAGGAAGAACCAGGCTCCGGGGTTCCCGCGGGTTTCCTGGATCCAAGGATGTCTCAGCCAACGCAGGGCTCCTGCTCGTCTCTGGGCTGCAGCCTGAGGACGAGGCTGACTCTGACCGT

>VL8-92*2 P

GTGATCCAGGAACCGTCACTGTTAGTGTCTCCAGGAGGGACGGTCACCCTCACCTGTGCACTGAGCTCTGGGTCAGTCACTACTTACAACGAACCCAGCTGGTACCAGCAGACCCCAGGCCAGGCTCCCAGAAATGTTATCTACAACACAAATACCCATGCCTCTGGGGTCCCTGATCGCTTCTCTGCCTCCATCTCTGGGAACAAAGCCACCCTCACCATCACGGGGGCCCAGCCCAAGGACGAGGCTGACGATCACTGC

>VL1-91*2 F

CAGGCTGTGCTGACTCAGCCACCCTCCGTGTCTGGATACCCAGGTCAGATGGTCACCATCTCCTGCACTGCAAGCAGCAGCAACATGAGGCTTGGCGATGTGGGCTGGTACCAACAGCTCCCCGGATCCGCTCCCAGAACCGTAATCTATGATACTATTGATCGACCTTCGGGGGTCCCAGACCGATTCTCTGGCTCCAGGTCTGACAGCACAGTCACCCTGACTATCACTTCGCTCCAGGCTGAGGACGAGGCAGATTATCACTGT

>VL10-9 P

TGATTCAGCCACATTCAGTGACTACGTCCGAGGACAGAAGGCCACTCTCACTTGGGCTGGAAAGATTGACCATGCTGTCAGCCAGGGGACAGCTTGGCTGCAGCCACATCCATGCTGGGTCCCTAAGCTCCTAACCCTCGGGAGTCACAACTTGCCCCCAGAGTCTCAGAGAAGCTTTCGGGCCCCTGGTCAGGTGAAGCCCTCTGACCATCTCTGGGCTCTAGCCCGAGGATGAGGCTGATTGCCACCAC

>VL5-138 ORF

CAGCCTGTGCTGACTCAGTCAGCCTCCCTGTCTGGATCTCTGGGAGCATCAGCCAGACTCTCCTGCACCCTGAGCAGGGGCTACAACTTTGGCAGCTTGCCTATAACCTGGTACCAGCAGAAGCCAGGAGTCCTCCCGGTAACTCCTGTCCTACAACTCAGACTCCCAAAGCTCCCGGGCTCCAGGGTCCCCAGCCACTTCTCTGGATCCAAAGACACCTCAGCCGACGTGGGGCGCCTGCTCATCTCTGGGCTGCAGCCCGAGGACAAGGCTGACTATTACTGG

>VL8-112 P

GACTGTGGTCCAGGAGCCAGCTGTCAGTGTCTCCAGGAGGGATGGTCACCCTCACCTGTGGACTGAGCTCTGGGCCAGTCACTACCAGTAACTACCCCAGCTGGTTCCAGCAGACCCCAGGCCAGGCTCCTGACTGCTTATCTACAGCACAAGCAGCTGATATTCTGGGGCCCCTGATTGCTTCTCTGGCTCCATCTCTGGGAACAAAGCCGCCCTCACCGTCACCGGGGCCCAGCCCGAGGACGAAGCTGACCATTACTGT

>VL1-73*2 P

CAGGCTGTGCTGACTCAGCCGTTCTCCGTGTCCGGGTCCCTGGGCCAGAGGGTCACCATCACCTGCTCTGGAAGCAGCAGCAACACTGGGGGTAATTTTGTGGGCTGGTGCCAGCAGCTCCCAGGAACGGACCCCAAAACCCCGATCTATGGTGATAGCAATCGACCCTCGGGGTCCCAGAGTGGTTCTCTGGCTCCAAGTCTGGAAATTCGGCCTCCATGACCATCGCTTCACTCCAGGCGAAGGAGGAGGCTGGTTATTACTGT

>VL1-36*3 P

CAGGCTGTGCTGACTCAGCCGTCCTCCGTGTCTGGGTCCCTGGGCCAGAGGGTCTCCATCACCTGCTCTGGAAGCAACATCGGTAGTAGTGGTGTAGGCTGGTTCCAGCTCCCAGGATCGGGCCTCAGAACCGTCATCTATTATAATAGCAATCGACCCTCAGGGGTCCCGACCCATTCTCTGGCTCCAAATCGGGCAACACAGCCACCCTGACCATCAGCTCGCTCCAGGCTGAGGACGAGGC

>VL8-113 P

GACTGTGGTCAGGAGCCAGCTGTCAGTTTCTCCAGGAGGATGGTCACCCTCACCTGTGGACTGAGGCTCTGGGTCAGTCACTACAAGTAACTACCCCAGCTGGTTCCAGCAGACCCCAGGCCAGGCTCCTGACTGCTTATCTACAGCACAAGCAGCTGATATTCTGGGGCCCCTGATTGCTTCTCTGGCTCCATCTCTGGAAACAAAGCGCCTCACCGTCACGGGGCCCAGCGAGACAGCCGACTATTACTGT

>VL1-73*3 P

CTGTGCTGACTCAGCCGTTCTCCGTGTCCGGGTCCCTGGGCCAGAGGGTCACCATCACCTGCTCTGGGCAGCAGCAACACTGGGGGTAATTTTGTGGGCTGGTGCCAGCAGCTCCCAGAACGGACCCCAAAACCCCGATCTATGGTGATAGCAATCGACCCTCGGGGCCCAGAGTGGTTCTCTGGCTCCAAGTCTGGAAATTCGCCTCCATGACCATCGCCTTCCTCCAGGCGAAGGAGGAGGCTGGTTATTACTGT

>VL1-57 ORF

CAGGCTGTGCTGACTCAGCCGTCCTCCGTGTCCGGGTCCCCGGGCCAGAGGGTCTCCATCACCTGCTCTGGAAGCAACATCGGTAGTAGTGGTGTAGGCTGGTTCCAACAGCTCCCAGGATCGGGCCTCAGAACCGTCATCTATTATAATAGCAATCGACCCTCAGGGGTCCCCGACCGATTCTCTGGCTCCAAGTCGGGCAACACAGCCACCCTGACCATCAGCTCGCTCCAGGCTGAGGACGAGGCCGATTATTTCTGT

>VL10-10 P

GCCACACTCACCTGCCTGGGAACAGCAACACTGTTGGCACCAAGGGGCGGCTTGGCTGCCGTCTCAGTGGTGCTGAGTCCCAAGCTCTGAGCACAGGAGTCACAACCCGCCCACAGGGTCTCAGCGAGGCTGGCGGGCTCTGATTTGGATGGGGCCCCCTGACCGTC

>VL5-139 P

CCTGCACCCTCAGCAGTGACATCAGTGTCGGCCACTTTGCTGTTACTGGCACCAGCAGAAGCCAGCGAGCCCTCCTTGGTCCTCCAGTCCTACCACTCAGACTCCAGAAAGAACCAGGCTCCGGGGTCCCACAGGTTTTCTGGATCCAAAGATGCCTTGGCCAACGCAGGGCTCCTGCTCGTCTCTGGGCTGCAGCGTGAGGACGACGCTGACTCTGACCGT

>VL9-3 P

CAGCCTGTGCTGATGCAGCCGCCCTGAGCATCTGCCTCCCTGGGAGCCTCGGCCAAACTCACCTGCACCCTGAGCGGCGGCTACAGCCGCTACTATGTGGGCTGGCACCAGAAGGTCCCAGGGAGGGGTCCCTGTTTCCTGATGTGAGTGGGCACCAGTGGTGTTGCGGGACAAGTGGGATGGGATCTCTGACCGCTTCTCAGGCTCGGGCTCTGGCCTGAGGGTGCCTGACCTTCCAGAACATCCCGGAGGAAGACGAGGCTGACTACATCTGT

>VL5-140 P

CAGCCTGAGGTGACTCAGGCGGCCTCCCTCTGGGCATCTCCGGGAGCATCAGCCAGACTCCCAGACTGCACCCTGCACAGTGGGCCAATGTTGGTGGCTAGCAAATATCCTGTTTCCAGCAGAAGCCAGGGAGCCCTCTCCAGGACCTCCTGGTGTTCAAGTCAGACTCCAATAAGCCCCAGGCCTCCGGGGTCCCCAGTCACTTCTGTGGATGCAAGGCTGCCTCAGCCAGTGAAGGGCTCCTGCTCATCTCTGGGCTGCAGACTCAGGATGAGGCTGATTATGATTGC

>VL1-40*2 F

CAGGCTGTGCTGACTCAGCCGTCCTCCGTGTCCAGGTCCCTGGGCCAGAGTGTCTCCATCACCTGCTCTGGAAGCAGCAGCAACGTTGGATATGGTAATTATGTGAGCTGGTTCCAACTCATCCCAGGATCGGCCCCCAGAACCCTCATCTATGGTGCAACCAGTCGAGCCTCGGGGGTCCCCGACCGATTCTCCGGCTCCAGGTCTGGCAACACAGCGACTCTGACCATCAGCTCGCTCCAGGCTGAGGACGAGGCCGATTATTACTGT

>VL8-114 P

TCTAGGAGCTGGCACTGTCAGTGTCTCCAGGAGGGACCGTCATCCTCACCTGTGCCCTTCGCTCTGGTTCAGCCTACTCTCCCACCCAAATGGAACCAGTAGCCCCCAGGCCAGGATCCCCACAGGCTTACCCACAGCACAAGCAGCCGCCCCTCTGGGGTCCCCTGTTGCTTCTCTGGCTCCATCTTTGGGAACAAAGCTGTCCTCACCATCACGGGAGCCCAGTCTGAGGATGAGGCCAAGTATCACTGT

>VL8-115 P

CCAGGAGCCAGCACTGTCAGTGTCTCCAGGAGGGACGGTCACCCTCACCTGGGGCCTCAGCTCTGGGTCAGTCACCACCAGTAACTCCCCTGGCTGGTTCCAGCAGACCCCAGGCCAGGCTCCCAGAACTGCTATCTAAGCACAAACACCCGCCTCTCTGGGGTCCCTGCTCGCTTCTCTGTCTCCGTCTCTGGGAACAAAGCCGCCCTCACCATCACGGGGCCCAGCCCGGGACGAGGCCGTCCCTTACTGT

>VL1-127 P

CAGGCTGTGCTGACTCAGCCACCCTCTGTTTCCCGAACTTTGGGTCAGAGGGTGACCATCTCCTGCACTGGAAGCAGCAACACAATTGGGGGTTATCATGTGAGCTGGTTCCAACAGCTCCCAGGATGGGCCCCCAGACTCCTGACCTATGGAAATGGCTAAAGACCCTCAGGGGTCCCGGATCGGTTCTCTGGTCCAAGTCTGGCAGCTCGGCCTCTCTGACCACCCCAGTTCATGCTGAGGATGATGTTGATTATTACTGT

>VL1-117*2 ORF

CAGGCTGTGCTGACTCAGCCGCCCTCCGTGTCCGGGTCCCCGGGCCAGAGGGTCTCCATCACCTGCTCTGGAAGCAGCAGCAACATCGGGGGTGGTAATTATGTGGGCTGGTACCAACAGCTCCCAGGATCAGGCCTCAAAACCATCATCTATGGTACTAGCAGTCGACCCTCGGGGGTCCCGGACCGATTCTCCGGCTCCAGGTCTGGCAACACGGCCACCCTGACCATCAGCTCGCTCCAGGCTGAGGACGAGGCCGATTATTACTGT

>VL5-141 P

CCTGCACCCTGAGCAGTGACATCAGTGTCGGCCGCTCTGCTCTTACTGGAACCAGCAGAAGCCAGCGAGCCCTCCTCAGTCCTCCGGTCCTACCACTCAGACTCCAGGAAGAACCAGGCTCCGGGGTTCCCACGGGTTTTCTGGATCCAAAGATGTCTCGGCCAGCACAGGGCTCCTGCTCGTCTCAGGGCTACAGCGTGAGGACGAGGCTGACTCTGAC

>VL8-116 P

GTGATCCAGGAGCCAGCTGTCAGTGTCTCCAGGAGGGACGGTCACCCTCACCTGTGGACTGAGCTCTGGGTCAGTCACTACCAGTAACTAACCCAGCTGGTTCCAGCAGACCCCAGGCCAGGCTCCCTGACTGCTTATCTACAGCACAAGCAGCTGATATTCTGGGGCCCCTGATTGCTTCTCTGGCTCCATCTCTGGAAACAAAGCCGCCCTCACCGTCACCGGGGCCCAGCACGAGGACGAGCCCGACTATTA

>VL1-59*2 ORF

CAGGCTGTGCTGACTCAGCCATCCTCCGTGTCCAGGTCCCTGGGCCAGAGGGTCACCATCACCTGCTCTGGAAGCAGCAGCAACATGGGGGGTAATTTTGTGGGCTGGTGCCAGCAGCTCCCAGGAACGGCCCCCAAAACCCCGATCTATGGTGATAGCAATCGACCCTCGGGGGGCCCAGAGTGGTTCTCTGGCTCCAAGTCTGGAAATTCGGCCTCCATGACCATCGCTTCACTCCAGTCGGAGGAGGAGGCTGGTTATTACTGT

>VL5-106*2 P

CAGCCTCTGCTGACTCAGCCGGCCTCCCTCTCTGCATCTCCAGGAGCATCAGCCAGACTCACCTGCACCCTGAGCAGTGGCTACAGTGTTGGCAGTTATACCATATACTGGTATCAGTAGAAGGCAGGGAGCCCTCCCCGGTACCTCCTGAGGTTCAAGTCAGACTCGGATAAGCACCAGGGCTCCGGGGTTCCCAGCCACTTCTCTGGATCCAAAGATGCCTCGACCAACGCAGGGCTCCTGCTCATCTCTGGGCTGCAGCCCGAGGACGAGGCCGACTATCACTG

>VL5-142 P

CCTGCACCCTCAGCAGTGACATCAGTGTCGGCGGCTCTGCTGTTGCTGGAATCAGCAGAAGCCAGCGACCCCTCCTCAGTCCTCCGGTCCTGCCACTCAGACTCCAGGAAGAACCAGGCTCCAGGGGCCCCGTGTGTTTTCTGGATCCAAAGATGCCTCGGCCAGCGCAGGGCTCCTGCTCGTCTCTGGGCTGCAGCGTGAGGACGAGGCTGACTCTGACCGT

>VL1-128 P

CAGGCTCTGCTGACTCAGCCGCCCTCCGTGTCCAGGACTTTGGGCCAGAGGATCACCCTCTCCCGCACTGGAAGCAGCAGCAACACTGGGGTTATTATGTGAGCTGGCACCAACAGCTCCCAGGATCGGCGCCCACACTTCTGATCTATGAAAATGGCTAAAGATCCTCAGGGGTCCCGGATCGGTTCTCTGGCTCCAAGTCTGGCAGCTCGGCCTCTCTGACCATCTCAGTTCAGACTGAGGATGATGCTGATTATTGCTGT

>VL1-76 F

CAGGCTGTGCTGACTCAGCTGTCCTCCGTGTCTGGGTCCCTGGGCCAGAGGGTCTCCATCACCTGCTCTGGAAGCAGCAGCAACGTTGGTAGATATGGTGTAGGCTGGTTCCAACAGCTCCCAGGATCAGGCCTCAGAACCGTCATCTATGGTACTAGCAGTCGACCCTCGGGTGTCCCGGACCGATTCTCCGGCTCCAGGTCTGGCAACACGGCCACCCTGACCATCAGCTCGCTCCAGGCTGAGGACGAGGCCGATTATTACTGT

>VL5-143 P

CCTGCACCCTCAGCAGTGACATCAGTGTTGGCCGCTCTGCTCTTACTGGAGCCAGGAGAAGCCAGCGAGCCCTCCTCAGTCCTCCGGTCCTACCACTCAGACTCCAGGAAGAACCAGGCTCTGGGGTCCCGTGGGTTTTCTGGATCCAAAGATGCCTCGGCCAGCGCAGGGCTCCTACTCGTCTCTGGGCTGCAGCGTGAGGACGAGGCTGACTCTGACCGT

>VL8-74*2 P

GACTGTGGTCCAGGAGCCAGCGCTGTCAGTGTCTCCAGGAGGGACGGTCACCCTCACCTGTGGACTGAGCTCTGGGTCAGTCACTACCAGTAACTACCCCAGCTGGTTCCAGCAGACCCCAGGCCAGGCTCCCTGATTGCTTATCTACAGCACAAGCAGCTGATATTCTGGGGCCCCTGATTCCTTCTCTGGCTCCATCTCTGGAAACAAAGCCCCCCTCACCATCACCGGGGCCCAGCCCGAGGACGAAGCCGACTATTACTGT

>VL1-73*4 F

CAGGCTGTGCTGACTCAGCCGTTCTCCGTGTCCAGGTCCCTGGGCCAGAGGGTCACCATCACCTGCTCTGGAAGCAGCAGCAACATGGGGGGTAATTTTGTGGGCTGGTGCCAGCAGCTCCCAGGAACGGACCCCAAAACCCCGATCTATGGTGATAGCAATCGACCCTCGGGGGGCCCAGAGTGGTTCTCTGGCTCCAAGTCTGGAAATTCGGCCTCCATGACCATCGCTTCACTCCAGGCGAAGGAGGAGGCTGGTTATTACTGT

>VL1-33 F

CAGGCTGTGCTGACTCAGCCGTCCTCCGTGTCTGGGTCCCCGGGCCAGAGGGTCTCCATCACCTGCTCTGGAAGCTACATCGGTAGTAGTGGTGTAGGCTGGTTCCAACAGGTCCCAGGATCGGGCCTCAGAACCGTCATCTATGGTAGTACCAATCGACCCTCAGGGGTCCCCGACCGATTCTCTGGCTCCAAGTCGGGCAACACAGCCACCCTGACCATCAGCTCGCTCCAGGCTGAGGACGAGGCTGATTATTTCTGT

>VL10-11 P

CAGGCAGCATGGACTCAGCCAGGGTCGCTGATGCCATGGACGCTCAGCCACACTCACCTGCCTGGGAACAGCAATACTGTTGGCACCAAGGGGGCGGCTTGGCTGCCGTCTCAGTCATGCTGAGTCCCAAGCTCTGAGCACAGGAGTCACAACCCGCCCGCAGGGTCTCAGCGAGGCTGGCGGGCTCTGATTTGGGTG

>VL5-144 P

CTGCACCCTCAGCAGTGACATCAGTGTCTGCCACTCTGCTCTTACTGGAACCAGCAGAAGCCAGTGAGCCCTCCTCAGTCCTACCACTCAGACTCCAGGAAGAACCAGGTTCTGGGGTCCCTGCGAGTTTTCTGGATCCAAAGGTGTCTCGGCCAACTCAGGGCTCCTGCTCGTCTCTGGACTGCAGCGTGAGGACGAGGCTGACT

>VL2-22 P

TGGTTAATCCCTTGTGTCTTTGTATCAAGAGCACCCAGGCTCTGCCCTAAAATCTCTGATTTATGATGTCAGTAAAAGGTCCTCAGGGGTCCTGTTTGCATCTCAGGCTCCAGCTCTGGCAACACGATCTCCCTGACCATCTCTGGGCTCCAGGCTGA

>VL2-19*1 F

CAGTCTGGCCTGACTCAGCCTGCCTCAGTGTCTGGGAATCCGGGACAGATGGTCAGCATCTCCTGTGCTGGAACCAGCAGTGATATTGGGGGTTATAATGGTGTGGGCTGGTACCAACAGCTCCCGGGCTCAGCCCCCAAAACTCTGATTTATAATGTCAACAAACGGCCCTCAGGGATCCCTGCTCGGTTCTCTGGCTCCAAGTCCGGGAACACAGCCACCCTGACCATCTCTGGGCTCCAGGCAGAGGACGAGGCCGACTATTACTGC

>VL2-21 F

CAGTCTGCCCTGACTCAGCCTGCCTCAGTGTCTGGGAATCCGGGACAGACGGTCACCATCTCCTGTACTGGCACCAGCAGTGACATTGGGGCTTATAATGGTGTGGGCTGGTACCAACAGCTCCCGGGCTCAGCCCCCAAAACTCTGATTTATGAAGTCAGTAAGAGGCCCTCAGGGGTCCCTGCTCGGTTCTCTGGCTCCAAGTCCGGGAATACAGCCACCCTGACCATCTCTGGGCTCCAGGCAGAGGACGAAGCCGACTATTACTGC

>VL2-20 F

CAGTCTGCCCTGACTCAGCCTGCCTCAGTGTCTGGGAATCCGGGACAGACGGTCACCATCTCCTGTGCTGGCACCAGCAGTGACATCGGGGGTTTTAACTATATTGGCTGGTACCAACAGCTCCCAGGCTCAGCCCCCAAAACTCTGATTTATGAAGTCAGTAAGAGGCCCTCAGGGATCCCTGCTCGGTTCTCTGGCTCCAAGTCCGGGAACACAGCCACCCTGACCATCTCTGGGCTCCAGGCAGAGGACGAGGCCGACTATTACTGC

>VL2-19*2 F

CAGTCTGCCCTGACTCAGCCTGCCTCAGTGTCTGGGAATCCAGGACAGACGGTCACCATCTCCTGTACTGGCACCAGCAGTGACATTGGCGGTTATAATGGTGTGGGCTGGTACCAACAGCTCCCGGGCTCAGCCCCCAAAACTCTGATTTATAATGTCAACAAACGGCCCTCAGGGATCCCTGCTCGGTTCTCTGGCTCCAAGTCCGGGAACACAGCCACCCTGACCATCTCTGGGCTCCAGGCAGAGGACGAGGCCGACTATTACTGC

>VL2-13*1 F

CAGTCTGCCCTGACTCAGCCTGCCTCAGTGTCTGGGAATCCAGGACAGACGGTCACCATCTCCTGTACTGGCACCAGCAGTGACATCGGGGGTTATAACTATATTGGCTGGTACCAACAGCTCCCGGGCTCAGCCCCCAAAACTCTGATTTATAATGTCAACAAACGGCCCTCAGGGATCCCTGCTCGGTTCTCTGGCTCCAAGTCCGGGAACACAGCCACCCTGACCATCTCTGGGCTCCAGGCAGAGGACGAGGCCGACTATTACTGC

>VL2-13*2 F

CAGTCTGCCCTGACTCAGCCTGCCTCAGTGTCTGGGAATCCAGGACAGACGGTCACCATCTCCTGTACTGGCACCAGCAGTGACATCGGGGGTTATAACTATATTGGCTGGTACCAACAGCTCCCGGGCTCAGCCCCCAAAACTCTGATTTATAATGTCAACAAACGGCCCTCAGGGATCCCTGCTCGGTTCTCTGGCTCCAAGTCCGGGAACACAGCCACCCTGACCATCTCTGGGCTCCAGGCAGAGGACGAGGCCGACTATTACTGC

>VL2-19*3 F

CAGTCTGCCCTGACTCAGCCTGCCTCAGTGTCTGGGAATCCAGGACAGACGGTCACCATCTCCTGTACTGGCACCAGCAGTGACATTGGCGGTTATAATGGTGTGGGCTGGTACCAACAGCTCCCGGGCTCAGCCCCCAAAACTCTGATTTATAATGTCAACAAACGGCCCTCAGGGATCCCTGCTCGGTTCTCTGGCTCCAAGTCCGGGAACACAGCCACCCTGACCATCTCTGGGCTCCAGGCAGAGGACGAGGCCGACTATTACTG

>VL2-10 F

CAGTCTGCCCTGACTCAGCCTGCCTCAGTGTCTGGGAATCCGGGACAGACGGTCACCATCTCCTGTACTGGCACCAGCAGTGACATCGGTGGTTATAGCTATGTTGGCTGGTACCAACAGCTCCCGGGCTCAGCCCCCAAAACTCTGATTTATAATGTCAACAAACGGCCCTCAGGGATCCCTGCTCGGTTCTCTGGCTCCAAGTCCGGGAACACAGCCACCCTGACCATCTCTGGGCTCCAGGCAGAGGACGAGGCTGACTATTACTG

>VL3-9 P

TCTTCTCAGCTGACTCAGCAGCCTGCGGTGTCCGTGTCCTTGGGACAGACGGCCAGCATCGCCTGCCAGGGAGGCGACTTAGGAAGCTCTTATGCTCACTGGTACCGGCAGAAGCCGGGCCAGGCCCCTATGCTGGTCATTTATGAGTTCAGTGAGAGGCCCTCGGGGATCCCTGACCGGTTCTCTGGCTCCAACTCGGGGAACACGGCCACCCTGACCATCCGCGGGGCCCGGACCGAGGACGAGGCCGACTATTATTGT

>VL3-8 F

TCCTATGAACTGACCCAGCCGACTTCAGTGTCGGTGGCCTTGGGACAGACGGCCAAGGTCACCTGCCAGGGAGACAACCTAGGAAGCTCTTATGTTCAGTGGCACCAGCAGAAGCCGGGCCAGGCCCCTGTGACGGTCATTTATCAGGATAGTAAGAGGCCCTCGGGGATCCCTGACCGGTTCTCTGGCTCCAACTCGGGGAACACGGCCACCCTGACCATCAGCGGGGCCCGGACCGAGGACGAGGCCGACTATTACTGT

>VL3-7 F

TCCTATGAACTGACCCAGCCGACTTCAGTGTCGGTGGCCTTGGGACAGATGGCCAAGGTCACCTGCTCGGGAGACCTATTAGACGAAAATTTTGCTCACTGGTACCAGCAGAAGCCGGGCCAGGCCCCTGTGCTGGTCATTTATCTGAATAGTGAGCGGGCCTCGGGGATCCCTGACCGGTTCTCCGGCTCCAGCTCAGGGAGCACGGCCACCCTGACCATCAGCGGGGTCCAGGCTGAGGACGAGGCCGACTATTTCTGT

>VL3-10 P

TCTTCTCAGCTGACTCAGCCGCCTGCGGTGTCCGTGTCCTTGGGACAGACGGCCAGCATCGCCTGCCAGGGAGACGACTTAGGAAGCTCTATGGTCACTGGTACCGGCAGAAGCCGGGCCAGGCCCCTGTGCCGGTCATTTATAAAGATAGTGAGCGGCCCTCAGGGATCCCTGACTGGTTCTCTGGCGGCTCCGCCTCCCGGCCCTCCAGGCAGGCTCTGCACCGGGGCATCCGCAGTGG

>VL9-4 P

GCCTGTGCTGACCCAGCCCCCATCTGCGTCCTCCTCCCTGGGAGGCTCGGCCAAGCTCACCTGCGCCCTGAGCAGTGAGCGCAGCACAGCCTACACTGAGTGGCATCAACAGAGTCCTGGGCAGGCCCCTCGGCATCTGATGAGGCTGACCAGTGACGGGAAAGTCACCCCGGGGGACGGCATCCCCGACCGCGCCTCCGGCTCCAGCTCCAGCTCTGGGGCTGACGGCTACCTGACCATCAGCAACCTCAGTCTGACGACGAGGCTGATTACATCTGT

>VL3-4 F

TCCTATGAACTGACCCAGCCGACTTCAGTGTCGGTGGCCTTGGGACAGACGGCCAAGATCACCTGCCAGGGAGACCTGCTGGATAAAAAATATACAGCTTGGTACCAGCAGAAGCCGGGCCAGGCTCCTGTGAAGGTCATTTGTAAAGACAGTGAGCGGCCTTCAGGGATCCTTGACCGGTTCTCTGGCTCCAGCTCAGGCAAAACAGCCACCCTGACCATCAGCGGGGCCCGGACCGAGGACGAGGCCGACTATTACTGT

>VL3-3 F

TCCTATGAACTGACCCAGCCGACTTCAGTGTCAGTGGTCTTGGGACAGACGGCCAAGGTCACCTGCTCGGGGGACCTGCTGGACGAACAGTATACTCAGTGGCACCAGCAGAAGCCGGGCCAGGCCCCTGAGCTGGTCATTTATGAAGACAGCAAGCGGCGCTCAGGGATCCCAGACCGGTTCTCTGGCTCCAGCTCCAGCAAAACAGCCATCTTGACCATCAGTGGGGTCCGTGCGGAGGATGAGGCCGACTATTACTGT

>VL3-2 F

TCTTCTCAGCTGACTCAGCAGCCTGCGGTGTCCGTGTCCTTGGGACAGACGGCCAGCATCGCCTGCCAGGGAGGCGACTTAGCATTTGCTAGTGTTAACTGGTACCAGCTGAAGCCGGGCCAGGCCCCTGTGACGGTCATTTATGGTGGTAGCGACCGGGCCTCAGGGATCCCTGACCGGTTCTCTGGCTCCAAATCAGACACCACGGCCACCCTGACCATCCGCGGGGCCCAGGCTGAGGACGAGGCCGACTATTACTGT

>VL3-11 P

TGACTCAGCCACCCTCGGCGTCAGTGTCCCCAGGACAGACGCCAGGATCACGTGTGGGGGGACCAGTGCTGGAGGTGAAAGCGTTCAGTGGCACCAGCAGAAGCCGGGCCGGCCCCTGCGCTGCTCACCTATGGAGACGATAACCGACCCGCGGGGGTCCCTGACCGGTTCTCTGGCGCCAACTCGGGGAGCACGGCCACCCTGACCACCAGCGGGGCCCGGGCCGAGGACGAGGCCGACTATGACTGT

>VL5-145 P

GCCCAACTGTCCTGCACCATCAGCCCCCATTACGCCATCGTCGGGGACCTCGGCGTGTCCTGGTATCAGCAGCGAGCAGGCAGCGCCCCCCGCCTGCTCCTCTACTACCGCTCAGAGGAGGACCAACACCGGGCCCCCGGCACCCCGGACCGCTTCTCCGCAGCTGCGGATGCAGCCCACAACACCTGCGTCCTGACCATCAGCCCCGTGCAGCCCGAAGATGACGCCGATTATTACTGC

>VL5-146 ORF

CAGCCCGTGCTGAGTCAGCCGCCTTCCGTGACCTCATTTCTGGGAGCCACCGTCCGCCTGGCCTGCACCCTGCGCGGTGACCACAACATCGGCCTTCACAACATCTACTGGTACCAGCAGAGGCCCGGCCACCCCCCGAGATTCCTGCTGAGATATTTCTCCCCCTCCAACAAGAGGCAGGGCCACCAGGTGCCCCCTCGCTTCTCCGGCTCCAAAGACCTGGCCAAGAACACGGGGTATTTGAGTATTGCTGAGCTGCAGGCCGAGGACGAGGCTGTCTATTTCTGT

>Jλ1

CTTCGTCTTAGGTGGCGGGACCCAGCTCACCGTCCTAG

>Jλ2

TGGTGTTTTCGGCAGCGGGACCAGGCTGACCGTCCTGG

>Jλ3

GTATGCCTTTGGCAGCGGGACCGAGGTCACCATCTCAG

>Cλ1

GTCGGCCCAAGTCCGCACCCTCGGTCACCCTGTTCCTGCCCTCCACGGAGGAGCTCAGTGCCAACAAGGCCACCGTGGTGTGTCTCATCAGCGACTTCTACCCGGGTAGCGTGACCGTGGCCTGAAAGGCAGATGGCAGCACCATCACTCGGAACGTGGAGACCACCCAGGCCTCCAAACTGAGCAACAGCAAGTACGCGGCCAGCAGCTACGTGACCCTGACGGGCAGCGAGTGGAACTCTAACAGCAGTTACAGCTGCGAGGTCACGCACGAGGGGAGCACCGTGACGAAGACAGTGAAGCCCTCAGAGTGTCCTTAG

>Cλ2

GTCAGCCCAAGTCCGCACCCTCGGTCACCCTGTTCCCGCCTTCCACGGAGGAGCTCAGTACCAACAAGGCCACCGTGGTGTGTCTCATCAACGACTTCTACCCGGGTAGCGTGAACGTGGTCTGGAAGGCAGATGGCAGCACCATCAATCAGAACGTGAAGACCACCCAGGCCTCCAAACAGAGCAACAGCAAGTACGCGGCCAGCAGCTACCTGACCCTGACGGGCAGCGAGTGGAAGTCTAAGAGCAGTTACACCTGCGAGGTCACGCACGAGGGGAGCACCGTGACGAAGACAGTGAAGCCCTCAGAGTGTTCTTAG
