# Supplementary material for: Annotation of gene loci and analysis of expression diversity in sheep immunoglobulin
Source: Front Immunol. 2025 Aug 29;16:1643380. doi: 10.3389/fimmu.2025.1643380 (PMC12425715; doi:10.3389/fimmu.2025.1643380)
Supplement: Supplementary 2 — The Expression of V(D)J in Sheep IgH and IgL. [file Table2.docx]

**Supplement 2 The Expression of V(D)J in Sheep IgH and IgL**

| **The Expression of IgH VDJ** | | | | |  | **The Expression of IgL(λ) VJ** | | | | |
| --- | --- | --- | --- | --- | --- | --- | --- | --- | --- | --- |
|  |  | S1 | S2 | S3 |  |  |  | S1 | S2 | S3 |
| IgHV | VH1S8 | 0.0% | 0.1% | 0.1% |  | IgλV | Vλ1-67 | 2.8% | 4.2% | 1.1% |
|  | VH1S1 | 58.1% | 67.2% | 40.5% |  |  | Vλ1-120 | 0.0% | 0.4% | 0.1% |
|  | VH1S5 | 12.6% | 19.7% | 15.6% |  |  | Vλ1-117 | 3.6% | 4.7% | 5.9% |
|  | VH1S4 | 29.2% | 13.0% | 43.8% |  |  | Vλ1-110 | 2.1% | 0.7% | 4.1% |
|  |  | S1 | S2 | S3 |  |  | Vλ1-105 | 1.5% | 2.8% | 5.9% |
| IgHD | DH1 | 10.4% | 11.0% | 11.4% |  |  | Vλ1-103 | 11.1% | 12.2% | 14.2% |
|  | DH2 | 59.2% | 53.9% | 53.7% |  |  | Vλ1-100 | 0.2% | 1.0% | 0.0% |
|  | DH3 | 13.8% | 17.7% | 16.1% |  |  | Vλ1-98 | 2.0% | 5.3% | 1.8% |
|  | DH4 | 16.6% | 17.3% | 18.8% |  |  | Vλ1-94 | 0.0% | 0.8% | <0.1% |
|  |  | S1 | S2 | S3 |  |  | Vλ1-36 | 9.7% | 15.4% | 12.2% |
| IgHJ | JH4 | 88.3% | 91.1% | 86.5% |  |  | Vλ1-40 | 7.9% | 0.0% | 0.1% |
|  | JH6 | 11.7% | 8.9% | 13.5% |  |  | Vλ1-49 | 9.8% | 0.6% | 9.6% |
|  |  |  |  |  |  |  | Vλ1-94 | 0.2% | <0.1% | 0.2% |
| **The Expression of IgL(κ) VJ** | | | | |  |  | Vλ1-57 | 2.2% | 0.1% | 7.0% |
|  |  | S1 | S2 | S3 |  |  | Vλ1-76 | 0.9% | 6.2% | 1.2% |
| IgκV | Vκ2-15 | 0.6% | 0.1% | 0.3% |  |  | Vλ1-33 | 1.1% | 4.0% | 2.8% |
|  | Vκ2-14 | 1.5% | 12.8% | 1.6% |  |  | Vλ2-19 | 2.2% | 2.6% | 0.4% |
|  | Vκ2-8 | 43.4% | 11.7% | 40.1% |  |  | Vλ2-21 | 0.2% | 0.6% | 0.2% |
|  | Vκ1-4 | 54.5% | 75.4% | 57.9% |  |  | Vλ2-20 | 1.0% | 2.2% | 3.3% |
|  | Vκ8-3 | <0.0% | <0.0% | 0.1% |  |  | Vλ2-13 | 6.3% | 3.8% | 15.0% |
|  |  | S1 | S2 | S3 |  |  | Vλ2-10 | 2.6% | 14.7% | 10.0% |
| IgκJ | IgκJ1 | 62.1% | 62.7% | 57.8% |  |  | Vλ3-8 | 21.7% | 9.0% | 1.9% |
|  | IgκJ2 | 0.3% | 0.1% | 0.1% |  |  | Vλ3-7 | 0.4% | 0.3% | <0.1% |
|  | IgκJ3 | 37.6% | 37.2% | 42.1% |  |  | Vλ9-4 | 9.0% | 6.6% | 2.5% |
|  |  |  |  |  |  |  | Vλ3-3 | 0.1% | <0.1% | 0.0% |
|  |  |  |  |  |  |  | Vλ3-2 | 1.6% | 1.8% | 0.2% |
|  |  |  |  |  |  |  |  | S1 | S2 | S3 |
|  |  |  |  |  |  | IgλJ | Jλ1 | <0.1% | <0.1% | <0.1% |
|  |  |  |  |  |  |  | Jλ2 | >99.9% | >99.9% | >99.9% |
